# Oncological Efficacy and Safety of Minimally Invasive Focal and Whole-Gland Interventions in the Treatment of Low- and Intermediate-Risk Prostate Cancer: A Systematic Review and Meta-Analysis

**DOI:** 10.3390/cancers17172863

**Published:** 2025-08-30

**Authors:** Benjamin Skribek, Anett Szabó, Júlia Ács, Bianca Golzio Navarro Cavalcante, Boglárka Dorina Sipos, Péter Hegyi, Péter Mátrai, Péter Nyirády, Nándor Ács, Attila Majoros, Pál Ákos Deák

**Affiliations:** 1 Department of Interventional Radiology, Semmelweis University, 1122 Budapest, Hungary; skribek.benjamin@semmelweis.hu; 2 Centre for Translational Medicine, Semmelweis University, 1085 Budapest, Hungary; szabo.anett2@semmelweis.hu (A.S.); acs.julia@semmelweis.hu (J.Á.); bianca.golzio@phd.semmelweis.hu (B.G.N.C.); hegyi.peter@semmelweis.hu (P.H.); nyirady.peter@semmelweis.hu (P.N.); acs.nandor@semmelweis.hu (N.Á.); majoros.attila@semmelweis.hu (A.M.); 3 Department of Urology, Semmelweis University, 1082 Budapest, Hungary; 4 Department of Oral Biology, Semmelweis University, 1088 Budapest, Hungary; 5 Department of Radiology, Gottsegen National Cardiovascular Center, 1096 Budapest, Hungary; bogisipos1996@gmail.com; 6 Institute for Translational Medicine, Medical School, University of Pécs, 7624 Pécs, Hungary; peter.matrai@pte.hu; 7 Institute of Pancreatic Diseases, Semmelweis University, 1083 Budapest, Hungary; 8 Department of Obstetrics and Gynecology, Semmelweis University, 1088 Budapest, Hungary

**Keywords:** oncology, interventional radiology, localized prostate cancer, minimally invasive techniques

## Abstract

Background and objective: Minimally invasive interventions, including irreversible electroporation (IRE), cryoablation, and high-intensity focused ultrasound (HIFU), offer promising alternatives for the treatment of low- and intermediate-risk prostate cancer. We aimed to evaluate the oncological efficacy and safety of these treatments. Methods: A systematic search of MEDLINE, Central, and EMBASE was conducted up to 5 January 2025, according to the Preferred Reporting Items for Systematic Reviews and Meta-Analyses (PRISMA) guidelines. Recurrence, complication, survival, biochemical, and retreatment rates were evaluated, with risk of bias assessed using the Risk of Bias In Non-randomized Studies of Interventions (ROBINS-I) and Risk of Bias 2 (RoB2) tools. Results: 85 studies met the inclusion criteria, comprising 42 prospective cohort studies, 36 retrospective cohort studies, six registries, and one randomized controlled trial. Whole-gland HIFU showed significantly lower recurrence (15%) and postoperative mean PSA levels (0.68 ng/mL) than focal HIFU (24%, 2.81 ng/mL). Recurrence rates were similar for focal vs. extended IRE (30% vs. 26%) and focal vs. whole-gland cryoablation (18% vs. 13%). In-field and out-of-field recurrence rates were similar across treatment modalities (5–15%). Retreatment rates were low, with 6–7% of patients receiving a second ablation and 2–8% progressing to radical or hormonal therapy. Major complications were consistently rare. One-year biochemical recurrence-free survival (BRFS) exceeded 95%, and five-year BRFS approached 80% for HIFU and cryoablation. Conclusions: Minimally invasive focal and whole-gland therapies are safe and effective for treating low- and intermediate-risk prostate cancer, with high survival and low major complication rates. Notably, whole-gland HIFU achieves superior biochemical control and lower recurrence than focal HIFU, emphasizing the clinical importance of treatment extent.

## Simple Summary

The implementation of scientific knowledge plays a crucial role in delivering benefits to the community. This study provides a comprehensive synthesis of current evidence on minimally invasive treatments for low- and intermediate-risk prostate cancer, specifically comparing irreversible electroporation (IRE), cryoablation, and high-intensity focused ultrasound (HIFU). It demonstrates that both focal and whole-gland therapies are generally safe and effective, with high survival rates and low rates of major complications. Importantly, the study reveals that whole-gland HIFU achieves significantly lower recurrence and better biochemical control than focal HIFU. These findings highlight the potential benefits of broader treatment coverage in achieving optimal oncological outcomes.

## 1. Introduction

The prevalence of prostate cancer (PCa) is 5% below the age of 30 years, increasing by an odds ratio (OR) of 1.7 per decade up to 59% over the age of 79 years, consistent with autopsy findings [1]. Localized PCa is usually asymptomatic, but local progression may cause erectile dysfunction, urinary retention, pain, or hematuria. On the basis of patient eligibility, the first option for PCa management is active surveillance (AS), which aims to delay or avoid radical treatment [2]. These patients are closely monitored through regular follow-ups, including prostate-specific antigen (PSA) screening, clinical examinations, magnetic resonance imaging (MRI), and possibly biopsies [3,4,5]. One of the most significant prospective studies on low-risk PCa managed by AS, conducted by Tosoian et al., followed 1298 men and reported a median treatment-free survival of 8.5 years, alongside a 31% cumulative incidence of grade reclassification during the follow-up period [6]. In cases where patients are not eligible for AS, radical prostatectomy (RP) or radiotherapy (RT) is considered to be the next therapeutic option [7,8]. However, all radical treatments can have significant side effects, commonly including incontinence, erectile dysfunction, and infection; therefore, alternative options are essential [9]. Decision regret is lowest for AS (13%), followed by RP (18%) and RT (19%) [10].

Alternative therapeutic approaches include minimally invasive procedures such as high-intensity focused ultrasound (HIFU), microwave and radiofrequency ablation, cryoablation, and irreversible electroporation (IRE), aimed to reduce complications and toxicity with equivalent oncological effectiveness [11,12]. Focal therapy (FT) targets specific lesions and areas within the prostate to preserve healthy tissue and reduce side effects, whereas whole-gland treatments involve the complete ablation of the prostate [13,14].

This review aims to summarize the oncological effectiveness and safety of these focal and whole-gland interventional therapies in the treatment of low-intermediate risk PCa. We performed a systematic review and meta-analysis of oncological, biochemical, and complication outcomes to provide an updated synthesis of evidence, clarifying the role of these emerging therapies alongside AS and radical treatments. Furthermore, we conducted a subgroup analysis to assess the clinical differences between focal and whole-gland approaches.

## 2. Materials and Methods

During the selection and extraction stages, we followed the recommendations of the Cochrane Handbook, Preferred Reporting Items for Systematic Reviews and Meta-Analyses (PRISMA) statement (Supplementary Table S1) [15,16]. Our study protocol was registered on PROSPERO before the start date under registration number CRD42023414131.

### 2.1. Systematic Search

We systematically searched for relevant articles in three databases using consistent search terms: MEDLINE via PubMed, Central, and EMBASE. The search was conducted on 7 May 2023, and the last update was on 5 January 2025; the domains of our search key included prostate, low-intermediate-risk tumors, and minimally invasive interventional radiological treatments. Our search key is detailed in Supplementary Table S2.

No filters or other restrictions were used.

### 2.2. Eligibility Criteria

We formulated our clinical question using the PICO (Population, Intervention, Comparison, Outcome) framework. Studies included were identified according to the following criteria: (P) Patients who had received any interventional radiological treatment for their low- or intermediate-risk PCa; and (I, C) minimally invasive interventional procedures commonly used in clinical practice, including HIFU, IRE, radiofrequency ablation, microwave ablation, cryoablation, intravascular embolization, and chemical ablation, either separately or compared to one another. For IRE, focal and extended treatment strategies were compared, while for cryoablation and HIFU, our analysis examined focal approaches—including targeted, partial, quadrant, and hemigland ablation—in comparison to whole-gland ablation. (O) As for outcomes, biopsy-proven in-field recurrence, defined as cancer persisting or reappearing within the initially treated zone, and out-of-field recurrence, indicating cancer detected outside the treated area, were assessed using 6-month, 12-month, and pooled data when different follow-up times were not considered; complication rates were reported according to the Clavien–Dindo classification, with grade 3 or higher adverse events categorized as major complications; the survival endpoints evaluated comprised overall survival (OS), cancer-specific survival (CSS), and metastasis-free survival (MFS); functional outcomes, including postoperative urinary incontinence and erectile dysfunction; and biochemical outcomes were assessed based on postoperative mean PSA levels and biochemical recurrence-free survival rates (BRFS) as defined by the Phoenix criteria [17]. The study design consisted of randomized controlled trials (RCTs), prospective and retrospective cohort studies, case–control studies, and registries. No language restrictions were applied to the selection process.

Studies were excluded if they were (a) reviews, meta-analyses, systematic reviews, case reports, or case series; (b) preclinical or animal studies; (c) studies on low- and intermediate-risk patients could not be separated from high-risk cases or metastatic prostate tumors; and (d) studies on patients with previous treatment. Data from conference abstracts and papers without accessible full texts were also excluded.

### 2.3. Study Selection Process

**Figure 1:**
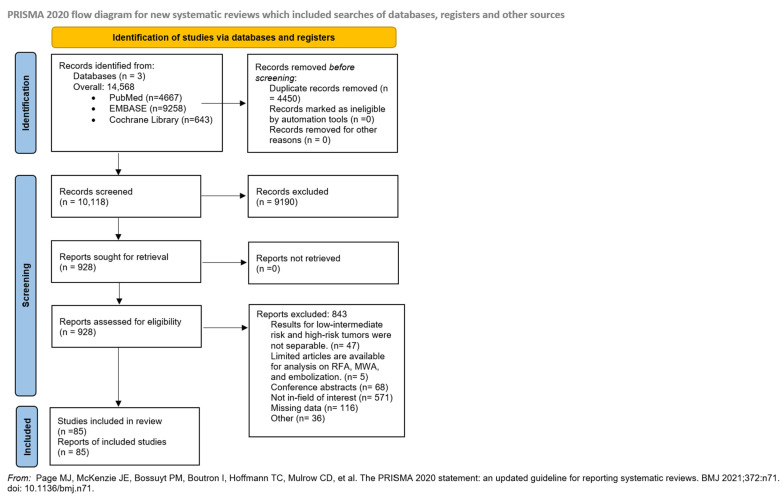
PRISMA 2020 flowchart showing the study selection process [16].

EndNote v9.0 (Clarivate Analytics, Philadelphia, PA, USA) reference manager software and Ryyan (Rayyan Systems Inc., Cambridge, MA 02142, USA) were used during the study selection process. After automatic and manual removal of duplicate records, two co-investigators (BS and JÁ) independently assessed the eligibility of articles by first author, title, and abstract, then the remaining articles by full text. A third investigator resolved any discrepancies (AS), and Cohen’s kappa coefficient (κ) was calculated at each stage to assess inter-rater reliability [18]. The entire study selection process is illustrated in the PRISMA flowchart (Figure 1).

### 2.4. Data Extraction

Two authors (BS and JÁ) independently extracted data from eligible publications using a standardized data extraction table. The data extracted included (a) general details of the article: name of the first author, year of publication, study design, study region, type and subgroups of interventions, and brand of the device used; (b) essential characteristics of the study population: age, tumor grading including Gleason score, National Comprehensive Cancer Network system (NCCN), and clinical Tumor Node Metastasis classification (TNM), and preoperative PSA levels; (c) outcome parameters included recurrence rates, biochemical control, retreatment rates, and complication rates according to Clavien–Dindo classification [19].

### 2.5. Risk of Bias Assessment

Two independent reviewers (BS and JÁ) assessed the risk of bias using the Risk of Bias In Non-randomized Studies of Interventions (ROBINS-I) tool for non-randomized studies and the Risk of Bias 2 (RoB 2) tool for randomized trials, following the guidelines outlined in the Cochrane Handbook [15,20,21]. For the risk assessment of the ROBINS-I and RoB 2 tools, pre-defined categories were established for each domain (Appendix A and Appendix B). Any disagreements were resolved by a third reviewer (BG).

### 2.6. Statistical Analysis

Statistical analyses were conducted with R software version 4.1.3, using the meta and metafor packages. All analyses employed a random effects model with Hartung–Knapp adjustments to minimize false positive conclusions [22]. We used the Q test and I^2^ statistics to evaluate statistical heterogeneity. Findings were presented in forest plots, with the mean effect size and its 95% confidence interval (CI) as summary statistics. Where possible, we included the 95% prediction interval (PI), following the recommendation of IntHout et al. [23]. Raw complication, recurrence, and survival rates were logit-transformed, pooled using the random effects model, and back-transformed for presentation on the original scale [24]. For the analysis of postoperative PSA levels, the mean PSA values were assessed. In cases where the mean and standard deviation were not directly reported, median values, quartiles, and minimum–maximum ranges were extracted, and the mean was estimated using the method proposed by Luo et al., while the standard deviation was calculated according to the method described by Shi et al. [25,26]. The leave-one-out method was used for sensitivity analysis. Statistical significance was set at *p* < 0.05.

## 3. Results

### 3.1. Search and Selection

A total of 14,568 articles were identified, with 10,118 remaining after duplicate removal. After title and abstract selection, 928 articles were found, and 85 full-text articles were eligible for analysis. (Figure 1). Our meta-analysis and systematic review included 82 single-arm studies, 14 studies on IRE [27,28,29,30,31,32,33,34,35,36,37,38,39,40], 28 on cryoablation [41,42,43,44,45,46,47,48,49,50,51,52,53,54,55,56,57,58,59,60,61,62,63,64,65,66,67,68], 40 on HIFU [69,70,71,72,73,74,75,76,77,78,79,80,81,82,83,84,85,86,87,88,89,90,91,92,93,94,95,96,97,98,99,100,101,102,103,104,105,106,107,108], and three comparative articles investigating both cryoablation and HIFU [109,110,111]. The eligibility criteria for each study are detailed in Supplementary Table S3.

### 3.2. Basic Characteristics of Included Studies

In terms of study design, 49.5% (42/85) of the articles included were prospective cohort studies, 42.5% (36/85) were retrospective cohort studies, 7% were registries (6/85), and 1% were randomized controlled trials (1/85). The baseline characteristics of these studies are detailed in Supplementary Tables S4–S6. A total of 15,488 patients with a mean age ranging from 60 to 74 years were included in our review, of whom 682 were treated with IRE, 7371 with cryoablation, and 7435 with HIFU worldwide.

### 3.3. Recurrence Rates

**Figure 2:**
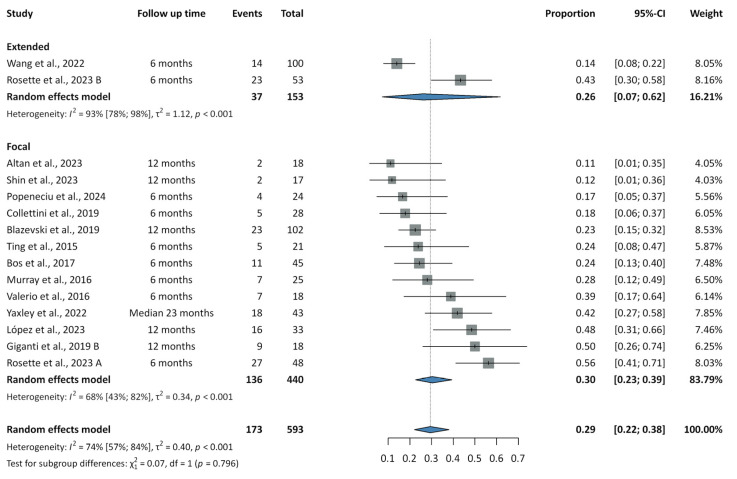
Summary forest plot of pooled recurrence rates for focal and extended irreversible electroporation [27,28,29,30,31,32,33,34,35,36,37,38,39,40].

**Figure 3:**
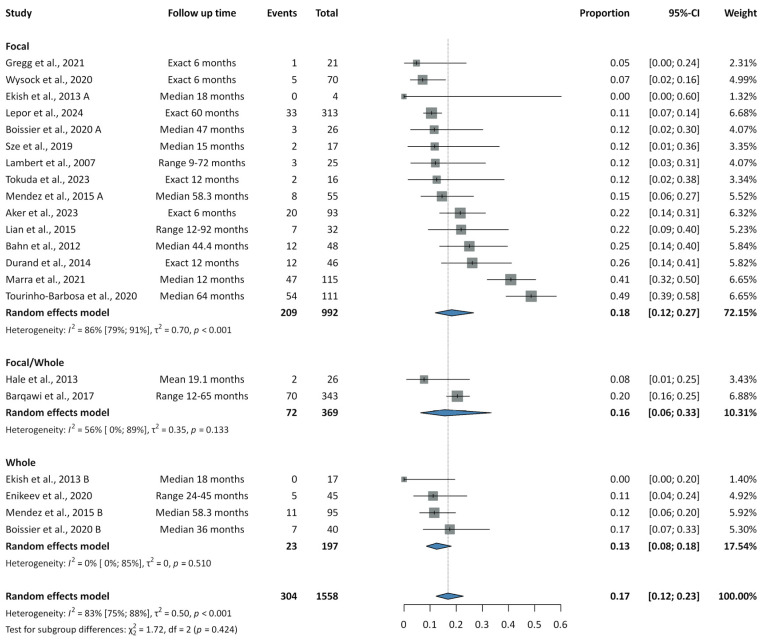
Summary forest plot of pooled recurrence rates for focal and whole-gland cryoablation [41,42,43,45,46,48,49,50,51,52,53,54,55,59,60,68,110,111].

**Figure 4:**
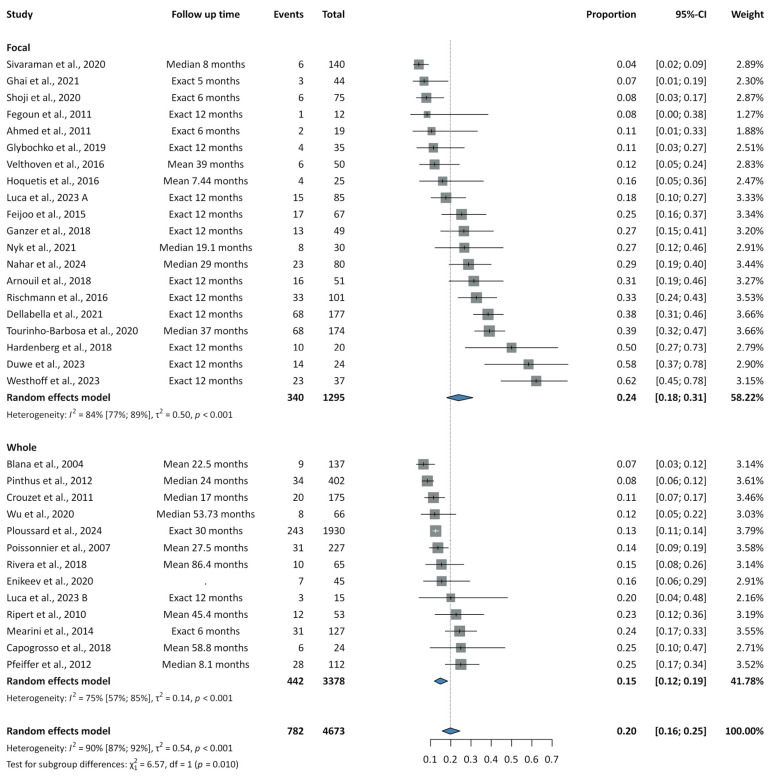
Summary forest plot of pooled recurrence rates for focal and whole-gland high-intensity focused ultrasound [69,70,71,75,76,77,78,79,80,81,82,83,84,85,86,87,88,89,90,91,94,95,96,103,104,105,107,108,110,111].

Results on recurrence rates are summarized in Figure 2, Figure 3 and Figure 4.

Forest plots illustrating recurrence rates following IRE are presented in Supplementary Figures S1–S3. In the extended subgroup at six months, two studies reported a recurrence rate of 0.26 (95% CI: 0.07–0.62; PI: NA; I^2^ = 93%). In the focal subgroup, eight studies yielded a six-month rate of 0.28 (95% CI: 0.19–0.40; PI: 0.07–0.66; I^2^ = 67%), and five studies reported a 12-month rate of 0.28 (95% CI: 0.15–0.46; PI: 0.03–0.85; I^2^ = 75%). The overall pooled recurrence rate across 14 studies was 0.29 (95% CI: 0.22–0.38; PI: 0.09–0.64; I^2^ = 74%). Subgroup analyses showed recurrence rates of 0.26 (95% CI: 0.07–0.62; PI: NA; I^2^ = 93%) for extended IRE and 0.30 (95% CI: 0.23–0.39; PI: 0.10–0.63; I^2^ = 68%) for focal IRE, with no significant difference between groups (*p* = 0.796). Among focal IRE studies, the pooled in-field recurrence rate was 0.14 (95% CI: 0.09–0.19; PI: 0.05–0.31; I^2^ = 36%), and the out-of-field recurrence rate was 0.15 (95% CI: 0.09–0.22; PI: 0.04–0.44; I^2^ = 57%). In- and out-of-field recurrence rates could not be assessed for extended IRE due to missing data.

Forest plots for cryoablation are presented in Supplementary Figures S4–S6. The pooled recurrence rate across 21 studies was 0.17 (95% CI: 0.12–0.23; PI: 0.04–0.48; I^2^ = 83%). When stratified by treatment strategy, recurrence rates were 0.18 (95% CI: 0.12–0.27; PI: 0.03–0.59; I^2^ = 86%) for focal and 0.13 (95% CI: 0.08–0.18; PI: 0.05–0.27; I^2^ = 0%) for whole-gland cryoablation, with no significant difference between groups (*p* = 0.424). In analyses combining both focal and whole-gland procedures, the pooled rates of in-field, out-field, and concurrent in- and out-field recurrence were 0.05 (95% CI: 0.04–0.07; PI: 0.03–0.08; I^2^ = 0%), 0.10 (95% CI: 0.06–0.15; PI: 0.02–0.32; I^2^ = 53%), and 0.02 (95% CI: 0.01–0.03; PI: 0.01–0.04; I^2^ = 0%), respectively.

Supplementary Figures S7–S10 present the forest plots for recurrence rates associated with HIFU. Considering the 12-month follow-up time, we were able to analyze only the focal HIFU group, yielding a recurrence rate of 0.33 (95% CI: 0.25–0.42; PI: 0.11–0.65; I^2^= 77%). Across 25 studies, the overall pooled rate was 0.20 (95% CI: 0.16–0.25; PI: 0.05–0.53; I^2^ = 90%). However, whole-gland HIFU demonstrated a statistically significant reduction in recurrence, with a rate of 0.15 (95% CI: 0.12–0.19; PI: 0.07–0.30; I^2^ = 75%), compared to 0.24 (95% CI: 0.18–0.31; PI: 0.06–0.59; I^2^ = 84%) for FT (*p* = 0.010). The pooled rates of in-field, out-field, and concurrent in- and out-field recurrence were 0.10 (95% CI: 0.06–0.16; PI: 0.02–0.43; I^2^ = 77%), 0.09 (95% CI: 0.05–0.14; PI: 0.02–0.35; I^2^ = 72%), and 0.02 (95% CI: 0.01–0.04; PI: 0.01–0.08; I^2^ = 17%), respectively.

### 3.4. Complications

Complication rates are summarized in Supplementary Figures S11–S16.

The overall complication rate associated with IRE was 0.37 (95% CI: 0.27–0.48; PI: 0.11–0.74; I^2^ = 82%). Subgroup analysis yielded rates of 0.43 (95% CI: 0.15–0.77; PI: NA; I^2^ = 94%) for extended IRE and 0.35 (95% CI: 0.25–0.47; PI: 0.10–0.73; I^2^ = 77%) for FT, with no statistically significant difference between groups (*p* = 0.683). Although the incidence of major complications remained low in both groups, extended IRE demonstrated a rate of 0.02 (95% CI: 0.01–0.08; PI: NA; I^2^ = 23%), while focal IRE showed a rate of 0.03 (95% CI: 0.02–0.06; PI: 0.02–0.07; I^2^ = 0%).

Cryoablation was associated with an overall complication rate of 0.21 (95% CI: 0.14–0.31; PI: 0.05–0.60; I^2^ = 71%). When stratified by treatment approach, FT presented a complication rate of 0.19 (95% CI: 0.13–0.27; PI: 0.07–0.43; I^2^ = 49%), while whole-gland cryoablation showed a higher rate of 0.41 (95% CI: 0.10–0.82; PI: 0.00–1.00; I^2^ = 78%), although the difference was not statistically significant (*p* = 0.334). Major complications remained low for both modalities, reported at 0.03 (95% CI: 0.01–0.04; PI: 0.01–0.05; I^2^ = 0%) for FT and 0.02 (95% CI: 0.01–0.06; PI: 0.00–0.32; I^2^ = 50%) for whole-gland ablation.

Regarding HIFU, the pooled complication rate was 0.29 (95% CI: 0.23–0.36; PI: 0.11–0.57; I^2^ = 88%), with subgroup rates of 0.28 (95% CI: 0.18–0.40; PI: 0.05–0.75; I^2^ = 87%) for focal and 0.30 (95% CI: 0.22–0.38; PI: 0.09–0.65; I^2^ = 92%) for whole-gland therapy. There was no statistically significant difference between the two approaches (*p* = 0.828). Major complications were similarly low for both, at 0.04 (95% CI: 0.03–0.07; PI: 0.01–0.15; I^2^ = 42%) for focal HIFU and 0.04 (95% CI: 0.02–0.10; PI: 0.00–0.46; I^2^ = 91%) for whole-gland HIFU.

### 3.5. Survival Outcomes: OS, CSS, and MFS

Survival outcomes, presented in Supplementary Figures S17–S25, were consistently favorable across all treatments, with no significant differences between focal and whole-gland subgroups.

For focal IRE, one-year OS was 98% (95% CI: 94–99; PI: 91–100; I^2^ = 0%), CSS was 98% (95% CI: 94–99; PI: 91–100; I^2^ = 0%), and MFS reached 99% (95% CI: 94–100; PI: 0–100; I^2^ = 0%).

Among patients treated with focal or whole-gland cryoablation, pooled OS, CSS, and MFS were 98% (95% CI: 95–99; PI: 69–100; I^2^ = 84%), 99% (95% CI: 99–100; PI: 99–100; I^2^ = 0%), and 99% (95% CI: 98–100; PI: 98–100; I^2^ = 0%).

Similarly, pooled OS, CSS, and MFS following focal and whole-gland HIFU were 97% (95% CI: 95–98; PI: 83–88; I^2^ = 76%), 99% (95% CI: 98–100; PI: 88–100; I^2^ = 59%), and 98% (95% CI: 96–99; PI: 74–100; I^2^ = 72%), respectively.

### 3.6. Biochemical Outcomes

Biochemical outcomes are shown in Supplementary Figures S26–S30.

During the follow-up periods, the mean postoperative PSA levels were 2.91 ng/mL (95% CI: 2.31–3.52; PI: 0.73–5.1; I^2^ = 86%) for IRE, 2.26 ng/mL (95% CI: 1.54–2.98; PI: 0.00–4.81; I^2^ = 93%) for cryoablation, and 2.39 ng/mL (95% CI: 1.83–2.96; PI: 0.00–4.85; I^2^ = 98%) for HIFU. Notably, a significant difference was observed between focal and whole-gland HIFU, with mean postoperative PSA levels of 2.81 ng/mL (95% CI: 2.41–3.21; PI: 1.28–4.34; I^2^ = 86%) and 0.68 ng/mL (95% CI: 0.35–1.01; PI: 0.00–4.74; I^2^ = 91%), respectively, in favor of the whole-gland technique (*p* < 0.001).

The one-year BRFS according to the Phoenix criteria for cryoablation in the treatment of pooled low-intermediate risk PCa was 95% (95% CI: 93–97; PI: 87–98; I^2^ = 49%), with a five-year BRFS of 81% (95% CI: 75–86; PI: 57–93; I^2^ = 91%). BRFS rates were 99% (95% CI: 93–100; PI: 43–100; I^2^ = 59%) and 86% (95% CI: 72–93; PI: 63–95; I^2^ = 87%) at one and five years in low-risk PCa, while 95% (95% CI: 88–98; PI: 68–99; I^2^ = 64%) and 78% (95% CI: 62–89; PI: 47–94; I^2^ = 92%) in intermediate-risk PCa patients. For HIFU, the one-year pooled BRFS was 96% (95% CI: 92–98; PI: 69–100; I^2^ = 78%), while the five-year pooled BRFS was 78% (95% CI: 72–84; PI: 53–92; I^2^ = 83%). In low-risk PCa, BRFS rates were 97% (95% CI: 88–99; PI: 65–100; I^2^ = 76%) at one year and 85% (95% CI: 77–90; PI: 59–96; I^2^ = 82%) at five years, whereas in intermediate-risk patients they were 96% (95% CI: 92–98; PI: 75–99; I^2^ = 74%) and 72% (95% CI: 59–82; PI: 38–92; I^2^ = 90%), respectively. Due to insufficient data, the analysis for IRE could not be performed.

### 3.7. Functional Outcomes

Functional outcomes are summarized in Supplementary Table S7.

The incidence of newly developed urinary incontinence ranged from 0 to 14% following focal IRE, while data for extended IRE were insufficient to analyze. For cryoablation, rates were 0–15% with FT and 0–23% with whole-gland ablation. No clinically relevant difference was observed between focal and whole-gland HIFU, with incidences of 0–20% and 0–22%, respectively.

De novo erectile dysfunction after focal and extended IRE was reported in 0–24% of cases. Focal cryoablation was associated with 0–31% rates, whereas whole-gland cryoablation showed higher rates of 0–53%. HIFU rates ranged from 0–33% with FT, compared to 12–53% with whole-gland treatment.

### 3.8. Retreatment

Retreatment forest plots are summarized in Supplementary Figures S31–S33.

In patients who underwent IRE, 7% (95% CI: 5–11; PI: 4–11; I^2^ = 0%) required a second IRE, 7% (95% CI: 4–13; PI: 2–27; I^2^ = 40%) received radical treatment, and 2% (95% CI: 1–5; PI: 1–5; I^2^ = 0%) were managed with hormonal therapy.

Following cryoablation, 7% (95% CI: 5–9; PI: 3–15; I^2^ = 40%) required a second cryoablation, 5% (95% CI: 2–9; PI: 1–32; I^2^ = 79%) underwent radical treatment, and 3% (95% CI: 1–6; PI: 0–28; I^2^ = 72%) were treated with hormonal therapy.

Among those who received HIFU, 6% (95% CI: 4–9; PI: 2–21; I^2^ = 75%) required a second HIFU procedure, 8% (95% CI: 6–12; PI: 2–30; I^2^ = 83%) progressed to radical treatment, and 3% (95% CI: 2–5; PI: 0–17; I^2^ = 72%) underwent hormonal therapy.

### 3.9. Risk of Bias Assessment

The results of the risk of bias assessment are presented in Supplementary Figures S34–S38. The overall risk was mostly moderate in retrospective and prospective studies, with 16 of them showing serious risk, especially for participant selection and missing data. Bias due to confounding was generally moderate, while bias related to intervention classification and deviations from intended interventions was typically low. In contrast, outcome measurement and reporting biases ranged from low to moderate. The only RCT included showed a low risk of bias across most domains, though some concerns were noted regarding missing outcome data for recurrence rates. The sensitivity analysis confirmed the robustness of our results, showing minimal sensitivity.

### 3.10. Publication Bias and Heterogeneity

We assessed publication bias visually using funnel plots and the trim-and-fill method, and statistically, we used Egger’s test. Our results all exceeded the 0.05 *p*-value threshold, indicating no significant publication bias.

Recurrence rates presented moderate to high heterogeneity, especially for focal IRE and HIFU. Complication rates were similarly variable, though major events remained low and consistent. Survival outcomes showed low heterogeneity overall, but HIFU-related survival rates were more variable, with moderate to high heterogeneity. Biochemical outcomes, such as PSA levels and BRFS, showed high heterogeneity, while retreatment rates were stable with low to moderate variability.

## 4. Discussion

We conducted a systematic review and meta-analysis to determine the effectiveness and safety profile of minimally invasive interventional therapies, such as IRE, cryoablation, and HIFU, for the treatment of low- and intermediate-risk PCa. Whole-gland HIFU showed a significantly lower recurrence rate than focal HIFU (15% vs. 24%, *p* = 0.010). In comparison, no statistically significant differences were found between focal and extended IRE (30% vs. 26%, *p* = 0.796) or between focal and whole-gland cryoablation (18% vs. 13%, *p* = 0.424). In-field and out-of-field recurrence rates were comparable within each treatment modality. Retreatment was generally low, with 6–7% of patients undergoing repeat ablation and 2–8% progressing to radical or hormonal therapy. Complication rates were higher with IRE (37%) and HIFU (29%) compared to cryoablation (21%), though major complications were consistently rare across all modalities. Survival outcomes were uniformly favorable regardless of treatment or approach, consistently exceeding 97%. Biochemical control was more pronounced with whole-gland HIFU, which resulted in significantly lower postoperative mean PSA levels than focal HIFU (0.68 vs. 2.81 ng/mL, *p* < 0.001). One-year BRFS exceeded 95% for both cryoablation and HIFU, while five-year BRFS approached 80%, with no comparable long-term data available for IRE.

Tay et al. analyzed FT outcomes—including IRE, cryoablation, and HIFU—across 49 cohorts, reporting favorable pooled survival rates: OS (98.0%), CSS (99.3%), and MFS (98.5%), with no significant differences between modalities, consistent with our findings. In contrast, they reported a biopsy-confirmed overall recurrence rate of 44.6%, with 22.2% classified as clinically significant cancer (8.9% in-field, 12.3% out-field), which differs from our results. Clinically significant PCa was not specifically emphasized in our analysis, as most included studies lacked sufficient detail on this outcome. Retreatment rates were comparable to our findings, with 5% of patients undergoing secondary FT and 10.5% progressing to radical treatment [112].

Nicoletti et al. highlighted the ongoing challenge of cancer recurrence following FT, with recurrence rates varying considerably by treatment modality and location. In-field recurrence ranged from 6–50% for HIFU, 0–56% for cryotherapy, and 0–33.3% for IRE. Nevertheless, OS and MFS remained consistently high (97–100%), aligning with our findings. For retreatment, HIFU required repeat ablation in 3.8–30% of cases and subsequent radical treatment in 2.2–25%, while cryotherapy retreatment rates ranged from 2.7–13% with 1.3–44% progressing to radical therapy, and IRE retreatment rates ranged from 1–10.5% [113].

Minimally invasive interventional procedures are currently considered investigational therapeutic approaches. Hopstaken et al. conducted a systematic review of FT, reporting a median recurrence rate of 15.4% in the treated area for HIFU, consistent with our findings, and a 2% complication rate. For IRE, the recurrence rate was 8.5%, while cryoablation showed a recurrence rate of 0–20% in the treated area, with grade 3 adverse events ranging from 0–9% [13]. In 2021, Shah et al. published a propensity score-matched study comparing FT (HIFU and cryoablation) with RP. Failure-free survival (FFS) rates at three, five, and eight years were similar between groups: 86%, 82%, and 79% for RP vs. 91%, 86%, and 83% for FT. Salvage therapy was required in 15.9% of RP patients and 24.4% of FT patients, with 17.1% requiring a second FT session [114]. In the literature evaluating functional outcomes after FT, the incidence of de novo urinary incontinence was comparable across the various treatment approaches, occurring in 0–14% of IRE patients, 0–15% of those undergoing cryoablation, and 0–20% of HIFU-treated cases [28,32,33,34,36,37,38,39,40,43,45,48,49,50,51,52,53,55,69,71,75,76,77,78,79,80,84,85,86,87,88]. Regarding sexual function, serious adverse effects, including erectile dysfunction, have been reported in approximately 6% of cases following FT, with no statistically significant differences between the treatment modalities [112].

Fewer studies have been published in the literature on whole-gland therapies. Bründl et al. analyzed 463 low- to intermediate-risk patients treated with whole-gland HIFU. After 15 years, CSS was 95% for low-risk and 89% for intermediate-risk patients, with 91% and 85% MFS rates. In contrast, the high-risk group showed significantly lower rates, with a CSS of 65% and MFS of 58% [115]. Tan et al.’s study on whole-gland cryoablation in 260 patients reported a 10-year FFS of 66% and MFS of 96%. Complications occurred in 8.8%, with 2.3% having grade ≥3 events, similar to our results. Both FFS and MFS were lower in high-risk patients [116]. Regarding functional outcomes, the incidence of treatment-related incontinence was slightly higher with whole-gland therapies, ranging from 0% to 23% for both cryoablation and HIFU [44,47,57,72,73,90,107,110]. Ganzer et al. reported a preserved potency rate of 25.4% among previously potent patients undergoing whole-gland HIFU [117]. Since whole-gland cryoablation yields comparable functional outcomes, most sexually active patients consequently prefer focal treatment strategies [116].

### 4.1. Strengths and Limitations

Regarding the strengths of our analysis, our results are based on a registered protocol and rigorous methodology, with a comprehensive review of clinically relevant outcomes across both focal and whole-gland minimally invasive therapies. In our analysis, we incorporated several recent studies, provided a more detailed and structured assessment of different retreatment strategies, and introduced various methodological improvements compared to previous reviews.

This work is limited by the predominance of retrospective and prospective single-arm studies, providing lower levels of evidence, insufficient data on preoperative variables, baseline differences in patient selection (e.g., tumor volume, multifocality, lesion location), physician preferences, operator skills, focus on predominantly short-term outcomes, a generally moderate risk of bias, and the inability to distinguish between low- and intermediate-risk cases. Furthermore, our analysis was not based on adjusted, direct head-to-head comparisons, and differences between treatment groups or subgroups may reflect variations in baseline characteristics or study design.

### 4.2. Implications for Practice and Research

The implementation of scientific knowledge is pivotal in providing benefits to the community [118,119]. Minimally invasive interventional therapies offer effective and safe treatment options for low- and intermediate-risk PCa, with favorable oncological, survival, and biochemical outcomes. Whole-gland HIFU may provide superior biochemical control and lower recurrence compared to focal HIFU, suggesting that treatment extent should be considered in clinical decision-making. Future research should focus on long-term outcomes, direct comparisons of these therapies, and refining patient selection criteria to optimize treatment efficacy and safety.

## 5. Conclusions

Minimally invasive focal and whole-gland therapies, including IRE, cryoablation, and HIFU, are effective and safe options for treating low- and intermediate-risk prostate cancer, with consistently high survival rates and low major complication rates. For IRE and cryoablation, recurrence rates and postoperative PSA levels are similar between focal and whole-gland approaches, while whole-gland HIFU offers superior biochemical control and lower recurrence than focal HIFU, highlighting the importance of treatment extent in clinical decision-making. However, these results need to be confirmed by further high-quality randomized trials.

## Data Availability

The original contributions presented in this study are included in the article/Supplementary Materials. Further inquiries can be directed to the corresponding author.

## Supplementary Materials

The following supporting information can be downloaded at: https://www.mdpi.com/article/10.3390/cancers17172863/s1, Table S1: PRISMA 2020 checklist; Table S2: The list of keywords for the systematic search; Table S3: Eligibility criteria of each included article; Tables S4–S6: Basic characteristics of included studies regarding irreversible electroporation (IRE)/cryoablation/high-intensity focused ultra-sound (HIFU); Table S7: Functional outcomes (newly developed urinary incontinence and sexual function); Figure S1: Summary forest plot of 6 and 12-month recurrence rates regarding focal and extended irreversible electroporation; Figure S2: Summary forest plot of pooled in-field recurrence rates regarding focal irreversible electroporation; Figure S3: Summary forest plot of pooled out-field recurrence rates regarding focal irreversible electroporation; Figure S4: Summary forest plot of pooled in-field recurrence rates regarding cryoablation; Figure S5: Summary forest plot of pooled out-field recurrence rates regarding cryoablation; Figure S6: Summary forest plot of pooled concurrent in- and out-field recurrence rates regarding cryo-ablation; Figure S7: Summary forest plot of 12-month recurrence rates regarding focal high-intensity focused ultrasound; Figure S8: Summary forest plot of pooled in-field recurrence rates regarding focal and whole gland high-intensity focused ultrasound; Figure S9: Summary forest plot of pooled out-field recurrence rates regarding focal and whole gland high-intensity focused ultrasound; Figure S10: Summary forest plot of pooled concurrent in- and out-field recurrence rates regarding focal and whole gland high-intensity focused ultrasound; Figure S11: Summary forest plot of total complication rates regarding focal and extended irreversible electroporation; Figure S12: Summary forest plot of major complication rates regarding focal and extended irreversible electroporation; Figure S13: Summary forest plot of total complication rates regarding focal and whole gland cryoablation; Figure S14: Summary forest plot of major complication rates regarding focal and whole gland cryoablation; Figure S15: Summary forest plot of total complication rates regarding focal and whole gland high-intensity focused ultrasound; Figure S16: Summary forest plot of major complication rates regarding focal and whole gland high-intensity focused ultrasound; Figure S17: Summary forest plot of 12-month overall survival rates re-garding focal irreversible electroporation; Figure S18: Summary forest plot of 12-month can-cer-specific survival rates regarding focal irreversible electroporation; Figure S19: Summary forest plot of 12-month metastasis-free survival rates regarding focal irreversible electro-poration; Figure S20: Summary forest plot of pooled overall survival rates regarding focal and whole gland cryoablation; Figure S21: Summary forest plot of pooled cancer-specific survival rates regarding focal and whole gland cryoablation; Figure S22: Summary forest plot of pooled metastasis-free survival rates regarding focal and whole gland cryoablation; Figure S23: Summary forest plot of pooled overall survival rates regarding focal and whole gland high-intensity focused ultrasound; Figure S24: Summary forest plot of pooled cancer-specific survival rates regarding focal and whole gland high-intensity focused ultrasound; Figure S25: Summary forest plot of pooled metastasis-free survival rates regarding focal and whole gland high-intensity focused ultrasound; Figure S26: Summary forest plot of mean postoperative PSA levels regarding focal and extended irreversible electroporation; Figure S27: Summary forest plot of mean postoperative PSA levels regarding focal and whole gland cryoablation; Figure S28: Summary forest plot of mean postoperative PSA levels regarding focal and whole gland high-intensity focused ultrasound; Figure S29: Summary forest plot of 12- and 60-month BFRS regarding focal and whole gland cryoablation (Phoenix criteria); Figure S30: Summary forest plot of 12- and 60-month BFRS regarding focal and whole gland high-intensity focused ultra-sound (Phoenix criteria); Figures S31: Summary forest plot of retreatments following focal ir-reversible electroporation: A, second IRE; B, radical treatment; and C, hormonal therapy; Figures S32: Summary forest plot of retreatments following focal and whole gland cryoablation: A, second cryoablation; B, radical treatment; and C, hormonal therapy; Figures S33: Summary forest plot of retreatments following focal and whole gland high-intensity focused ultrasound: A, second HIFU; B, radical treatment; and C, hormonal therapy; Figure S34: Risk of bias as-sessment using the ROBINS-I tool Regarding IRE; Figure S35: Risk of bias assessment using the ROBINS-I tool Regarding cryoablation; Figure S36: Risk of bias assessment using the ROBINS-I tool Regarding HIFU; Figure S37: Risk of bias assessment using the RoB2 tool Regarding com-plication rates; Figure S38: Risk of bias assessment using the RoB2 tool Regarding recurrence rates.

## Abbreviations

AS	Active surveillance
BFRS	Biochemical recurrence-free survival
CI	Confidence interval
CSS	Cancer-specific survival
FFS	Failure-free survival
FT	Focal therapy
HIFU	High-intensity focused ultrasound
IRE	Irreversible electroporation
ISUP	International Society of Urological Pathology
MFS	Metastasis-free survival
MRI	Magnetic resonance imaging
NA	Not available
NCCN	National Comprehensive Cancer Network
OR	Odds ratio
OS	Overall survival
PCa	Prostate cancer
PICO	Population, Intervention, Comparison, Outcome
PI	Prediction interval
PRISMA	Preferred Reporting Items for Systematic Reviews and Meta-Analyses
PSA	Prostate-specific antigen
RCT	Randomized controlled trials
ROBINS-I	Risk of Bias In Non-randomized Studies of Interventions
RoB 2	Risk of Bias 2
RP	Radical prostatectomy
RT	Radiotherapy
TNM	Tumor Node Metastasis

## Appendix A. Risk of Bias Assessment Methodology Regarding Prospective and Retrospective Non-Randomized Cohort and Registry Studies (ROBINS-I Tool)

**Bias Due to Confounding**: (1) Low risk of bias: Assigned when no confounding factors were anticipated. (2) Moderate risk of bias: Assigned when confounding was expected, but all essential domains (e.g., age, preoperative PSA levels, Gleason score, clinical stage, NCCN risk category) were appropriately measured and controlled for. The reliability and validity of measurements were sufficient, so residual serious confounding was unlikely. (3) Serious risk of bias: Assigned when at least one crucial domain was either not correctly measured or not controlled for or if the reliability or validity of the measurements was inadequate, suggesting the presence of serious residual confounding. (4) Critical risk of bias: Assigned when confounding was inherently uncontrollable or negative controls strongly indicated the presence of unmeasured confounding. (5) No information: Assigned when there was insufficient information to determine whether confounding might exist.

**Bias in Selection of Participants for the Study**: (1) Low risk of bias was assigned when participants were enrolled in a clearly defined manner, with inclusion and exclusion criteria transparently applied. (2) Moderate risk of bias was assigned when selection was appropriately managed but with minor concerns, such as vague reporting on how criteria were enforced, although unlikely to impact study outcomes. (3) Serious risk of bias was assigned when selection criteria were not clearly defined or if there was evidence suggesting systematic differences in how participants were included, raising concerns about selection bias. (4) Critical risk of bias was assigned when selection appeared arbitrary or heavily dependent on factors likely to introduce systematic differences in the outcome. (5) No information was assigned when the study failed to report sufficient details on participant selection.

**Bias in Classification of Interventions**: (1) Low risk of bias was assigned when interventions were clearly defined, with no misclassification issues apparent. (2) Moderate risk of bias was assigned when minor uncertainties regarding intervention classification were present but unlikely to affect the outcomes significantly. (3) A serious risk of bias was assigned when intervention classification was unclear or inconsistent, potentially introducing systematic errors into the analysis. (4) Critical risk of bias was assigned when there was substantial misclassification of interventions that would severely undermine the integrity of the study results. (5) No information was assigned when the study did not provide enough details on how interventions were classified.

**Bias Due to Deviations from Intended Interventions**: (1) Low risk of bias was assigned when deviations from intended interventions were minimal or not likely to influence the study outcomes. (2) A moderate risk of bias was assigned when there were some deviations from the protocol, but these deviations were unlikely to affect the study results significantly. (3) Serious risk of bias was assigned when deviations occurred that could plausibly influence outcomes, especially when it was unclear whether the deviations were related to the intended intervention. (4) Critical risk of bias was assigned when deviations from the intended intervention were substantial and likely to bias the results. (5) No information was assigned when there was inadequate detail to determine if deviations from the intervention occurred.

**Bias Due to Missing Data**: (1) Low risk of bias was assigned when missing data were minimal and were unlikely to influence the results. (2) Moderate risk of bias was assigned when missing data were present but were appropriately handled using acceptable imputation methods or when missingness was unlikely to be associated with the outcome. (3) Serious risk of bias was assigned when a substantial proportion of data was missing or if the methods for handling missing data were inadequate or unclear, increasing the risk of bias. (4) Critical risk of bias was assigned when the amount or nature of missing data was substantial enough to severely undermine the validity of the findings. (5) No information was assigned when missing data were not reported, and the extent of missingness could not be determined.

**Bias in Measurement of Outcomes**: (1) Low risk of bias was assigned when outcome measurement was clearly defined, with valid and reliable tools used consistently across participants. (2) Moderate risk of bias was assigned when minor concerns arose about measurement accuracy or consistency, but these were unlikely to affect the study conclusions. (3) Serious risk of bias was assigned when outcome measurement was inadequately reported or inconsistently applied, introducing a significant potential for bias. (4) Critical risk of bias was assigned when measurement methods were so flawed or inconsistent that they would likely compromise the validity of the findings. (5) No information was assigned when the study failed to provide sufficient details about measuring outcomes.

**Bias in Selection of the Reported Result**: (1) Low risk of bias was assigned when all prespecified outcomes were reported, and no evidence suggested selective reporting. (2) A moderate risk of bias was assigned when there was some concern about selective reporting, but it was unlikely to affect the study’s main conclusions. (3) A serious risk of bias was assigned when important outcomes appeared to be selectively reported, potentially biasing the overall findings. (4) Critical risk of bias was assigned when selective reporting was so substantial that the study’s conclusions were highly suspect. (5) No information was assigned when the study did not provide enough detail to determine if selective reporting occurred.

## Appendix B. Risk of Bias Assessment Methodology Regarding Randomized Controlled Trials of Interventions (RoB2 Tool)

**Randomization process**: (1) A low risk of bias is indicated when the randomization is adequately generated and concealed, ensuring that participants have an equal chance of being assigned to any intervention group. (2) Concerns arise when the randomization method is unclear or inadequately described, leading to uncertainty about the comparability of groups. (3) A high risk of bias is present when evidence of non-random assignment, such as systematic differences between groups at baseline, could influence outcomes.

**Deviations from intended interventions**: (1) A low risk of bias is found when participants receive the intended interventions as per the protocol, with minimal deviations. (2) Some concerns may exist if there are notable deviations, such as participant crossover between groups or differences in intervention implementation, but these do not substantially affect the outcomes. (3) A high risk of bias occurs when there are significant deviations from the intended interventions that likely impact the effect of the treatment being evaluated.

**Missing outcome data**: (1) A low risk of bias is identified when there is a low level of missing data, and the reasons for any missingness are unlikely to be related to the outcomes. (2) Some concerns are raised when there is a moderate amount of missing data, especially if the reasons are unclear, which may introduce bias. (3) A high risk of bias is evident when there is a substantial amount of missing data, particularly if it is related to the outcomes and could significantly affect the study’s conclusions.

**Measurement of the outcome**: (1) A low risk of bias is indicated when outcomes are measured using valid and reliable methods, with assessors blinded to group assignment. (2) Some concerns arise when the measurement methods are not well described or if there is a lack of blinding, which could influence the outcomes. (3) There is a high risk of bias when there is clear evidence that the outcome measurement is flawed or biased, such as using subjective assessments without appropriate validation.

**Selection of the reported result**: (1) A low risk of bias is characterized by a pre-defined analysis plan that aligns with the reported outcomes, ensuring transparency and reducing selective reporting. (2) Some concerns may arise if there is some discrepancy between the planned and reported outcomes, suggesting potential selective reporting, but it does not substantially alter the conclusions. (3) A high risk of bias is indicated when there is clear evidence of selective reporting, where only favorable outcomes are reported or analyzed, potentially misleading the interpretation of the findings.
