# Supplementary material for: Oncological Efficacy and Safety of Minimally Invasive Focal and Whole-Gland Interventions in the Treatment of Low- and Intermediate-Risk Prostate Cancer: A Systematic Review and Meta-Analysis
Source: Cancers (Basel). 2025 Aug 30;17(17):2863. doi: 10.3390/cancers17172863 (PMC12427472; doi:10.3390/cancers17172863)
Supplement: Supplementary file 1 [file cancers-17-02863-s001.zip › cancers-3808318-supplementary.pdf]

cancers

MDPI

Supplementary Materials

Oncological Efficacy and Safety of Minimally Invasive Focal and Whole-Gland Interventions in the Treatment of Low- and Intermediate-Risk Prostate Cancer: A Systematic Review and Meta-Analysis

Benjamin Skribek, Anett Szabó, Júlia Ács, Bianca Golzio Navarro Cavalcante, Boglárka Dorina Sipos, Péter Hegyi , Péter Mátrai , Péter Nyirády, Nándor Ács, Attila Majoros and Pál Ákos Deák

Table S1. PRISMA 2020 checklist.

Section and topic	Item #	Checklist item	Location where item is reported
		**Title**	
Title	1	Identify the report as a systematic review.	1
		**Abstract**	
Abstract	2	See the PRISMA 2020 for Abstracts checklist (table 2).	1-2, Suppl. Table S1
		**Introduction**	
Rationale	3	Describe the rationale for the review in the context of existing knowledge.	2
Objectives	4	Provide an explicit statement of the objective(s) or question(s) the review addresses.	2
		**Methods**	
Eligibility criteria	5	Specify the inclusion and exclusion criteria for the review and how studies were grouped for the syntheses.	3
Information sources	6	Specify all databases, registers, websites, organizations, reference lists and other sources searched or consulted to identify studies. Specify the date when each source was last searched or consulted.	3
Search strategy	7	Present the full search strategies for all databases, registers, and websites, including any filters and limits used.	3-4 Figure 1 Suppl. Table S2
Selection process	8	Specify the methods used to decide whether a study met the inclusion criteria of the review, including how many reviewers screened each record and each report retrieved, whether they worked independently, and if applicable, details of automation tools used in the process.	3-4
Data collection process	9	Specify the methods used to collect data from reports, including how many reviewers collected data from each report, whether they worked independently, any processes for obtaining or confirming data from study investigators, and if applicable, details of automation tools used in the process.	3-4
Data items	10a	List and define all outcomes for which data were sought. Specify whether all results that were compatible with each outcome domain in each study were sought (e.g., for all measures, time points, analyses), and if not, the methods used to decide which results to collect.	3-4

 https://doi.org/10.3390/cancers17172863

	10b	List and define all other variables for which data were sought (e.g., participant and intervention characteristics, funding sources). Describe any assumptions made about any missing or unclear information.	3-4
Study risk of bias assessment	11	Specify the methods used to assess risk of bias in the included studies, including details of the tool(s) used, how many reviewers assessed each study and whether they worked independently, and if applicable, details of automation tools used in the process.	4
Effect measures	12	Specify for each outcome the effect measure(s) (e.g., risk ratio, mean difference) used in the synthesis or presentation of results.	5
Synthesis methods	13a	Describe the processes used to decide which studies were eligible for each synthesis (e.g., tabulating the study intervention characteristics and comparing against the planned groups for each synthesis (item #5)).	4-5
	13b	Describe any methods required to prepare the data for presentation or synthesis, such as handling of missing summary statistics, or data conversions.	4-5
	13c	Describe any methods used to tabulate or visually display results of individual studies and syntheses.	4-5
	13d	Describe any methods used to synthesize results and provide a rationale for the choice(s). If meta-analysis was performed, describe the model(s), method(s) to identify the presence and extent of statistical heterogeneity, and software package(s) used.	4-5
	13e	Describe any methods used to explore possible causes of heterogeneity among study results (e.g. subgroup analysis, meta-regression).	4-5
	13f	Describe any sensitivity analyses conducted to assess robustness of the synthesized results.	4-5
Reporting bias assessment	14	Describe any methods used to assess risk of bias due to missing results in a synthesis (arising from reporting biases).	4<br>Suppl. appendix A and B
Certainty assessment	15	Describe any methods used to assess certainty (or confidence) in the body of evidence for an outcome.	5
		**Results**	
Study selection	16a	Describe the results of the search and selection process, from the number of records identified in the search to the number of studies included in the review, ideally using a flow diagram (see fig 1).	5
	16b	Cite studies that might appear to meet the inclusion criteria, but which were excluded, and explain why they were excluded.	5, Figure 1<br>Suppl. material
Study characteristics	17	Cite each included study and present its characteristics.	5<br>Suppl. Table S4-S6
Risk of bias in studies	18	Present assessments of risk of bias for each included study.	10<br>Suppl. Appendices A and B
Results of individual studies	19	For all outcomes, present, for each study: (a) summary statistics for each group (where appropriate) and (b) an effect estimate and its precision (e.g. confidence/credible interval), ideally using structured tables or plots.	5-10<br>Figures 2-4.<br>Suppl. Figures S1-S33. and Suppl. Table S7.

Results of syntheses	20a	For each synthesis, briefly summarize the characteristics and risk of bias among contributing studies.	5-10 Suppl. Figures S34-S38.
	20b	Present results of all statistical syntheses conducted. If meta-analysis was done, present for each the summary estimate and its precision (e.g. confidence/credible interval) and measures of statistical heterogeneity. If comparing groups, describe the direction of the effect.	5-10 Figures 2-4. Suppl. Figures S1-S33.
	20c	Present results of all investigations of possible causes of heterogeneity among study results.	10 Figures 2-4. Suppl. Figures S1-S33.
	20d	Present results of all sensitivity analyses conducted to assess the robustness of the synthesized results.	10
Reporting biases	21	Present assessments of risk of bias due to missing results (arising from reporting biases) for each synthesis assessed.	10
Certainty of evidence	22	Present assessments of certainty (or confidence) in the body of evidence for each outcome assessed.	5-10 Figures 2-4. Suppl. Figures S1-S33.
**Discussion**			
Discussion	23a	Provide a general interpretation of the results in the context of other evidence.	11-12
	23b	Discuss any limitations of the evidence included in the review.	12
	23c	Discuss any limitations of the review processes used.	12
	23d	Discuss implications of the results for practice, policy, and future research.	12
**Other information**			
Registration and protocol	24a	Provide registration information for the review, including register name and registration number, or state that the review was not registered.	3
	24b	Indicate where the review protocol can be accessed, or state that a protocol was not prepared.	3
	24c	Describe and explain any amendments to information provided at registration or in the protocol.	3
Support	25	Describe sources of financial or non-financial support for the review, and the role of the funders or sponsors in the review.	14
Competing interests	26	Declare any competing interests of review authors.	14
Availability of data, code, and other materials	27	Report which of the following are publicly available and where they can be found: template data collection forms; data extracted from included studies; data used for all analyses; analytic code; any other materials used in the review.	14

Table S2. The list of keywords for the systematic search.

Databases	Search Key
Pubmed<br>EMBASE<br>Cochrane Library	(prostat*) **AND** (adenoma OR tumor OR tumour OR cancer OR neoplasm OR neoplasia OR carcinoma) **AND** (non-invasive OR minimally invasive OR noninvasive OR high-intensity focused ultrasound OR high intensity focused ultrasound OR HIFU OR IRE OR electroporation OR thermo* OR radiofrequency OR RFA OR microwave OR MWA OR ablat* OR cryoab* OR cryother* OR embolization OR embolisation) **AND** (local* OR primar* OR low-risk OR intermediate-risk OR low risk OR intermediate risk)

*We used the same keywords in each database.

Table S3. Eligibility criteria of each included article.

Author (year)	Inclusion criteria	Exclusion criteria
Rosette et al., 2023 [1]	Clinical stage T1c-T2b, Gleason sum score 6 or 7 (without tumor volume threshold), PSA <15 ng/mL or PSA >15ng/mL counseled with caution, Life expectancy of >10 years	Bleeding disorder as determined by prothrombin time >14.5 seconds, partial thromboplastin time >34 seconds, and platelet count <140/mL.. No ability to stop anticoagulant or anti-platelet therapy for 7 days prior to the procedure, Active urinary tract infection, History of bladder neck contracture, American Society of Anesthesiologists category IV or greater, History of inflammatory bowel disease, Concurrent major debilitating illness, Prior or concurrent malignancy except for basal cell carcinoma of the skin, Cardiac history including arrhythmias, implant able cardioverter defibrillator, or pacemaker, Prostate calcifications greater than 5 mm, Biological or chemotherapy for prostate cancer, Hormonal therapy for prostate cancer within 6 months prior to procedure, Previous radiation to pelvis, Transurethral resection of the prostate/urethral stent, Prior major rectal surgery (except hemorrhoids)
Collettini et al., 2019 [2]	Eligible participants were men (age >18 years) with biopsy-proven, treatment-naive, low-to-intermediate-risk nonmetastatic prostate cancer (PSA level, ≤ 15 ng/mL; Gleason score, ≤ 3 + 4; clinical stage, ≤ T2c; lesion size at multiparametric prostate MRI, ≤ 20 mm).	Participants were excluded if they had undergone previous radiation therapy for prostate cancer, previous or concomitant androgen suppression therapy, or previous focal therapy of the prostate.
Wang et al., 2022 [3]	Patients with low- or intermediate-risk PCa were eligible as defined by the National Comprehensive Cancer Risk classification. PCa was verified by template-guided mapping biopsy or targeted plus template-guided mapping biopsy. Other inclusion criteria included the following: participants aged 40 to 85 years, serum prostate-specific antigen (PSA) level less than 20 ng/mL (to convert to micrograms per liter, multiplied by 1), clinical stage of T2c or less (defined based on digital rectal examination); and Gleason score of 7 or less.	The exclusion criteria included the following: (1) prior radical prostatectomy, hormonal therapy, or radiotherapy; (2) prostatic calculus greater than 5 mm; (3) history of epilepsy (4) cardiac pacemaker or any metal implant between L1 and midfemur level; and (5) any other malignant tumor.
Shin et al., 2023 [4]	Not defined	Not defined

Giganti et al., 2019 [5]	(1) biopsy-proven tumor; (2) serial mpMRI scans before and after the procedure, as follows: pre-treatment, early (within 10 days from the procedure, to evaluate the effect of the treatment, including any evidence of rectourethral fistula) and at 6 months.	Not defined
Blazevski et al., 2019 [6]	Low (high-volume > 4 mm)- to intermediate risk PCa (D'Amico), Gleason score ≤ 7 (ISUP ≤ 3), Unilateral or midline anterior/posterior index tumor, allowing single targeted ablative therapy, PSA ≤ 15 ng/ml, Life expectancy ≥ 10 years, No previous treatment for PCa, No previous androgen suppression treatment for PCa, Minimum 12-month follow-up, Multiple lesions which can be encompassed in one treatment	Bilateral significant disease, Metastatic disease, Multiple lesions that cannot be treated within one treatment field
Popeneciu et al., 2024 [7]	(1) Histopathologic diagnosis of PCa- Maximum Gleason score (GS): 3+4=7a- Prostate-specific antigen (PSA) ≤20 ng/mL- Unifocal unilateral appearance (histology) and ≤15 mm in greatest diameter (multiparametric MRI, mpMRI)-Carcinoma lesions positively detected by mpMRI/transrectal ultrasonography (TRUS) fusion biopsy of the prostate (PIRADS Score >2) using a targeted and systematic biopsy protocol-mpMRI (PI-RADS ver. 2.0): no involvement of the prostate capsule or extraprostatic extension-mpMRI minimum safety distance from positive lesion to rectal wall of 10 mm and urethral safety distance of 6 mm (2) Karnofsky performance status scale: >80% (3) No previous history of prostate malignancy or treatment (4) Patient refusal of AS treatment and other standard treatment options (5) Life expectancy >10 years (clinician's expert opinion)	Any other histological patterns of PCa, anesthesiologic reasons (pacemaker/defibrillator implants, contraindications for i.v. anesthesia or relaxant medication), clinical appearance (Karnofsky performance status scale, comorbidities), or patient's preference for any other established curative treatment or AS led to exclusion from the study.
Yaxley et al., 2022 [8]	Patients with localized PCa based on staging prostate-specific membrane antigen positron emission tomography/computed tomography (PSMA PET/CT) scan.	All patients required a pre-operative multiparametric magnetic resonance imaging (mpMRI), with radiological evidence of extraprostatic extension or seminal vesicle invasion (stage T3a/T3b).
Altan et al., 2023 [9]	Patients who had first-year confirmation biopsies were defined as low- and intermediate-risk patients with localized prostate cancer according to the guidelines of the European Urology Association.	Patients in the high-risk group and those who had not yet undergone their first-year confirmation biopsies.
Murray et al., 2016 [10]	Patients with a diagnosis of prostate cancer who were counseled and offered conventional management options (surveillance, surgery, radiation) but did not accept and requested IRE were considered for this approach. Patients in the primary IRE cohort included men with Gleason grade	Not defined

Ting et al., 2015 [11]	Signed informed consent by the patient (Men who either refuse or feel unsuitable for whole-gland therapy or men who have been counseled regarding IRE for prostate cancer and its relatively experimental nature), Age ⩾40 years, Visible lesion on mp-MRI with no evidence of ECE or SVI, Stage ⩽T2c on mp-MRI, Transperineal, TRUS or MRI-guided biopsies correlating with the visible lesion on mp-MRI, Gleason score ⩽7 on biopsy, Low-intermediate risk disease (D'Amico Criteria)	Men who are unable to give informed consent, Men who are unable to undergo MRI (for example, incompatible metallic implant, claustrophobia), Men with evidence of metastatic or nodal disease outside the prostate on mp-MRI or other imaging, Men who have had previous treatment for their prostate cancer
Valerio et al., 2016 [12]	Histologically proven prostate cancer, overall Gleason Score ≤ 4 + 3, Anterior visible lesion on mpMRI accessible to irreversible electroporation ablation, Transperineal prostate biopsies (template mapping and/or limited and targeted) concordant with the MR-visible lesion, Absence of clinically significant disease outside planned treatment area, Radiological stage T1-T3a N0 M0, Serum PSA ≤ 15 ng/ml, Age ≥ 40 years and life expectancy of ≥ 10 years, Signed informed consent, Knowledge of the English language sufficient to understand written and verbal information about the trial and consent process	Previous radiation therapy to the pelvis, Previous prostate ablation (HIFU, cryotherapy, electroporation, microwave, etc.). Transurethral Resection of the Prostate (TURP) for symptomatic lower urinary tract symptoms within the prior 6 months. These patients may be included within the trial if deferred from consent and screening until at least 6 months following the TURP, Androgen suppression/hormone treatment within the previous 12 months, Evidence of metastatic disease or nodal disease outside the prostate on bone scan or cross-sectional imaging, Unable to have pelvic MRI scanning (severe claustrophobia, permanent cardiac pacemaker, metallic implant etc. likely to contribute significant artifact to images), Presence of metal implants/stents in the urethra, Renal impairment with a GFR of <35ml/min (unable to tolerate Gadolinium dynamic contrast-enhanced MRI), Non-visible tumor on mpMRI - Inability to tolerate a transrectal ultrasound or prior significant rectal surgery preventing insertion of the TRUS probe (decided on the type of surgery in individual cases), Latex allergies, Unfit for surgery
Bos et al., 2017 [13]	Low- to intermediate-risk PCa (D'Amico), Gleason score ≤7 (ISUP Grade ≤3), Unilateral or single midline anterior/posterior index tumor, allowing single targeted ablative therapy, Life expectancy ≥10 years, No previous treatment for PCa, No previous androgen suppression/hormone treatment for PCa	Not defined

López et al., 2023 [14]	1. Male subjects of 18 years of age and above. 2. Histologically proven PCa, Gleason ≤ 7 (3+4). 3. Tumor visible on mpMRI (PI-RAD ≥ 3/5) confirmed histologically by transperineal targeted biopsy. Positive cores concordant with visible lesion on mpMRI. 4. Intraprostatic tumor location should allow treatment without compromising neurovascular bundles or bladder neck, except for those patients with erectile dysfunction. 5. Absence of tumor in normal areas on mpMRI (systematic biopsy). 6. Absence of tumor-wide capsular contact on mpMRI except for anterior tumors. 7. Clinical stage T1-T2bN0M0. 8. PSA ≤ 15 ng/ml. 9. Life expectancy ≥ 10 years. 10. Capable of giving written informed consent, which includes compliance with the requirements and restrictions listed in the consent form. 11. Informed consent signed by the patient.	1. Men with any previous prostate treatment for PCa. 2. Men who have been on hormone therapy for PCa 12 months prior to treatment. 3. Men with evidence of metastatic disease. 4. Men who cannot tolerate transrectal ultrasound. 5. Men with latex allergy (transrectal probe is covered with a latex condom). 6. Men with previous significant transrectal surgery that prevents the passage of the transrectal probe. 7. Men who are unable to undergo general anesthesia.
Tokuda et al., 2023 [15]	Not defined	Not defined
Wysock et al., 2020 [16]	a single reported mpMRI region of interest concordant with biopsy Gleason grade < 4, no gross extraprostatic extension on mpMRI, no Gleason grade>1 or Gleason grade 1 core length>5mm contralateral to the region of interest-to-treat on systematic biopsy	Not defined
Sze et al., 2019 [17]	Not defined	Not defined
Kim et al., 2012 [18]	Not defined	Not defined
Bahn et al., 2012 [19]	biopsy-proven, clinically unilateral, low-intermediate risk (PSA ≤ 20, Gleason score ≤ 7, clinical stage T1-T2b) prostate cancer	(1) clinically bilateral cancer, (2) Gleason score 8, and (3) biopsy-proven extraprostatic extension of cancer
Barqawi et al., 2017 [20]	T1c, PSAD < 0.15 ng/mL, Gleason 6 or less, two or fewer positive biopsy cores, max 50% involvement of any core	Not defined
Hayek et al., 2008 [21]	Not defined	Not defined
Gregg et al., 2021 [22]	Serum PSA ≤ 10 ng/mL and diagnostic prostate biopsy revealing prostate adenocarcinoma with: (1) < 50% of cores from one side of the prostate positive for adenocarcinoma, (2) No greater than 50% tumor core length in any positive core, (3) Gleason grade group (GG) 1 or 2 disease, (4) No GG 4 or 5 tertiary pattern. Patients with contralateral disease were eligible only when the contralateral disease was GG1 and ≤ 2 mm in a single core.	Men were excluded who had medical contraindications to treatment, including inability to hold antiplatelet or anticoagulative medications, those that had an AUA symptom score > 19, or had signs and symptoms of a urinary tract infection.
Lambert et al., 2007 [23]	The patients were eligible for focal TCAP if they had Gleason score 6 or Gleason score 7 (3 4) prostate cancer confined to one lobe in one or two contiguous biopsy cores and a tumor volume of less than 10% in a 12-core	Not defined

	biopsy. These patients had not undergone hormonal therapy or radiotherapy.	
Lian et al., 2015 [24]	No previous PCa-related treatment, PSA (ng/ml) <20, Biopsy cores (n) ≥12, Positive cores (n) 1–2, Core involvement of a single core, % <50, Gleason score 6–7, Gland involvement: Unilateral, Clinical stage T1c to T2b	Not defined
Mendez et al., 2015 [25]	Low-risk criteria according to the D'Amico definition, that is, biopsy Gleason score ≤ 6, PSA level at diagnosis <10ng/mL, and clinical stage ≤ T2a. Furthermore, included only men with preoperative erectile function sufficient for penetration during sexual intercourse.	Patients underwent whole gland ablation using a ''nerve-warming'' technique whereby a probe was placed adjacent to the nerve(s) to try to maintain a warm temperature during the cryoablation process.
Marra et al., 2021 [26]	Institutional protocol generally comprised preoperative 1.5 or 3.0 T multiparametric magnetic resonance imaging (mpMRI) with FC being performed for low-/intermediate-risk PCa (defined as prostate-specific antigen (PSA) <20 ng/ml, ISUP ≤ 2, and clinical stage ≤T2c). A bone scan and cross-sectional abdominal imaging (mpMRI and/ or computed tomography (CT) scan) were performed to rule out extraprostatic disease in intermediate-risk PCa. Biopsies were performed using a systematic (at least ten cores), saturation, or mpMRI targeted (at least ten systematic plus three targeted cores) approach depending on the period of inclusion, mpMRI findings, treating physician, and clinical case.	cT3 disease
Barret et al., 2013 [27]	Low-risk PCa according to the D'Amico criteria (PSA <10ng/ml, Gleason sum 6, clinical stage T2a or less), unilateral disease, and fewer than three positive biopsies.	Clinically bilateral cancer, Gleason score 7, extracapsular extension proven on biopsy or suspected on multiparametric magnetic resonance imaging (MRI), and have received androgen deprivation therapy by referring physicians.
Enikeev et al., 2020 [28]	Low-risk prostate cancer according to the D'Amico classification (Gleason score 3 + 3 = 6; PSA <10 ng/ml; T1-T2a), two or fewer positive cores in one lobe, and a prostate volume of ≤50 cc.	Any prior treatment of the prostate, urethral stricture, bladder neck sclerosis, or LUTS (IPSS <15).
Durand et al., 2014 [29]	No previous PCa-related treatment; Clinical stage: T1c to T2a; PSA <10 ng/mL; Number of biopsy cores ≥12; Positive cores (%) <33; Core involvement of a single core (%): <50; Gland involvement: Unilateral; Gleason score ≤6; No extraprostatic extension on multiparametric MRI	Not defined
Hale et al., 2013 [30]	PSA<10ng/mL, PSAD<0.15ng/mL/g, Clinical stage T1NxMx or T2aNxMx, Gleason score: 3+3 or less, No more than 2 adjacent regions positive for	Not defined

	cancer, Total length of cancer < 10 mm total and <7mm in any 1 core; <1/3 of cores positive for cancer	
Boissier et al., 2020 [31]	Patients with ≥70 years old or younger if unfit for radical prostatectomy or radiotherapy, low-intermediate D'Amico risk PCa, estimated prostate volume of ≤60 gr.	Indication of cryotherapy for salvage treatment, follow-up of less than one-year, high-risk PCa (≥ T2c and/or PSA >20 ng/mL and/or Gleason ≥ 8), and metastatic patients.
Rodríguez et al., 2014 [32]	Inclusion criteria comprised patients diagnosed with localized prostate cancer at clinical stage T1c-T2c, and negative extension studies were carried out, whether the value of PSA at diagnosis was above 20ng/mL, with the exception of two patients treated in clinical stage T3aN0M0 as described later.	Not defined
Elkjær et al., 2014 [33]	Not defined	Not defined
Guo et al., 2020 [34]	Clinical T1c T2b tumor, prostate specific antigen (PSA) ≤20ng/ml before treatment, and biopsy GS ≤7 (3 + 3, 3 + 4, and 4 + 3).	Patients who had not received RP or CA or had incomplete clinicopathological data were primarily excluded. Then, patients with high-risk disease, radiotherapy experience, biopsy Gleason Score (GS) other than 3 + 3, 3 + 4, or 4 + 3, or metastasis were also excluded.
Aker et al., 2023 [35]	Unilateral csPCa, age 40– 85 years; PSA <20 ng/mL; prostate volume <70cc; and a life expectancy of at least 10 years. csPCa was defined as PCa ≥ or high-volume GG1 (cancer core length ≥6 mm).	Patients with medical contraindications to MRI, biopsy, or anesthesia, or who had previous treatment for prostate cancer.
Ekish et al., 2013 [36]	Not defined	Not defined
Johansen et al., 2007 [37]	Low-risk patients: T1a-T2a, N0, M0, Gleason score ≤ 6, and prostate-specific antigen (PSA)<10 ng/ml. Intermediate-risk patients were those with T2b tumors or a PSA between 10-20 ng/ml or a Gleason score of 7.	Not defined
Cohen et al., 2008 [38]	Low risk, prostate-specific antigen (PSA) level less than 10 ng/dL, Gleason score less than 7, and Stage T1; moderate risk, PSA level 10 to 20 ng/dL or Stage T2 or Gleason score of 7, with other factors being low risk; and high risk, PSA level of greater than 20 ng/dL, Gleason score of 8 to 10, Stage T3, or any two increased risk factors.	Patients with recurrent prostate cancer after radiotherapy and patients who had undergone hormonal therapy
Dhar et al, 2011 [39]	Not defined	Not defined
Grossgold et al., 2014 [40]	Low risk, PSA<10ng/mL, Gleason score<7, and stage<T2b; moderate-risk, not low or high risk; high-risk, PSA>20ng/mL, Gleason score>7, or stage>T2b.	All patients who had received only post-operative adjuvant hormonal therapy.

Liu et al., 2014 [41]	Not defined	Focal therapy, salvage therapy, pathology other than adenocarcinoma, and advanced prostate cancer (N = 4, T stage (T3a or N stage positive or M-stage positive).
Mercader et al., 2020 [42]	Patients with localized PC over 70 years old, or > 65 years, are unfit for radical prostatectomy.	Not defined
Oishi et al., 2018 [43]	Not defined	Not defined
Tourinho-Barbosa et al., 2020 [44]	Low or intermediate risk PCa (PSA less than 20 ng/ml, GS 7 or less, and clinical stage T2b or less) who received FT using HIFU or cryotherapy.	Patients previously treated with RDT, ADT, or focal or whole gland energy treatment were excluded from the study, as were patients with a follow-up of 6 months or less.
Lepor et al., 2024 [45]	Unilateral intermediate-risk disease Gleason grade group (GGG) 2 or 3), no gross extra-prostatic extension on mpMRI, no GGG ≥2 contralateral to the ROI, and no very distal apical disease on mpMRI.	Not defined
Westhoff et al., 2023 [46]	Suspicious multiparametric MRI (mpMRI) finding (classified either as Prostate Imaging Reporting and Data System (PI-RADS) 3 or as ''suspicious'') as determined and centrally reviewed by experienced in-house radiologists, International Society of Urological Pathologists (ISUP) grade group (GG) 2 PCa proven by combined MRI/TRUS fusion and systematic biopsy or only a previously performed systematic biopsy if cancer positive systematic biopsy cores corresponded to a lesion detected in a later performed mpMRI, prostate-specific antigen (PSA) <10 ng/ml, a normal digital rectal finding (clinical stage cT1c), and life expectancy >10 yr.	Previous PCa treatment, previous radiation to the pelvis, PI-RADS >3 lesion of the contralateral site, and acute urinary tract infection.
Glybochko et al., 2019 [47]	Not defined	Not defined
Ahmed et al., 2011 [48]	Low to intermediate-risk unilateral disease (Gleason 4 3 or less, PSA 15 ng/ml or less, cT2bN0M0 or less) diagnosed by TRUS-guided biopsies who had received no prior treatment.	Bilateral disease
Aoun et al., 2015 [49]	Organ-confined prostate cancer, prostate-specific antigen (PSA) < 20ng/mL, Gleason score ≤ 7(3+4), clinical stage T1N0M0-T2N0M0, and follow-up longer than 12 months.	Patients with incomplete oncologic data.
Blana et al., 2004 [50]	Clinical Stage T1-T2N0M0 biopsy-proven localized prostate cancer, serum prostate-specific antigen (PSA) level less than 15 ng/mL, and a Gleason score of 7 or less.	Not defined

Capogrosso et al., 2018 [51]	Patients were included in the analysis if they were ≥ 70 years, with clinical stage (cT) 1-2 diseases, biopsy Gleason score (GS) either 3 + 3 or 3 + 4, and preoperative PSA ≤ 10 ng/mL.	Preoperative therapy.
Dellabella et al., 2021 [52]	Histologically biopsy-proven clinically significant PCa, stage cT ≤ 2, age ≥45 years, ≥5 years of life expectancy, and grade group (GG) ≤ 3.	Metastatic/nodal disease, radio-recurrent PCa, active rectal diseases, whole-gland ablation, and hormonal treatment. Patient candidates for active surveillance were also excluded.
Duwe et al., 2023 [53]	MpMRI-ultrasound (US)-fusion prostate biopsy of suspicious lesions with a score of ≥ 3 on the Prostate Imaging-Reporting and Data System (PI-RADS). Only patients who had been diagnosed with unilateral PCa of ISUP, grades 1 to 3, were included. Prostate-specific antigen (PSA) ≤ 15 ng/ml, clinical tumor classification ≤ T2, and prostate volume of ≤ 90 ml.	Patients with any previous therapy for PCa (AS, RT, hormone therapy, ablation), patients without prostate-limited disease preoperatively (in terms of extracapsular extension, seminal vesicle invasion, or lymph node metastases), as well as patients diagnosed with PCa after transurethral resection of the prostate.
Fegoun et al., 2011 [54]	Prostate-specific antigen (PSA) ≤ 10 ng/mL, ≤ 3 positive biopsies with only one lobe involved, clinical stage ≤ T2a, Gleason score ≤ 7 with no predominant pattern 4, and negative staging (absence of lymphadenopathy on CT scan and a negative bone scan).	Patients with a previous history of any definitive treatment for prostate cancer or hormonal therapy.
Feijoo et al., 2015 [55]	Unilateral disease, clinical stage T1c–T2a, maximum positive biopsies <33%, Gleason score 7 (3 + 4), prostate-specific antigen (PSA) <15 ng/ml, no extraprostatic extension disease on multiparametric magnetic resonance imaging (mpMRI), and life expectancy >10 yr.	Patients with previous PCa-related treatment.
Ganzer et al., 2018 [56]	Age 18 years or greater, clinical stage T1c-T2a, biopsy-proven unilateral PC, 30% or fewer positive biopsies on systematic TRUS prostate 12-core biopsy with a Gleason score of 3 + 4 = 7 or less, maximum cancer core length 5 mm (only for Gleason score 3 + 4 = 7 cores) and PSA 10 ng/ml or less.	Evidence of significant cancer on the contralateral side on mpMRI as defined by a score of 4 or greater on PI-RADS, version 1 or later version 2, previous prostatic and/or urethral surgery, and/or intake of 5a-reductase inhibitors 6 months or less in duration, and/or previous androgen deprivation therapy.
Hoquetis et al., 2016 [57]	Age < 80 years old; unifocal or multifocal localized PCa (clinical stage ≤ T2N0M0); PSA < 15 ng/mL; Gleason score ≤ 7 and no grade 4 predominant; no prior local or systemic treatment of prostate cancer; and no contraindications to MRI.	Not defined
Nyk et al., 2021 [58]	(1) low or intermediate risk as per EAU risk group definition (T1c–T2b, grade group 1–3, prostate-specific antigen (PSA) 20 ng/mL), (2) not having	Not defined

	undergone prior treatment for PCa, (3) Charlson Comorbidity Index 4 at surgery (not including the points for prostate solid tumor), (4) Eastern Cooperative Oncology Group (ECOG) score at surgery equal to 0.	
Pinthus et al., 2012 [59]	Patients with a clinical stage of T1 and T2, Gleason score of ≤ 7, and serum PSA of < 20 ng/mL.	Patients with a prostate volume of > 40 mL (based on their pre-treatment TRUS at the time of the diagnostic prostate biopsy). Patients who had previously received radiation or HIFU therapy, patients who had received androgen deprivation therapy, and patients who had < 2 consecutive PSA measurements.
Poissonnier et al., 2007 [60]	Localized prostate cancer, clinical stage T1–T2, baseline prostate-specific antigen (PSA) ≤ 15ng/ml, prostate volume ≤ 40 cc, no previous radical treatment for prostate cancer, and at least one year of follow-up.	Not defined
Ghai et al., 2021 [61]	Age: 50 years or older, and had intermediate-risk prostate cancer, a prostate-specific antigen (PSA) level of 20 ng/mL or less, and less than 20 mm of MRI-visible GG 2 or GG 3 disease at transrectal US-guided systematic and targeted biopsy.	Contraindications to MRI, intraductal carcinoma, a second site of a tumor visible at MRI, previous treatment for prostate cancer, calcification of 2 mm or greater at CT adjacent to the rectal wall or in the beam path, or if the tumor location was less than 6 cm from the rectal wall.
Rischmann et al., 2016 [62]	Treatment-naive patients with T1/T2 clinical stage were considered for inclusion. Gleason score ≤ 7 (3 + 4).	Patients with a biopsy-confirmed mpMRI lesion located <6 mm from the apex or <5 mm from the sagittal midline were excluded.
Sivaraman et al., 2020 [63]	Clinical stage T1c--T2a, maximum 33% of biopsy cores involved by PCa, Gleason ≤7 (3+4), PSA <15ng/mL, absence of extra-prostatic extension and seminal vesicle invasion and pelvic lymph node involvement at mpMRI, and patient's life expectancy higher than ten years.	Men with anterior and/or apical lesions or men with prostate calcifications and/or cysts possibly interfering with optimal HIFU energy delivery were excluded.
Velthoven et al., 2016 [64]	Men with localized prostate cancer (clinical stage ≤ T2), a PSA < 15 ng/ml, a life expectancy of at least five years, and a prostate volume of 40cm3.	Patients who had an extra-prostatic extension on multiparametric MRI suspected regional lymph nodes or distant metastases on cross-sectional imaging or bone scan and/or previous HIFU or radiation therapy to the prostate.
Hardenberg et al., 2018 [65]	Prostate-specific antigen (PSA) < 10 ng/ml and Gleason score 3+4 for all patients, and either two cancer-positive prostate imaging reporting and data system (PI-RADS) 3-5 target lesions on mpMRI or biopsy-proven cancer limited to one prostate zone without a PI-RADS lesion on mpMRI.	Tumor locations out of the anterior-posterior distance of the device (40 mm) or too close to the sphincter were excluded.

Arnouil et al., 2018 [66]	Localized cancer: clinical stage T1 or T2, Gleason score ≤ 3 + 4 = 7, PSA < 15 ng/mL, first-line treatment, absence of extraprostatic extension on MRI, and life expectancy of 10 years.	Not defined
Crouzet et al., 2011 [67]	Stage T1-T2, PSA ≤ 15, Gleason ≤ 7.	Not defined
Luca et al., 2023 [68]	Men with low-risk PCa (who refuse active surveillance) and intermediate-risk PCa, according to EAU guidelines, at prostate target biopsy with previously positive multiparametric MRI (mpMRI) and concomitant negative standard biopsy or two positive standard biopsy homolateral and/or contralateral to the target ones; men with negative multiparametric MRI and standard biopsy-proven low or intermediate-risk cancer.	Contraindications to MRI or prostate volume greater than 70 cc as measured at MRI; Gleason Grade Group (GG) >2 (Gleason Score >3+4); severe systemic disease, previous neoadjuvant androgen deprivation therapy, severe anal strictures, inflammatory bowel disease and contraindication to general or epidural anesthesia.
Misraï et al., 2008 [69]	Clinical stage T1/T2 (1998 TNM classification), normal bone scintigraphy, normal abdominal CT, and refusal of other treatment options.	In case of lymph node invasion, HIFU therapy was systematically avoided. Any previous treatment for prostate cancer.
Rosenhammer et al., 2019 [70]	All patients with clinically localized PCa and adequate oncological follow-up data (life status, cause-specific mortality, biochemical relapse, and salvage treatment) were consecutively included	Advanced cT3 stage.
Shoji et al., 2020 [71]	(a) serum PSA levels ≤20 ng/ml, (b) significant PCa that had been located using MRI-TRUS elastic fusion image-guided transperineal prostate biopsy and 12-cores transperineal systematic biopsy, (c) life expectancies longer than 10 years, (d) no metastasis, (e) no bilateral cancers with Gleason scores ≥7, (f) no severe anal strictures, and (g) no previous history of treatment for PCa	Patients who had the lesion includes urethra and bilateral PZ close to NVB, the severe anal stricture, and difficulty to understand what the focal therapy is.
Rivera et al., 2018 [72]	Prostate cancer diagnosed by 12-cylinder transrectal biopsy, TNM stage T1-T2cN0M0, prostatic volume less than 50 cc, intraprostatic calcifications no larger than 1 cm, PSA ≤ 15 ng/ml.	Non-localized prostate cancer (T3N+M+), previous rectal surgery (cured hemorrhoidectomy and anal fistulorrhaphy do not exclude the patient), current rectal disease, stricture of the anal canal, active inflammatory bowel disease, rectal or urinary fistula, active UTI or prostatitis, prostatic calcifications greater than 1 cm or very abundant microcalcifications, presence of ureteral stent or an anteroposterior prostatic diameter greater than 4 cm.
Wu et al., 2020 [73]	Not defined	Not defined

Abreu et al., 2020 [74]	Hemigland HIFU as primary treatment for PCa; biopsy-proven unilateral PCa or selected men with bilateral PCa, where the contralateral lobe had nondominant, low-volume, GG1 PCa; and localized PCa (patients with clinical T3 on DRE were not offered partial gland ablation).	Patients undergoing three-quarters, subtotal, whole gland or salvage HIFU.
Chen et al., 2018 [75]	Not defined	Not defined
Dickinson et al., 2016 [76]	Not defined	Not defined
Reddy et al., 2022 [77]	Patients with Gleason Score 6-9 prostate cancer, and radiological stage up to T3bN0M0 were offered focal therapy. Only patients with MRI visible lesions and no high-volume (>/=6mm) Gleason score 3+3=6 or any volume Gleason score >/=3+4=7 disease in areas to be left untreated were considered suitable for focal ablation.	Not defined
Komura et al., 2013 [78]	Clinical stage T1-T2N0M0 biopsy-proven localized prostate cancer, prostate volume at diagnosis B50 ml, and no previous treatment for prostate cancer with curative intent.	Salvage treatment
Limani et al., 2014 [79]	Whole gland primary therapy with curative intent for localized PCa, prostate specific antigen (PSA) <20ng/mL, clinical stage T1N0M0-T2N0M0, no previous radical therapy for PCa, and a follow-up >12 months.	Clinically advanced PCa, evidence of metastatic or nodal disease on bone scan or cross-sectional imaging, prior significant rectal surgery, any contraindication for anesthesia, and presence of prostatic calcification and cysts.
Mearini et al., 2014 [80]	Patients with T1c–T2 and limited cT3a N0M0 disease.	Prostate volume exceeding 50 ml, intraprostatic calcification greater than 1 cm and concomitant anal stricture.
Pfeiffer et al., 2012 [81]	Low risk: clinical stage T1 – T2a, preoperative PSA ≤ 10 ng/mL and biopsy Gleason score ≤ 6; intermediate risk: stage T2b or PSA > 10 to ≤ 20 ng/mL or Gleason score 7; and high risk: stage T2C or PSA > 20 ng/mL or Gleason score ≥ 8.	Patients with nodal extension or metastatic disease.
Ripert et al., 2010 [82]	Low risk: clinical stage T1c or T2a, Gleason score < 6 and PSA ≤ 10 ng/mL; intermediate risk: PSA 10–20 ng/mL, Gleason score of 7, or clinical stage T2b.	Patients in whom HIFU treatment was incomplete because of a technical incident, excessive rectal wall thickness, or ultrasonography detection problems, and who were not offered a second HIFU session.
Tsai et al., 2023 [83]	Not defined	Not defined
Ploussard et al., 2024 [84]	Patients with localized, low- to low-to-intermediate-risk PC (clinical stage T1–2 Nx M0) with prostate-specific antigen (PSA) <15 ng/ml, Gleason score 3 + 4 (grade group 1 or 2), are not eligible for active surveillance (AS), and a	Not defined

	maximum of four sextants out of six with invasion on biopsies performed following multiparametric magnetic resonance imaging.	
Nahar et al., 2024 [85]	Patients with localized PC are eligible for focal or hemigland ablation; All risk groups; Patients with bilateral prostatic disease were included if they had clinically nonsignificant PC (GG 1) on the contralateral side to the index lesion.	Patients with high-risk PC with more than two cores containing a Gleason grade group (GG) 4 PC and no identifiable lesion on MRI.

Table S4. Basic characteristics of included studies regarding irreversible electroporation (IRE).

Author (year)	Study site	Study design	Study period	Modality subgroup	Device	Study participation	Age (years) ‡	Gleason score (No: ≤6; 7)	NCCN risk category (No: low; intermediate)	Clinical stage (No: T1; T2)	Preoperative PSA (ng/mL) ‡
Rosette et al. (2023) [1]	Netherland	RCT	2015-2020	Focal	NanoKnife (Angiodynamics)	51	64 (58;64)	28;23	28;23	45;6	5.93 (4.34;8.96)
				Extended	NanoKnife (Angiodynamics)	55	64 (57;68)	32;23	32;23	49;6	6.05 (4.5;8.64)
Collettini et al. (2019) [2]	Germany	Prospective cohort	2014-2017	Focal	NanoKnife (Angiodynamics)	30	65.5 (60; 68.8)	7;23	4;26	NA	8.65 (5;11)
Wang et al. (2022) [3]	China	Prospective cohort	2018-2019	Extended	Remedicine Co	109	67 (8)	47;62	27;82	32;77	9 (6;12.7)
Shin et al. (2023) [4]	South -Korea	Prospective cohort	2021-2022	Focal	NanoKnife (Angiodynamics)	17	66.1 (9.3)	11;6	10;7	0;17	7.5 (3.9)
Giganti et al. (2019) [5]	UK	Retrospective cohort	2011-2016	Focal	NanoKnife (Angiodynamics)	30	63 (60;67)	7;23	NA	NA	NA
Blazevski et al. (2019) [6]	Australia	Prospective cohort	2013-2018	Focal	NanoKnife (Angiodynamics)	123	68 (62;73)	12;111	11;112	NA	5.73 (3.8;8)
Popeneciu et al., 2024 [7]	Germany	Prospective cohort	2018-2021	Focal	NanoKnife (Angiodynamics)	24	65.2 (4.9)	14;10	14;10	NA	7.9 (3.5)
Yaxley et al., 2022 [8]	Australia	Retrospective cohort	2018-2021	Focal	NanoKnife (Angiodynamics)	52	72 (51-87)	4;48	4;48	NA	NA
Altan et al., 2023 [9]	Turkey	Prospective cohort	2020-2023	Focal	NanoKnife (Angiodynamics)	18	61.1 (6.5)	NA	5;13	NA	6.73 (2.98)

Murray et al. (2016) [10]	USA	Prospective cohort	2011-2014	Focal	NanoKnife (Angiodynamics)	25	63.1 (59.3;67.6)	18;7	18;7	NA	4.3 (3.3;5.6)
Ting et al. (2015) [11]	Australia	Prospective cohort	2013-2014	Focal	NanoKnife (Angiodynamics)	25	67 (60; 71)	2;23	2;23	11;14	6 (4.3;8.6)
Valerio et al. (2016) [12]	UK	Prospective cohort	2013-2015	Focal	NanoKnife (Angiodynamics)	19	60 (53; 66)	8;11	7;12	18;1	7.75 (5.5; 10.03)
Bos et al. (2017) [13]	Australia	Prospective cohort	2013-2016	Focal	NanoKnife (Angiodynamics)	63	67 (61-71)	9;54	8;55	NA	6 (3.2;8.4)
López et al. (2023) [14]	Spain	Prospective cohort	2014-2021	Focal	NanoKnife (Angiodynamics)	41	65.8 (8.34)	30;11	NA	NA	6.9 (2.76)

‡ Parameters are represented as mean with standard deviation (SD) or median with interquartile range (IQR min; IQR max) or range (minimum-maximum). UK: United Kingdom; USA: United States of America; RCT: randomized controlled trial; NCCN: National Comprehensive Cancer Network; PSA: Prostate-specific antigen; NA: not applicable

Table S5. Basic characteristics of included studies regarding cryoablation.

Author (year)	Study site	Study design	Study period	Modality subgroup	Device	Study participation	Age (years) ‡	Gleason score (No: ≤6; 7)	NCCN risk category (No: low; intermediate)	Clinical stage (No: T1; T2)	Preoperative PSA (ng/mL) ‡
Tokuda et al. (2023) [15]	Japan	Retrospective cohort	2017-2021	Focal	CryoHit	16	69 (51-81)	0;16	0;16	0;16	8.22 (4.14-14)
Wysock et al. (2020) [16]	USA	Prospective cohort	2017-2019	Partial/Hemi/Whole	Cryocare CS® system	83	64 (59;70)	9;74	9;74	NA	6.18 (4.6;7.8)
Sze et al. (2019) [17]	USA	Retrospective cohort	2012-2016	Focal	NA	17	NA	12;5	12;5	NA	8.7 (6.7;11.76)
Kim et al. (2012) [18]	Brazil	Retrospective cohort	2010-2011	Partial/Hemi/Whole	Cryocare CS® system	10	66.2 (10.8)	6;4	5;5	NA	7.8 (2.8)
Bahn et al. (2012) [19]	USA	Retrospective cohort	2002-2010	Focal	Endocare	73	64 (47-79)	30;43	24;49	41;32	5.9 (3.9)
Barqawi et al., (2017) [20]	USA	Prospective cohort	2007-2015	Focal/Partial/Whole	NA	393	65 (60;71)	NA	NA	NA	NA

Hayek et al. (2008) [21]	Brazil	Prospective cohort	2000-2004	Focal	Cryocare CS® system	13	70.9 (55-83)	NA	13;0	NA	5.5 (NA)
Gregg et al. (2021) [22]	USA	Prospective cohort	2009-2012	Partial/Hemi/Whole	NA	23	62.2 (6.8)	18;5	18;5	NA	3.9 (2.1)
Lambert et al. (2007) [23]	USA	Retrospective cohort	2002-2005	Focal	NA	25	69 (48-78)	13;12	NA	25;0	6 (1-13.1
Lian et al. (2015) [24]	China	Retrospective cohort	2006-2013	Focal	CryoHit	41	67 (56-76)	24;17	23;18	26;15	7.1 (2.6-14.1)
Mendez et al. (2015) [25]	USA	Registry	2007-2013	Focal	Endocare	317	66.5 (6.6)	317;0	317;0	NA	NA
				Partial/Hemi/Whole	Endocare	317	66.5 (6.6)	317;0	317;0	NA	NA
Marra et al. (2021) [26]	France	Prospective cohort	2008-2018	Focal	Cryocare CS® system	121	66 (62;71)	92;29	92;29	101;20	6.42 (5.03;8.08)
Barret et al. (2013) [27]	France	Prospective cohort	2009-2011	Focal	Galil Medical	50	66.5 (61;73)	50;0	50;0	NA	6.2 (5;7.9)
Enikeev et al. (2020) [28]	Russia	Prospective cohort	2016-2017	Partial/Hemi/Whole	SeedNet Gold	45	64.4 (3.8)	45;0	45;0	NA	8.6 (1.2)
Durand et al., 2014 [29]	France	Prospective cohort	2009-2012	Hemigland	Galil Medical	48	66.6 (50.4-77.1)	48;0	48;0	42;6	6.1 (4.9;7.1)
Hale et al., 2013 [30]	USA	Prospective cohort	2006-2012	Hemigland/Subtotal	Cryocare CS® system	26	65	25;1	23;3	26;0	NA
Boissier et al., 2020 [31]	Spain	Prospective cohort	2010-2018	Hemigland/Whole	Galil Medical	66	76 (71-80); 74 (42-81)	20;46	12;54	44;22	7.9 (3.3-11.9); 6.7 (1.2-11.6)
Rodríguez et al., 2014 [32]	Spain	Prospective cohort	2001-NA	Whole	Stryker Cryo/44	62	NA	NA	28;34	NA	NA
Elkjær et al., 2014 [33]	Denmark	Prospective cohort	2006-2012	Whole	SeedNet Gold	27	NA	NA	6;21	NA	NA
Guo et al., 2020 [34]	USA	Registry	2004-2015	Focal/Whole	NA	1942	68.6 (7.4)	939;1003	805;1137	1645;297	NA

Aker et al., 2023 [35]	USA	Prospective cohort	2017-2021	Partial	Galil Medical	97	NA	NA	97;0	NA	NA
Ekish et al., 2013 [36]	USA	Retrospective cohort	2008-2011	Focal/Whole	Cryocare CS® system	21	68 (54-89)	NA	10;21	NA	NA
Johansen et al., 2007 [37]	Norway	Retrospective cohort	2003-2007	Whole	Galil Medical	64	64.65 (53-75)	NA	27;37	NA	8.2 (0.5-16)
Cohen et al., 2008 [38]	USA	Retrospective cohort	1991-1996	Whole	Cryocare CS® system	116	NA	NA	36;80	NA	NA
Dhar et al, 2011 [39]	USA	Registry	NA	Whole	NA	576	NA	NA	127;446	NA	NA
Grossgold et al., 2014 [40]	USA	Registry	NA	Whole	NA	2090	NA	NA	682;1408	NA	NA
Liu et al., 2014 [41]	Taiwan	Retrospective cohort	2008-2013	Whole	Cryocare CS® system	43	NA	NA	19;24	NA	NA
Mercader et al., 2020 [42]	Spain	Retrospective cohort	2008-2017	Whole	Cryocare CS® system	148	NA	NA	57;91	NA	NA
Oishi et al., 2018 [43]	USA	Retrospective cohort	2002-2012	Whole	Cryocare CS® system	69	NA	NA	24;45	NA	NA
Tourinho-Barbosa et al., 2020 [44]	France	Retrospective cohort	2009-2018	Focal/Whole	Galil Medical	119	66 (62;71)	91;28	79;40	NA	6.5 (5-8.3)
Lepor et al., 2024 [45]	USA	Prospective cohort	2017-2024	Partial	Cryocare CS® system	313	65 (60.9;70.1)	NA	0;313	NA	NA

‡ Parameters are represented as mean with standard deviation (SD) or median with interquartile range (IQR min; IQR max) or range (minimum-maximum). USA: United States of America; NCCN: National Comprehensive Cancer Network; PSA: Prostate-specific antigen; NA: not applicable.

Table S6. Basic characteristics of included studies regarding high-intensity focused ultrasound (HIFU).

Author (year)	Study site	Study design	Study period	Modality subgroup	Device	Study participation	Age (years) ‡	Gleason score (No: ≤6; 7)	NCCN risk category (No: low; intermediate)	Clinical stage (No: T1; T2)	Preoperative PSA (ng/mL) ‡
Westhoff et al. (2023) [46]	Germany	Prospective cohort	2014-2020	Focal	Focal One	50	68 (63;74)	27;23	35;15	50;0	6.5 (4.9;8.3)
Glybochko et al. (2019) [47]	Russia	Retrospective cohort	2013-2016	Hemiablation	Ablatherm	35	65 (NA)	NA	NA	35;0	6 (1.5)
Ahmed et al. (2011) [48]	UK	Prospective cohort	2006-2008	Hemiablation	Sonablate	20	60.4 (5.4)	NA	5;15	NA	7.3 (2.8)
Aoun et al. (2015) [49]	Belgium	Retrospective cohort	2001-2012	Whole	Ablatherm	70	74 (62;86)	51;19	31;39	39;31	12.1 (4.1)
Blana et al. (2004) [50]	Germany	Retrospective cohort	1997-2002	Whole	Ablatherm	146	66.9 (6.7)	NA	NA	NA	7.6 (3.4)
Capogrosso et al. (2018) [51]	France	Prospective cohort	2005-2014	Whole	Ablatherm	84	72 (72;76)	56;28	47;37	44;40	6.32 (5.2-7.7)
Dellabella et al. (2021) [52]	Italy	Prospective cohort	2017-2019	Focal/Hemiablation	Focal One	189	70 (59;81)	92;97	NA	NA	5.8 (2.8;8.8)
Duwe et al. (2023) [53]	Germany	Prospective cohort	2016-2021	Focal/Hemiablation	Focal One	29	66 (61.5;72.5)	20;9	NA	NA	6.8 (5.1;8.9)
Fegoun et al. (2011) [54]	France	Retrospective cohort	1997-2000	Hemiablation	Ablatherm	12	70 (4.8)	10;2	0;12	NA	7.3 (2.44)
Feijoo et al., (2015) [55]	France	Prospective cohort	2009-2013	Hemiablation	Ablatherm	67	70.2 (6.8)	58;9	NA	NA	6.1 (1.6;15.5)
Ganzer et al. (2018) [56]	Germany	Prospective cohort	2013-2016	Hemiablation	Focal One (40), Ablatherm (11)	51	63.4 (8.3)	43;8	NA	NA	6.2 (2.1)
Hoquetis et al. (2016) [57]	France	Retrospective cohort	2009-2014	Hemiablation	Ablatherm	25	66 (7.3)	19;6	NA	17;8	6.13 (2.5)
Nyk et al. (2021) [58]	Poland	Retrospective cohort	2016-2019	Focal/Hemiablation	Sonablate	30	64.5 (NA;NA)	20;10	NA	17;13	6.6 (NA)

Pinthus et al. (2012) [59]	Canada	Retrospective cohort	2005-2010	Focal	Ablatherm	402	62.7 (7.5)	209;193	183;219	309;93	6.6 (3.1)
Poissonnier et al. (2007) [60]	France	Retrospective cohort	1993-2003	Hemiablation/Whole	Ablatherm	227	68.8 (5.82)	152;75	NA	122;105	6.99 (3.48)
Ghai et al. (2021) [61]	Canada	Prospective cohort	2016-2019	Focal	Exablate 2100 Prostate	44	67 (62;70)	0;44	0;44	NA	6.4 (4.3;9.6)
Rischmann et al. (2016) [62]	France	Prospective cohort	2009-2014	Hemiablation	Ablatherm	111	64.8 (6.2)	82;29	75;36	77;34	6.2 (2.5)
Sivaraman et al. (2020) [63]	France	Retrospective cohort	2009-2019	Hemiablation (Cohort 1: US-guided)	Ablatherm	88	68 (6.9)	73;15	NA	NA	7.1 (2.9)
	France			Hemiablation (Cohort 2: MRI-guided)	Focal One	54	67.6 (7.3)	36;16	NA	NA	7.7 (2.6)
Velthoven et al. (2016) [64]	Belgium	Prospective cohort	2007-2015	Hemiablation	Ablatherm	50	74 (70;77)	30;20	24;26	16;34	6.3 (3.9-8.3)
Hardenberg et al. (2018) [65]	Germany	Prospective cohort	2014-2016	Focal	Focal One	24	70 (52-78)	17;7	NA	NA	6.53 (0.99-9.36)
Arnouil et al. (2018) [66]	France	Retrospective cohort	2008-2014	Focal/Hemiablation	Focal One, Ablatherm	53	65.11 (6.08)	40;13	33;20	47;6	6.32 (2.68)
Crouzet et al. (2011) [67]	France	Retrospective cohort	2005-2009	Whole	Ablatherm	297	71.4 (5.1)	190;107	149;148	172;125	6.49 (3.43)
Luca et al. (2023) [68]	Italy	Prospective cohort	2018-2020	Focal/Hemiablation/Whole	Focal One	100	73.7 (6.6)	33;67	33;67	NA	5.92 (2.5)
Barret et al. (2013) [27]	France	Prospective cohort	2009-2011	Hemiablation	Ablatherm	21	66.5 (60;73)	21;0	21;0	NA	6 (5.1;8.1)
Enikeev et al. (2020)[28]	Russia	Prospective cohort	2016-2017	Whole	Ablatherm	45	63.9 (3.7)	45;0	45;0	NA	8.7 (0.9)
Tourinho-Barbosa et al., 2020 [44]	France	Retrospective cohort	2009-2018	Focal/Hemiablation	Ablatherm	190	68 (62;73)	130;60	103;87	NA	7.1 (5.5;9)
Misraï et al., 2008 [69]	France	Retrospective cohort	2001-2006	Whole	Ablatherm	115	NA	NA	65;50	NA	NA
Rosenhammer et al., 2019 [70]	Germany	Retrospective cohort	1997-2009	Whole	Ablatherm	402	NA	NA	209;193	NA	NA

Shoji et al., 2020 [71]	Japan	Prospective cohort	2016-2018	Focal	Sonablate	75	68.76 (39-85)	46;29	31;44	0;75	7.5 (2.48-19.05)
Rivera et al., 2018 [72]	Spain	Retrospective cohort	2007-2016	Whole	Sonablate	65	NA	40;25	35;30	NA	NA
Wu et al., 2020 [73]	Taiwan	Retrospective cohort	2009-2015	Whole	Ablatherm	66	NA	35;31	14;52	NA	NA
Abreu et al., 2020 [74]	USA	Retrospective cohort	2015-2019	Hemiablation	Sonablate	69	NA	NA	25;44	NA	NA
Chen et al., 2018 [75]	Taiwan	Retrospective cohort	2009-2015	Whole	Ablatherm	85	NA	NA	23;62	NA	NA
Dickinson et al., 2016 [76]	UK	Registry	2004-2012	Whole	Sonablate	532	NA	NA	161;371	NA	NA
Reddy et al., 2022 [77]	UK	Registry	2005-2020	Focal	Sonablate	916	NA	NA	20;896	NA	NA
Komura et al., 2013 [78]	Japan	Prospective cohort	2004-2008	Whole	Sonablate	99	NA	NA	52;47	NA	NA
Limani et al., 2014 [79]	Belgium	Retrospective cohort	2001-2012	Whole	Ablatherm	89	NA	NA	40;49	NA	NA
Mearini et al., 2014 [80]	Italy	Prospective cohort	2004-2007	Whole	Sonablate	127	NA	NA	80;47	NA	NA
Pfeiffer et al., 2012 [81]	Germany	Retrospective cohort	2002-2006	Whole	Ablatherm	138	NA	NA	72;66	NA	NA
Ripert et al., 2010 [82]	France	Retrospective cohort	2004-2010	Whole	Ablatherm	53	72.5 (60-79)	43;10	28;25	34;19	8.5 (4)
Tsai et al., 2023 [83]	Taiwan	Retrospective cohort	2021-2022	Whole	Sonablate	43	NA	NA	15;28	NA	NA
Ploussard et al., 2024 [84]	France	Prospective cohort	2015-2019	Whole	Ablatherm	1967	74.7 (72.4;77.6)	NA	1109;858	NA	NA
Nahar et al., 2024 [85]	USA	Prospective cohort	2016-2023	Focal/Hemiablation	NA	80	NA	NA	24;56	NA	NA

‡ Parameters are represented as mean with standard deviation (SD) or median with interquartile range (IQR min; IQR max) or range (minimum-maximum). UK: United Kingdom; NCCN: National Comprehensive Cancer Network; PSA: Prostate-specific antigen; NA: not applicable.

Table S7. Functional outcomes (newly developed urinary incontinence and sexual function).

	IRE (%)		Cryoablation (%)		HIFU (%)	
	**Focal**	**Extended**	**Focal**	**Whole-gland**	**Focal**	**Whole-gland**
De novo urinary incontinence	0-14	NA	0-15	0-23	0-20	0-22
De novo erectile dysfunction	0-24		0-31	0-53	0-33	12-53

IRE: irreversible electroporation; HIFU: high-intensity focused ultrasound; NA: not applicable.

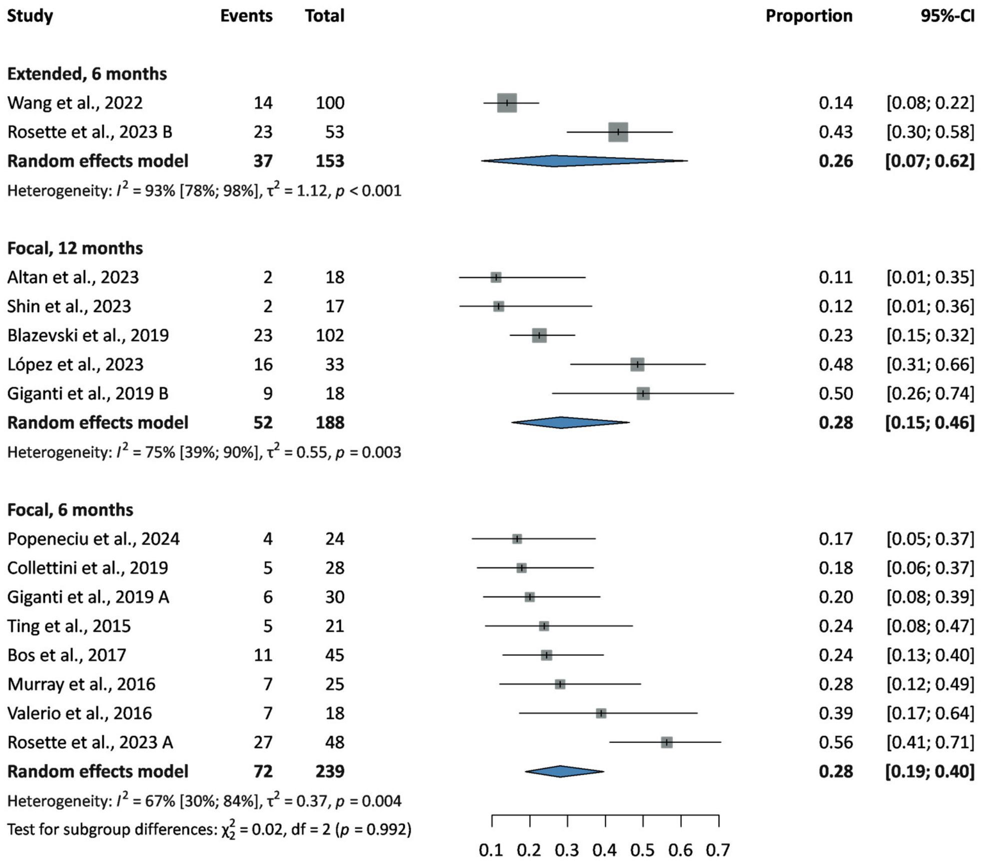

Figure S1. Summary forest plot of 6 and 12-month recurrence rates regarding focal and extended irreversible electroporation [1-4, 6, 7, 9-14].

Study	Follow up time	Events	Total	Proportion	95%-CI	Weight
Ting et al., 2015	6 months	0	21	0.00	[0.00; 0.16]	2.09%
Popeneciu et al., 2024	6 months	1	24	0.04	[0.00; 0.21]	3.82%
Altan et al., 2023	12 months	1	18	0.06	[0.00; 0.27]	3.77%
Shin et al., 2023	12 months	1	17	0.06	[0.00; 0.29]	3.76%
Blazevski et al., 2019	12 months	10	102	0.10	[0.05; 0.17]	16.69%
Yaxley et al., 2022	Median 23 months	5	43	0.12	[0.04; 0.25]	11.78%
López et al., 2023	12 months	5	33	0.15	[0.05; 0.32]	11.50%
Bos et al., 2017	6 months	7	45	0.16	[0.06; 0.29]	13.79%
Murray et al., 2016	6 months	4	25	0.16	[0.05; 0.36]	9.96%
Collettini et al., 2019	6 months	5	28	0.18	[0.06; 0.37]	11.28%
Valerio et al., 2016	6 months	7	18	0.39	[0.17; 0.64]	11.56%
**Random effects model**		**46**	**374**	**0.14**	**[0.09; 0.19]**	**100.00%**

Heterogeneity: $I^2 = 36\%$ [0%; 68%], $\tau^2 = 0.17$, $p = 0.114$

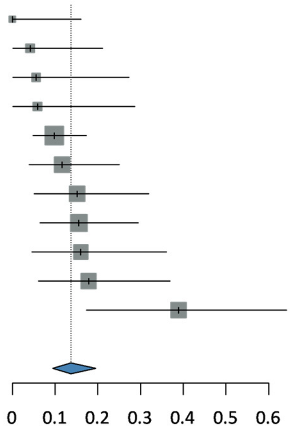

Figure S2. Summary forest plot of pooled in-field recurrence rates regarding focal irreversible electroporation [2, 4, 6-14].

Study	Follow up time	Events	Total	Proportion	95%-CI	Weight
Collettini et al., 2019	6 months	0	28	0.00	[0.00; 0.12]	2.91%
Valerio et al., 2016	6 months	0	18	0.00	[0.00; 0.19]	2.89%
Altan et al., 2023	12 months	1	18	0.06	[0.00; 0.27]	4.90%
Shin et al., 2023	12 months	1	17	0.06	[0.00; 0.29]	4.89%
Bos et al., 2017	6 months	4	45	0.09	[0.02; 0.21]	10.88%
Murray et al., 2016	6 months	3	25	0.12	[0.03; 0.31]	9.36%
Popeneciu et al., 2024	6 months	3	24	0.12	[0.03; 0.32]	9.33%
Blazevski et al., 2019	12 months	13	102	0.13	[0.07; 0.21]	15.31%
Ting et al., 2015	6 months	5	21	0.24	[0.08; 0.47]	11.08%
Yaxley et al., 2022	Median 23 months	13	43	0.30	[0.17; 0.46]	14.60%
López et al., 2023	12 months	11	33	0.33	[0.18; 0.52]	13.85%
**Random effects model**		**54**	**374**	**0.15**	**[0.09; 0.22]**	**100.00%**

Heterogeneity: $I^2 = 57\%$ [16%; 78%], $\tau^2 = 0.37$, $p = 0.009$

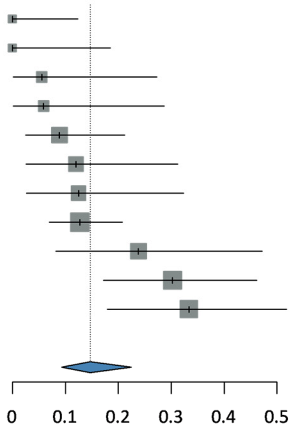

Figure S3. Summary forest plot of pooled out-field recurrence rates regarding focal irreversible electroporation [2, 4, 6-14].

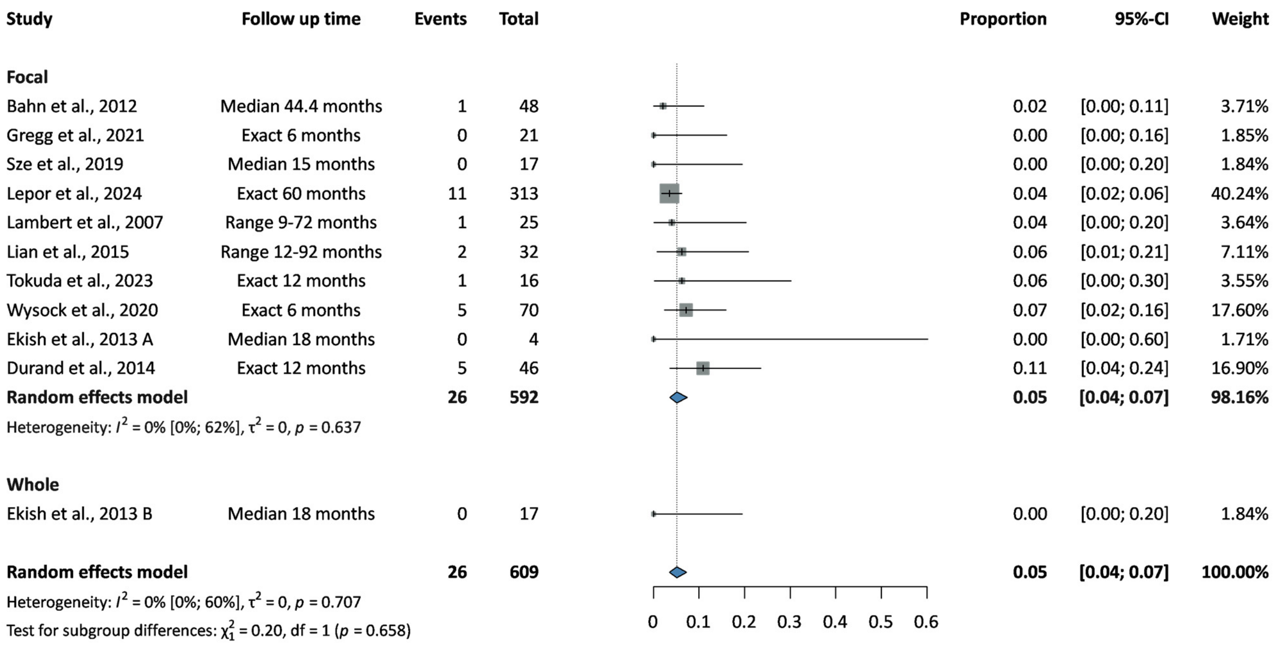

Figure S4. Summary forest plot of pooled in-field recurrence rates regarding cryoablation [15-17, 19, 22-24, 29, 36, 45].

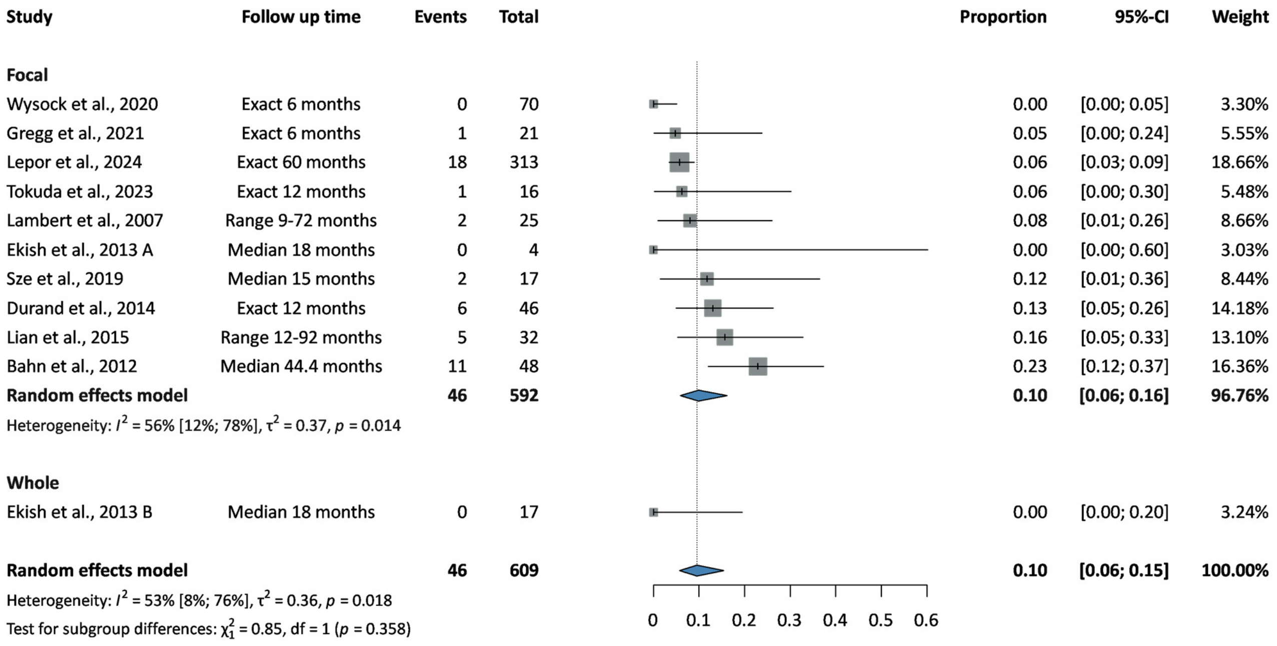

Figure S5. Summary forest plot of pooled out-field recurrence rates regarding cryoablation [15-17, 19, 22-24, 29, 36, 45].

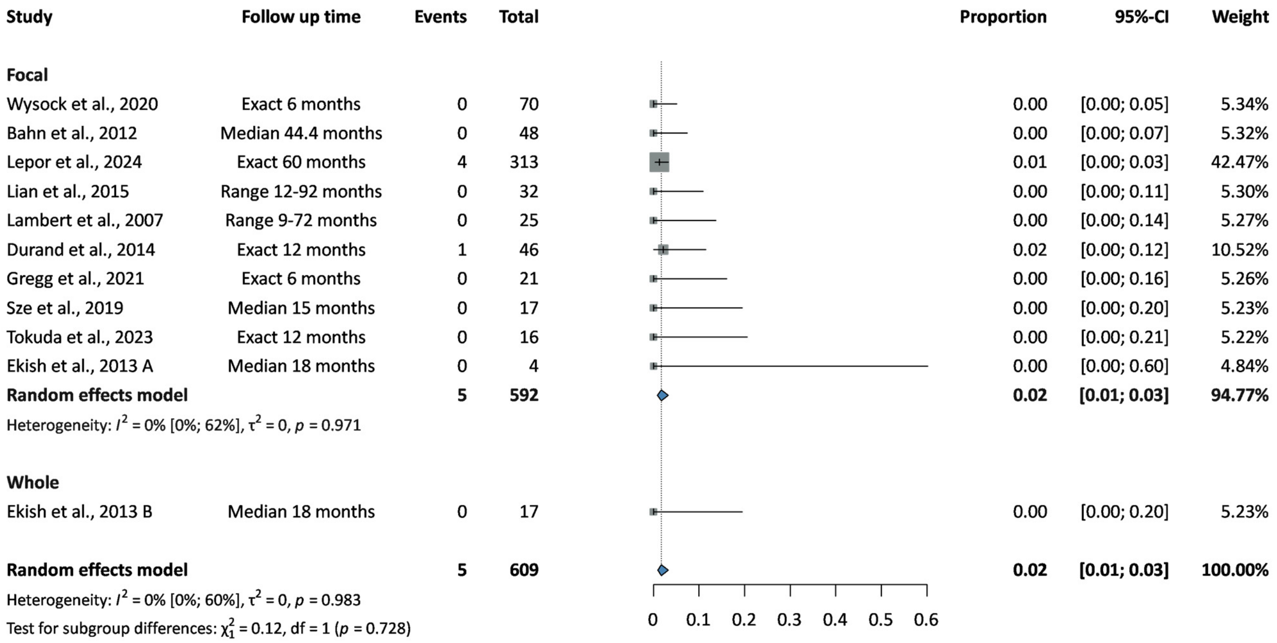

Figure S6. Summary forest plot of pooled concurrent in- and out-field recurrence rates regarding cryoablation [15-17, 19, 22-24, 29, 36, 45].

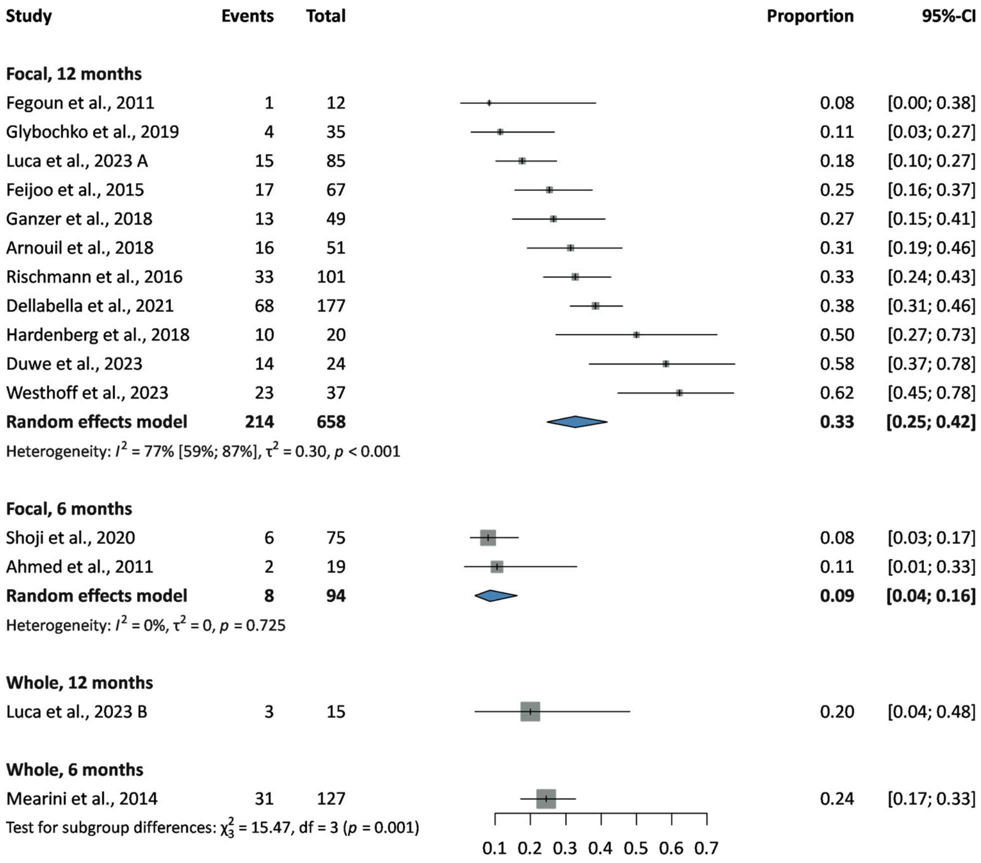

Figure S7. Summary forest plot of 12-month recurrence rates regarding focal high-intensity focused ultrasound [46-48, 52-56, 62, 65, 66, 68, 71, 80].

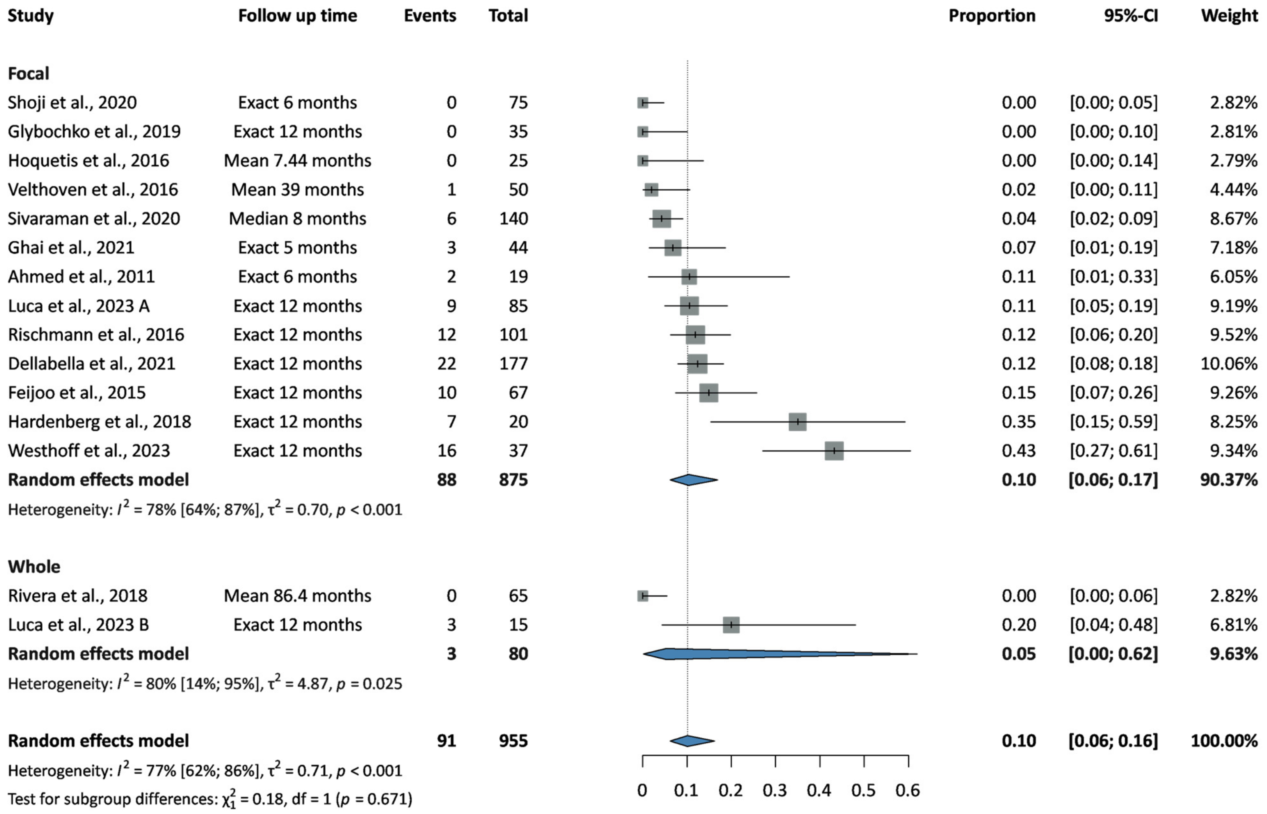

Figure S8. Summary forest plot of pooled in-field recurrence rates regarding focal and whole gland high-intensity focused ultrasound [46-48, 52, 55, 57, 61-65, 68, 71, 72].

Study	Follow up time	Events	Total	Proportion	95%-CI	Weight
**Focal**						
Sivaraman et al., 2020	Median 8 months	0	140	0.00	[0.00; 0.03]	2.66%
Ghai et al., 2021	Exact 5 months	0	44	0.00	[0.00; 0.08]	2.64%
Ahmed et al., 2011	Exact 6 months	0	19	0.00	[0.00; 0.18]	2.61%
Velthoven et al., 2016	Mean 39 months	3	50	0.06	[0.01; 0.17]	7.44%
Luca et al., 2023 A	Exact 12 months	6	85	0.07	[0.03; 0.15]	9.20%
Shoji et al., 2020	Exact 6 months	6	75	0.08	[0.03; 0.17]	9.17%
Westhoff et al., 2023	Exact 12 months	3	37	0.08	[0.02; 0.22]	7.37%
Feijoo et al., 2015	Exact 12 months	6	67	0.09	[0.03; 0.18]	9.15%
Hardenberg et al., 2018	Exact 12 months	2	20	0.10	[0.01; 0.32]	6.10%
Glybochko et al., 2019	Exact 12 months	4	35	0.11	[0.03; 0.27]	8.08%
Hoquetis et al., 2016	Mean 7.44 months	4	25	0.16	[0.05; 0.36]	7.93%
Rischmann et al., 2016	Exact 12 months	19	101	0.19	[0.12; 0.28]	10.87%
Dellabella et al., 2021	Exact 12 months	46	177	0.26	[0.20; 0.33]	11.52%
**Random effects model**		**99**	**875**	**0.10**	**[0.06; 0.15]**	**94.75%**
Heterogeneity: I^2 = 73% [52%; 84%], τ^2 = 0.50, p < 0.001						
**Whole**						
Rivera et al., 2018	Mean 86.4 months	0	65	0.00	[0.00; 0.06]	2.65%
Luca et al., 2023 B	Exact 12 months	0	15	0.00	[0.00; 0.22]	2.60%
**Random effects model**		**0**	**80**	**0.02**	**[0.00; 0.10]**	**5.25%**
Heterogeneity: I^2 = 0%, τ^2 = 0, p = 0.476						
**Random effects model**		**99**	**955**	**0.09**	**[0.05; 0.14]**	**100.00%**
Heterogeneity: I^2 = 72% [53%; 83%], τ^2 = 0.56, p < 0.001						
Test for subgroup differences: χ^2_1 = 3.43, df = 1 (p = 0.064)						

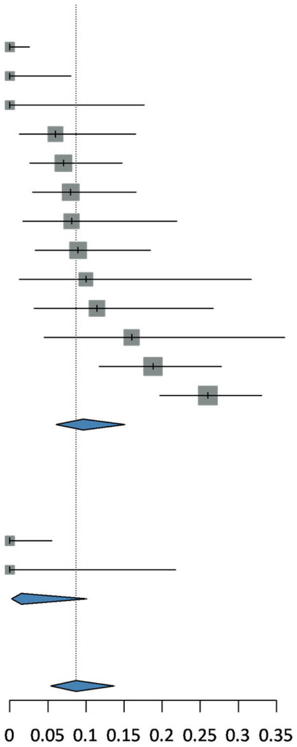

Figure S9. Summary forest plot of pooled out-field recurrence rates regarding focal and whole gland high-intensity focused ultrasound [46-48, 52, 55, 57, 61-65, 68, 71, 72].

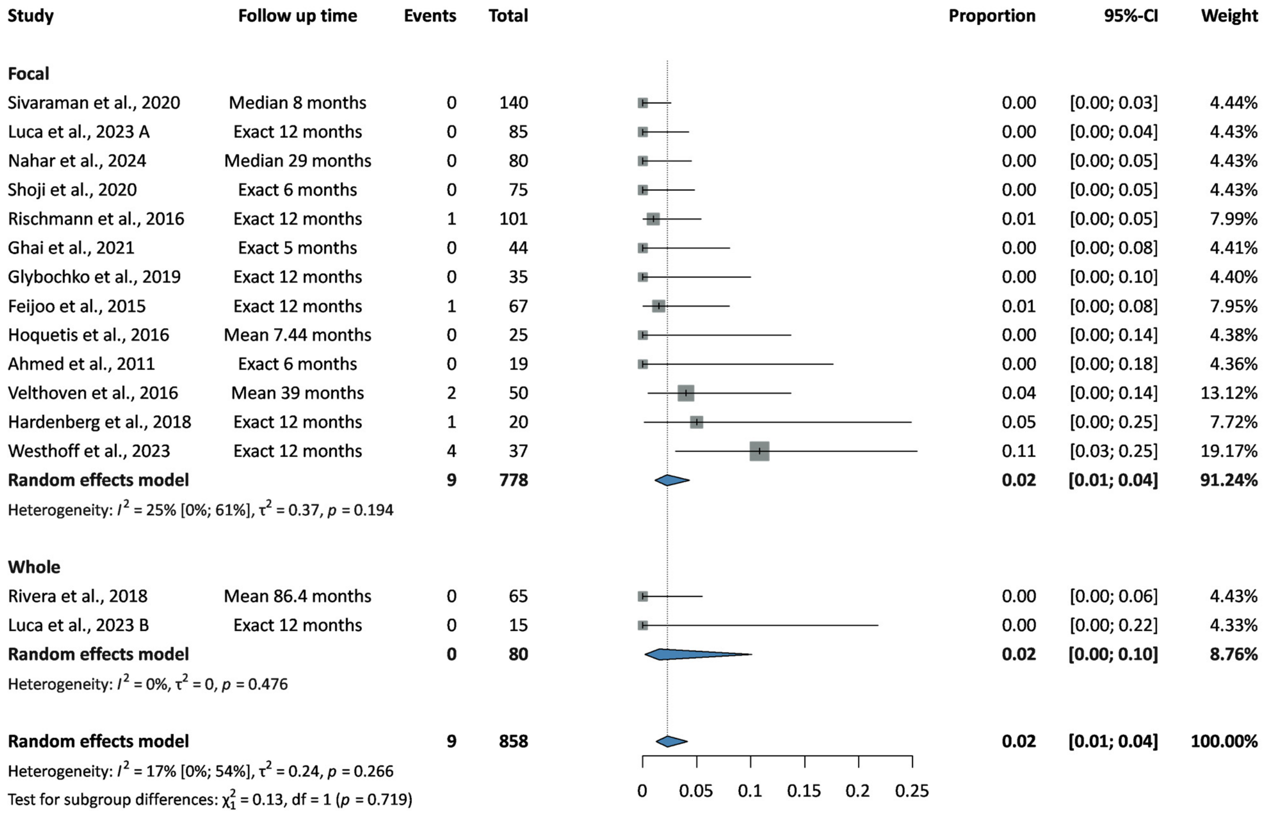

Figure S10. Summary forest plot of pooled concurrent in- and out-field recurrence rates regarding focal and whole gland high-intensity focused ultrasound [46-48, 55, 57, 61-65, 68, 71, 72, 85].

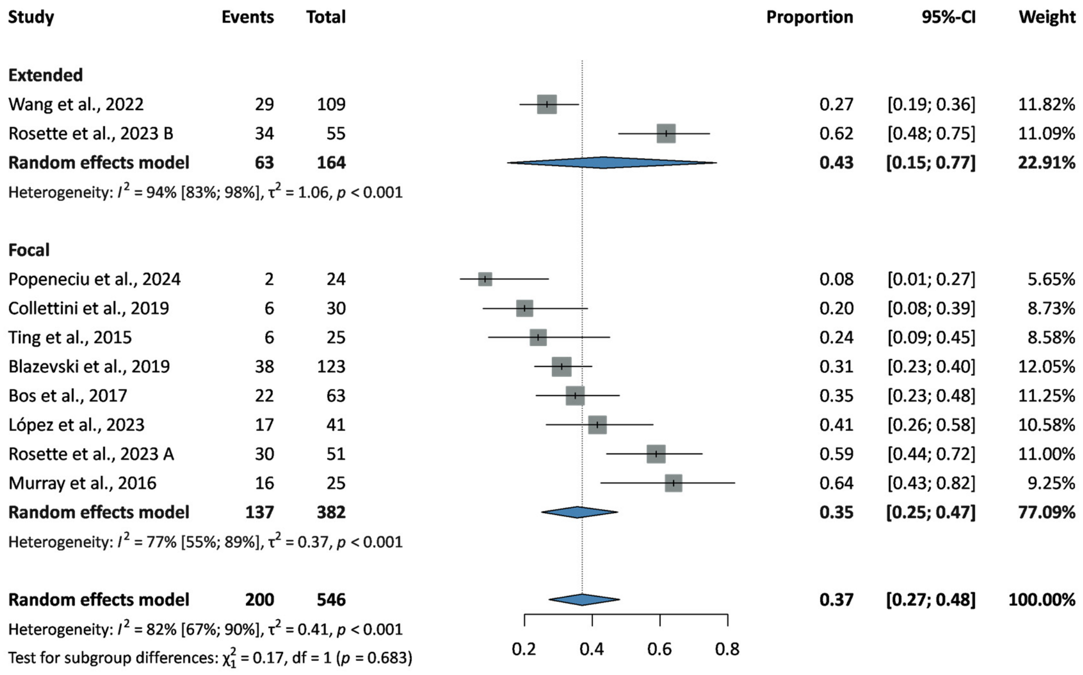

Figure S11. Summary forest plot of total complication rates regarding focal and extended irreversible electroporation [1-3, 6, 7, 10, 11, 13, 14].

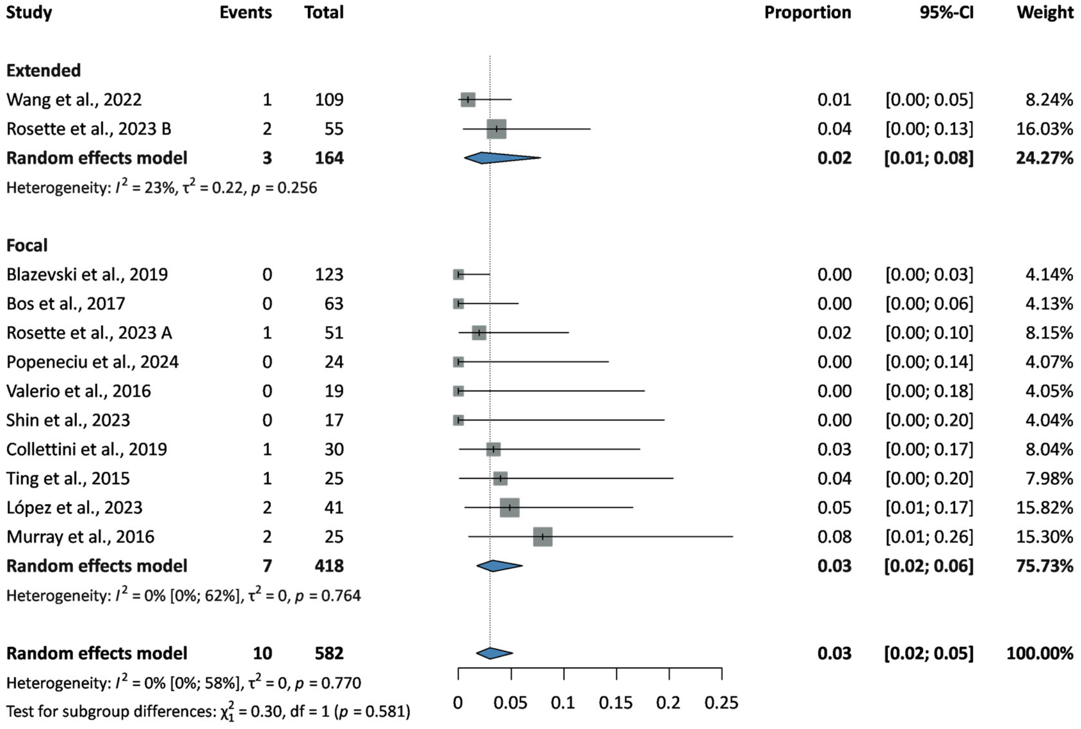

Figure S12. Summary forest plot of major complication rates regarding focal and extended irreversible electroporation [1-4, 6, 7, 10-14].

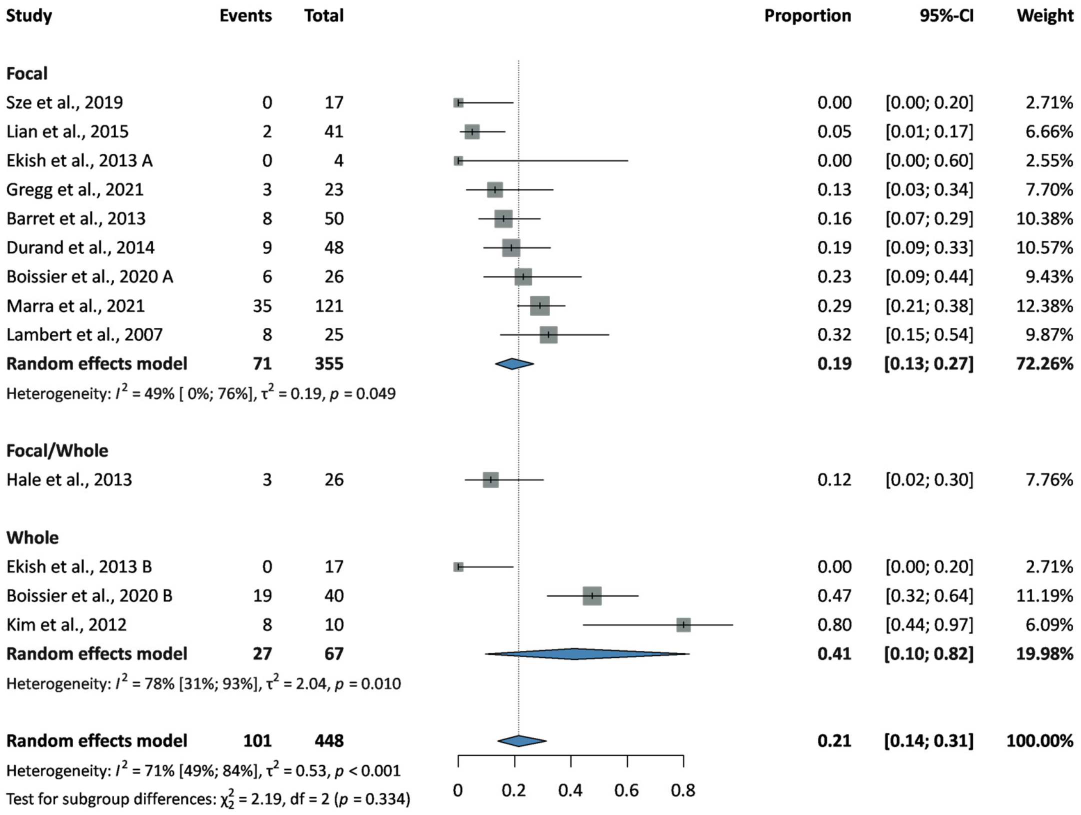

Figure S13. Summary forest plot of total complication rates regarding focal and whole gland cryoablation [17, 18, 22-24, 26, 27, 29-31, 36].

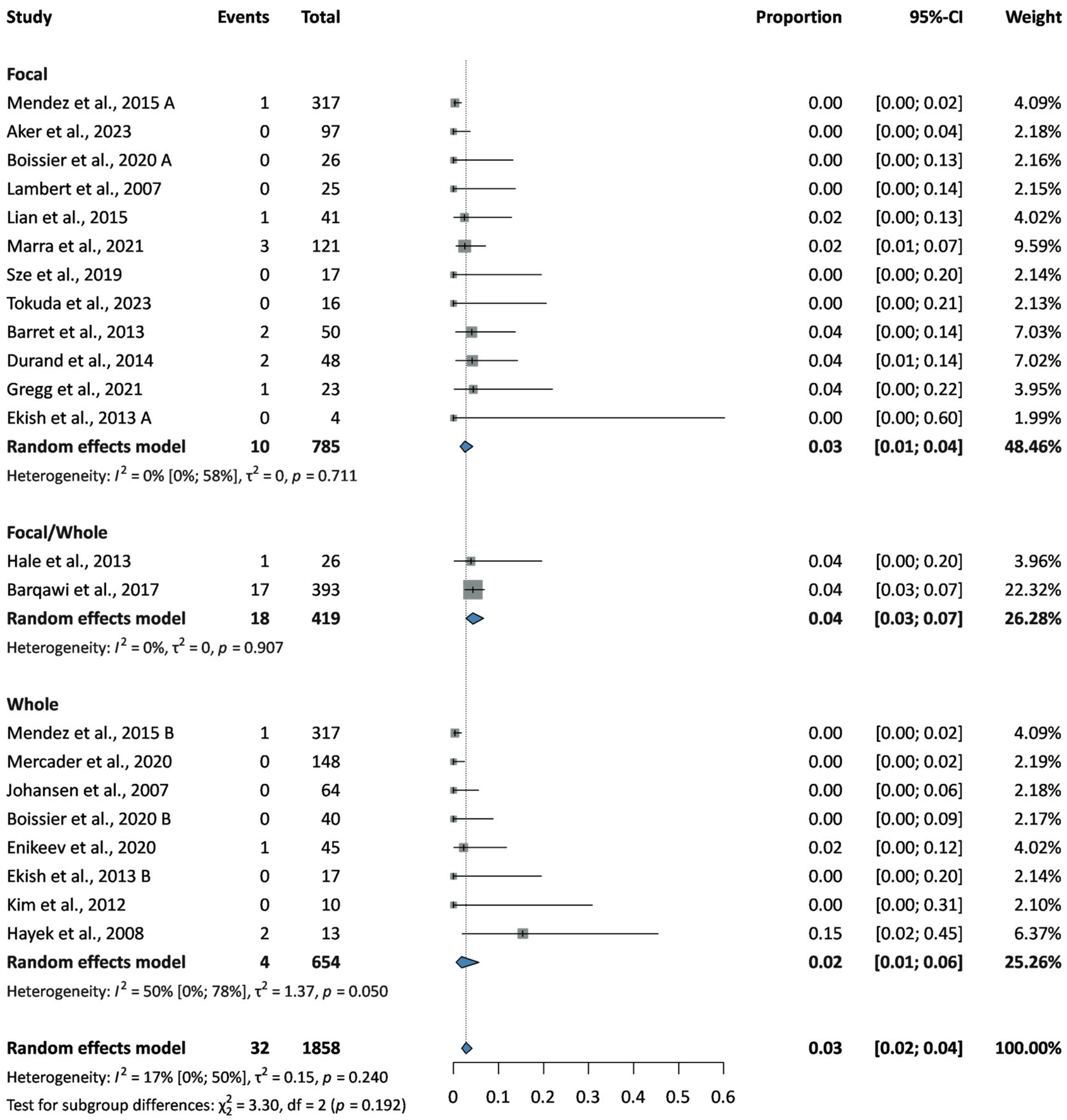

Figure S14. Summary forest plot of major complication rates regarding focal and whole gland cryoablation [15, 17, 18, 20-31, 35-37, 42].

Study	Events	Total	Proportion	95%-CI	Weight
**Focal**					
Glybochko et al., 2019	0	35	0.00	[0.00; 0.10]	1.02%
Arnouil et al., 2018	5	53	0.09	[0.03; 0.21]	4.70%
Sivaraman et al., 2020	16	140	0.11	[0.07; 0.18]	6.71%
Feijoo et al., 2015	10	67	0.15	[0.07; 0.26]	5.92%
Barret et al., 2013 H	5	21	0.24	[0.08; 0.47]	4.34%
Hoquetis et al., 2016	6	25	0.24	[0.09; 0.45]	4.71%
Fegoun et al., 2011	3	12	0.25	[0.05; 0.57]	3.25%
Dellabella et al., 2021	63	189	0.33	[0.27; 0.41]	7.73%
Westhoff et al., 2023	17	50	0.34	[0.21; 0.49]	6.37%
Ganzer et al., 2018	21	51	0.41	[0.28; 0.56]	6.51%
Ghai et al., 2021	24	44	0.55	[0.39; 0.70]	6.33%
Duwe et al., 2023	25	29	0.86	[0.68; 0.96]	4.13%
**Random effects model**	**195**	**716**	**0.28**	**[0.18; 0.40]**	**61.73%**
Heterogeneity: I^2 = 87% [79%; 92%], τ^2 = 0.75, p < 0.001					
**Whole**					
Capogrosso et al., 2018	19	84	0.23	[0.14; 0.33]	6.76%
Ploussard et al., 2024	471	1967	0.24	[0.22; 0.26]	8.31%
Blana et al., 2004	33	137	0.24	[0.17; 0.32]	7.35%
Crouzet et al., 2011	104	297	0.35	[0.30; 0.41]	7.97%
Poissonnier et al., 2007	98	227	0.43	[0.37; 0.50]	7.89%
**Random effects model**	**725**	**2712**	**0.30**	**[0.22; 0.38]**	**38.27%**
Heterogeneity: I^2 = 92% [84%; 96%], τ^2 = 0.17, p < 0.001					
**Random effects model**	**920**	**3428**	**0.29**	**[0.23; 0.36]**	**100.00%**
Heterogeneity: I^2 = 88% [83%; 92%], τ^2 = 0.28, p < 0.001					
Test for subgroup differences: χ^2_1 = 0.05, df = 1 (p = 0.828)					

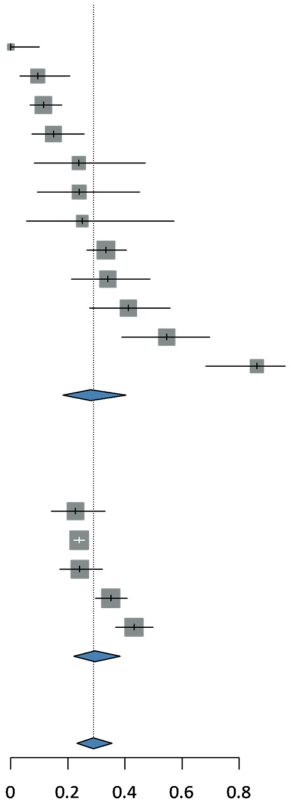

Figure S15. Summary forest plot of total complication rates regarding focal and whole gland high-intensity focused ultrasound [27, 46, 47, 50-57, 60, 61, 63, 66, 67, 84].

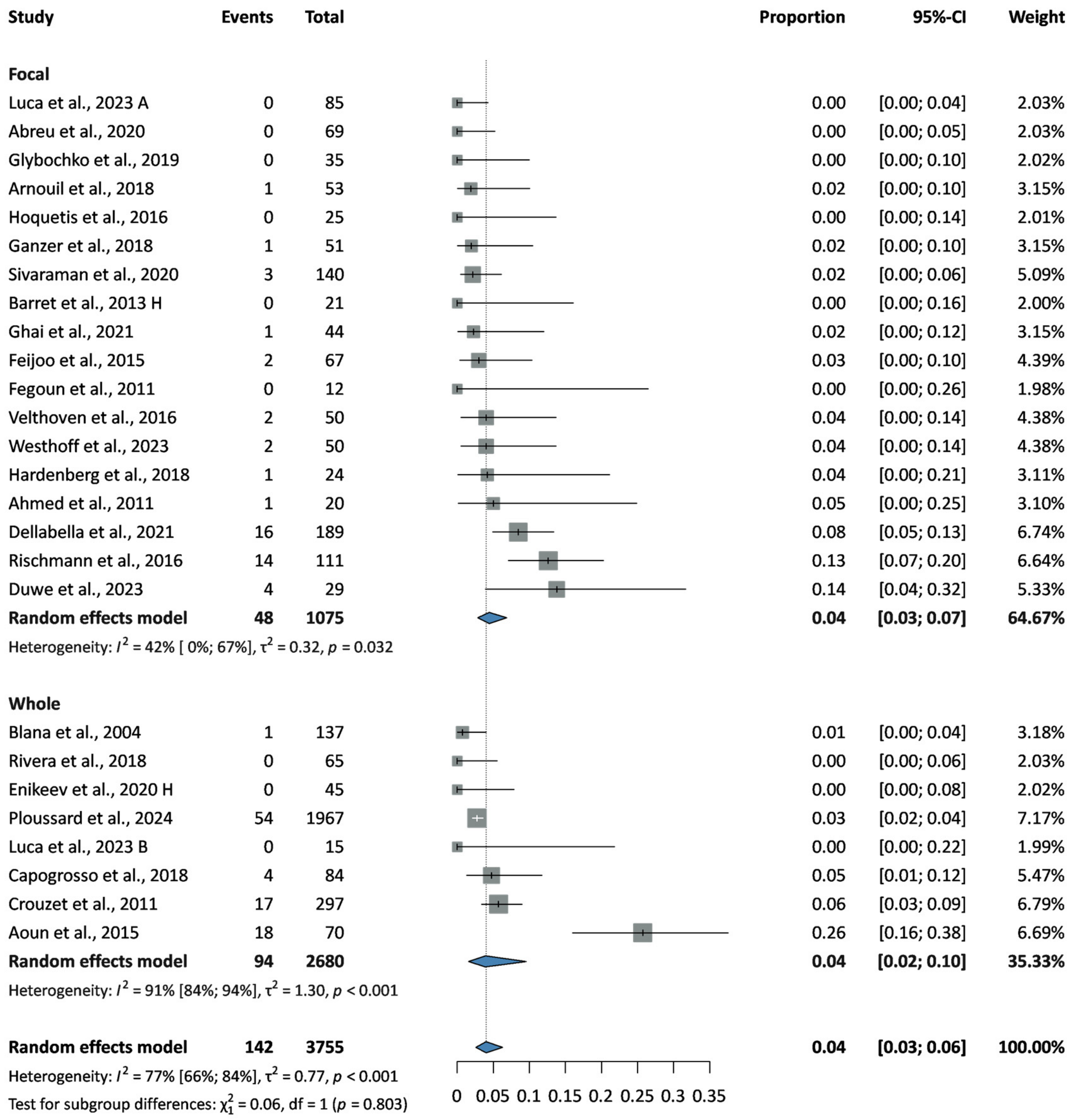

Figure S16. Summary forest plot of major complication rates regarding focal and whole gland high-intensity focused ultrasound [27, 28, 46-57, 61-68, 72, 74, 84].

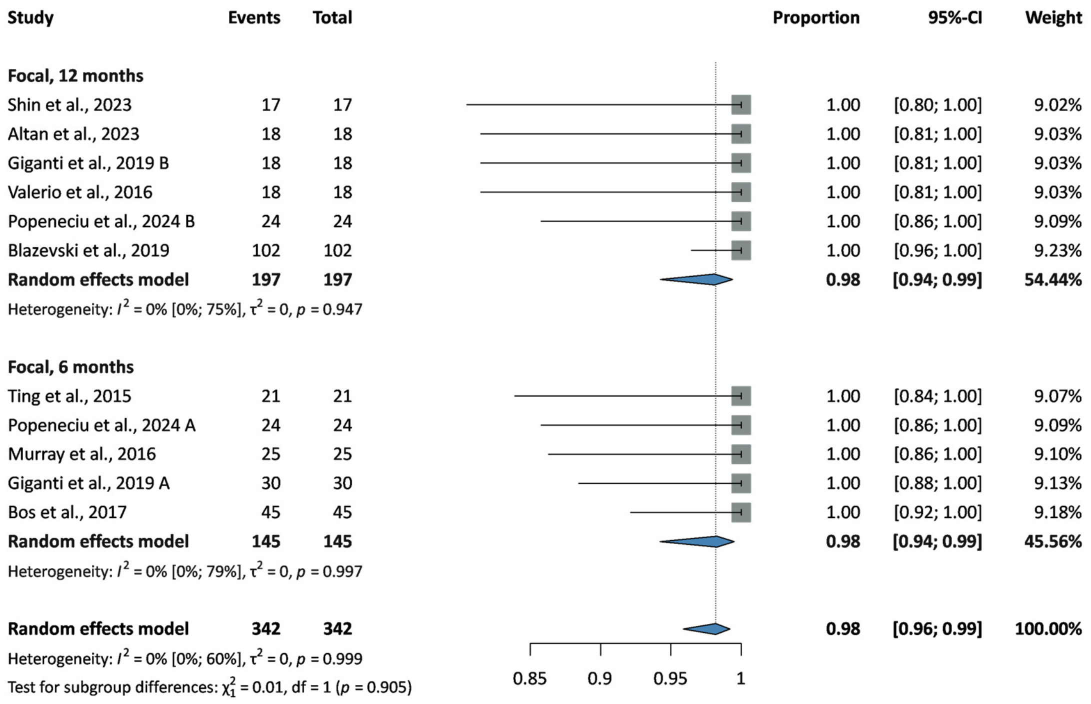

Figure S17. Summary forest plot of 12-month overall survival rates regarding focal irreversible electroporation [4-7, 9-13].

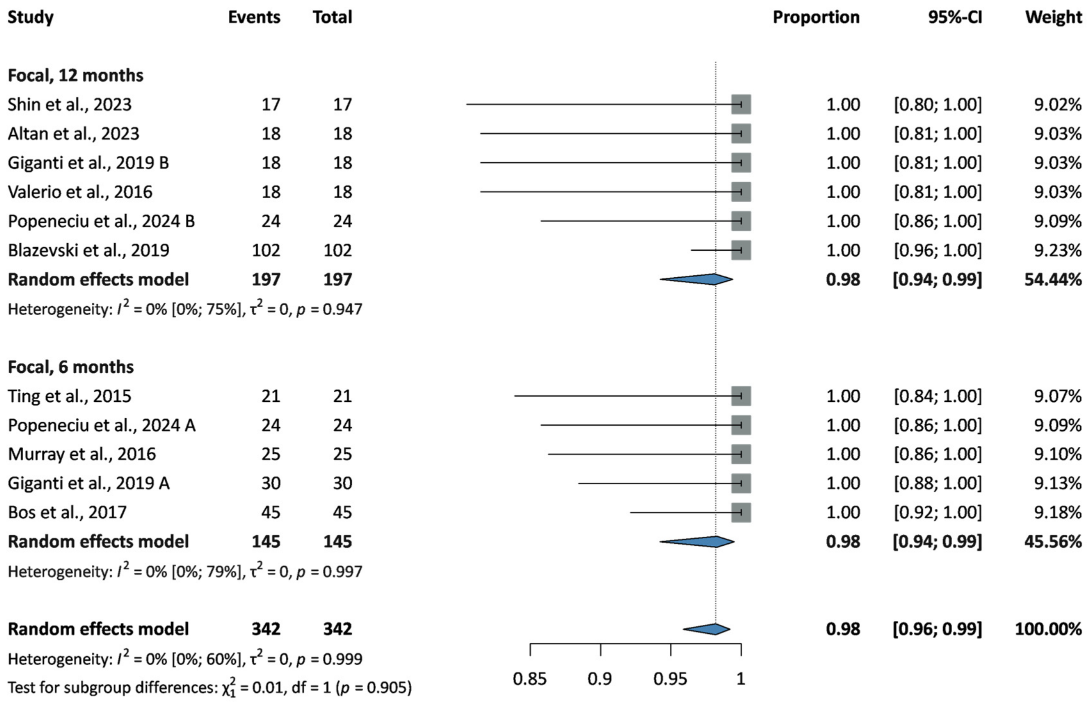

Figure S18. Summary forest plot of 12-month cancer-specific survival rates regarding focal irreversible electroporation [4-7, 9-13].

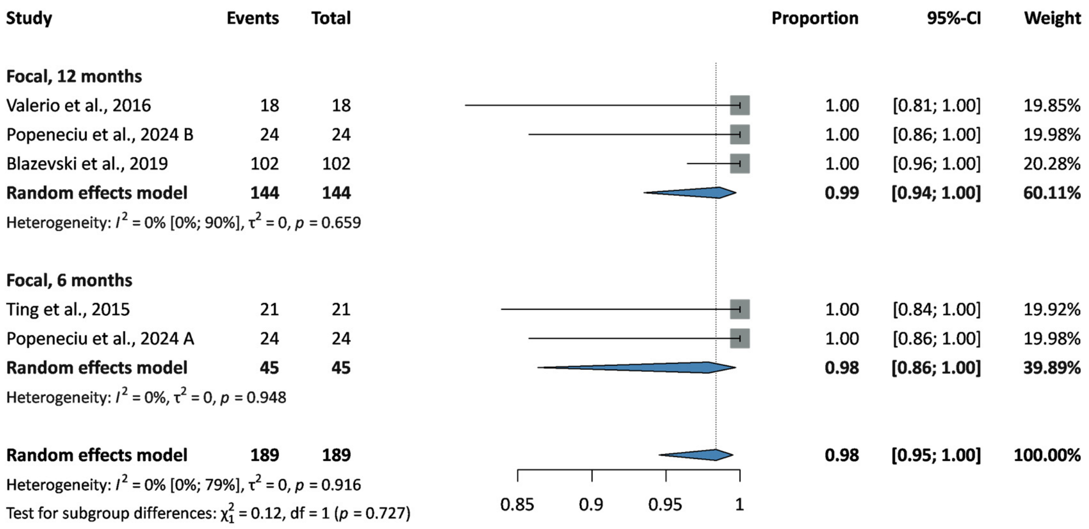

Figure S19. Summary forest plot of 12-month metastasis-free survival rates regarding focal irreversible electroporation [6, 7, 11, 12].

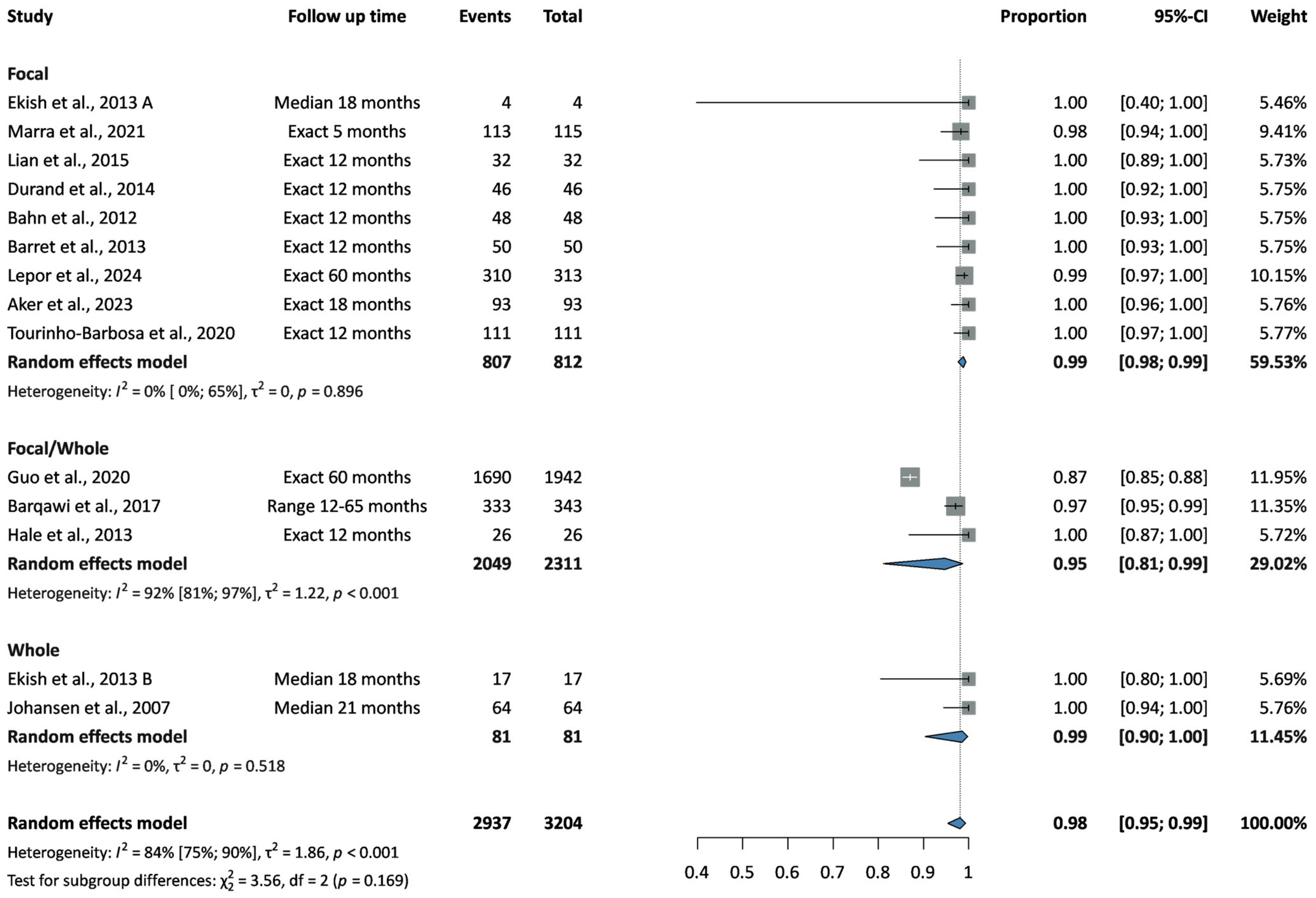

Figure S20. Summary forest plot of pooled overall survival rates regarding focal and whole gland cryoablation [19, 20, 24, 26, 27, 29, 30, 34-37, 44, 45].

Study	Follow up time	Events	Total	Proportion	95%-CI	Weight
**Focal**						
Ekish et al., 2013 A	Median 18 months	4	4	1.00	[0.40; 1.00]	2.60%
Lian et al., 2015	Exact 12 months	32	32	1.00	[0.89; 1.00]	2.84%
Durand et al., 2014	Exact 12 months	46	46	1.00	[0.92; 1.00]	2.85%
Bahn et al., 2012	Exact 12 months	48	48	1.00	[0.93; 1.00]	2.86%
Barret et al., 2013	Exact 12 months	50	50	1.00	[0.93; 1.00]	2.86%
Aker et al., 2023	Exact 18 months	93	93	1.00	[0.96; 1.00]	2.87%
Tourinho-Barbosa et al., 2020	Exact 12 months	111	111	1.00	[0.97; 1.00]	2.87%
Lepor et al., 2024	Exact 60 months	313	313	1.00	[0.99; 1.00]	2.88%
**Random effects model**		**697**	**697**	**0.99**	**[0.98; 1.00]**	**22.63%**
Heterogeneity: I^2 = 0% [0%; 68%], τ^2 = 0, p = 0.675						
**Focal/Whole**						
Hale et al., 2013	Exact 12 months	26	26	1.00	[0.87; 1.00]	2.83%
Barqawi et al., 2017	Range 12-65 months	341	343	0.99	[0.98; 1.00]	11.47%
Guo et al., 2020	Exact 60 months	1932	1942	0.99	[0.99; 1.00]	57.40%
**Random effects model**		**2299**	**2311**	**0.99**	**[0.99; 1.00]**	**71.71%**
Heterogeneity: I^2 = 0% [0%; 90%], τ^2 = 0, p = 0.674						
**Whole**						
Ekish et al., 2013 B	Median 18 months	17	17	1.00	[0.80; 1.00]	2.80%
Johansen et al., 2007	Median 21 months	64	64	1.00	[0.94; 1.00]	2.86%
**Random effects model**		**81**	**81**	**0.99**	**[0.90; 1.00]**	**5.67%**
Heterogeneity: I^2 = 0%, τ^2 = 0, p = 0.518						
**Random effects model**		**3077**	**3089**	**0.99**	**[0.99; 1.00]**	**100.00%**
Heterogeneity: I^2 = 0% [0%; 57%], τ^2 = 0, p = 0.819						
Test for subgroup differences: χ^2_2 = 1.47, df = 2 (p = 0.480)						

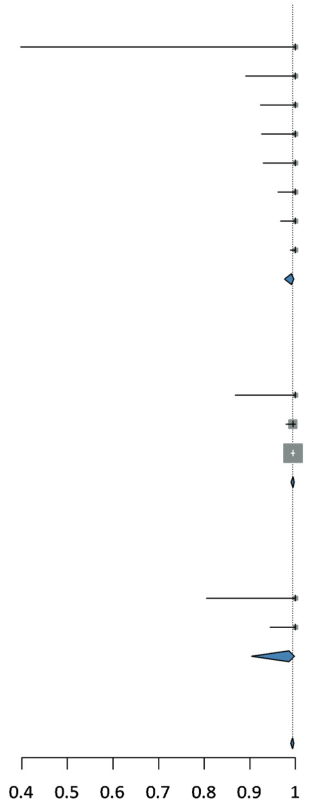

Figure S21. Summary forest plot of pooled cancer-specific survival rates regarding focal and whole gland cryoablation [19, 20, 24, 27, 29, 30, 34-37, 44, 45].

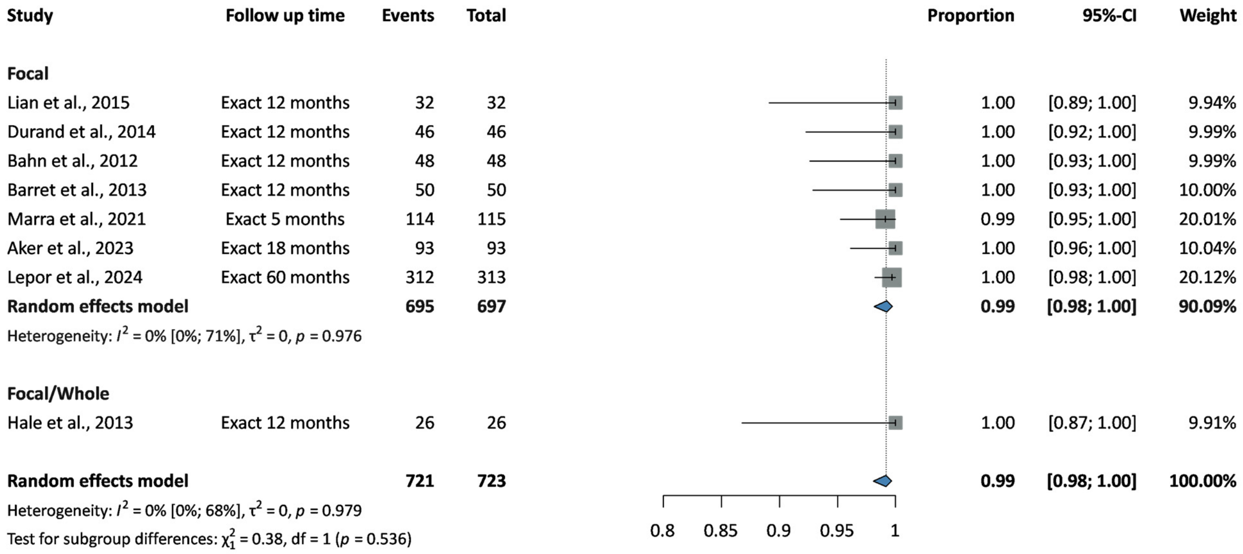

Figure S22. Summary forest plot of pooled metastasis-free survival rates regarding focal and whole gland cryoablation [19, 24, 26, 27, 29, 30, 35, 45].

Study	Follow up time	Events	Total	Proportion	95%-CI	Weight
**Focal**						
Fegoun et al., 2011 A	Exact 60 months	10	12	0.83	[0.52; 0.98]	5.03%
Velthoven et al., 2016	Exact 60 months	44	50	0.88	[0.76; 0.95]	7.29%
Westhoff et al., 2023	Median 42 months	36	37	0.97	[0.86; 1.00]	3.80%
Ahmed et al., 2011	Exact 12 months	19	19	1.00	[0.82; 1.00]	2.40%
Hardenberg et al., 2018	Mean 18.4 months	20	20	1.00	[0.83; 1.00]	2.40%
Barret et al., 2013	Median 9 months	21	21	1.00	[0.84; 1.00]	2.40%
Rischmann et al., 2016	Mean 30.4 months	99	101	0.98	[0.93; 1.00]	5.39%
Hoquetis et al., 2016	Mean 25.1 months	25	25	1.00	[0.86; 1.00]	2.41%
Dellabella et al., 2021	Median 28 months	174	177	0.98	[0.95; 1.00]	6.26%
Ghai et al., 2021	Exact 5 months	44	44	1.00	[0.92; 1.00]	2.42%
Ganzer et al., 2018	Exact 12 months	49	49	1.00	[0.93; 1.00]	2.42%
Feijoo et al., 2015	Median 12	67	67	1.00	[0.95; 1.00]	2.43%
Shoji et al., 2020	Median 21 months	75	75	1.00	[0.95; 1.00]	2.43%
Tourinho-Barbosa et al., 2020 A	Exact 12 months	174	174	1.00	[0.98; 1.00]	2.44%
Reddy et al., 2022 A	Exact 12 months	916	916	1.00	[1.00; 1.00]	2.44%
**Random effects model**		**1773**	**1787**	**0.98**	**[0.96; 0.99]**	**51.95%**
Heterogeneity: $I^2 = 58\%$ [25%; 76%], $\tau^2 = 1.26$, $p = 0.003$						
**Whole**						
Aoun et al., 2015 A	Exact 60 months	62	70	0.89	[0.79; 0.95]	7.70%
Ripert et al., 2010	Mean 45.4 months	47	53	0.89	[0.77; 0.96]	7.30%
Rosenhammer et al., 2019 A	Exact 60 months	366	402	0.91	[0.88; 0.94]	8.83%
Capogrosso et al., 2018	Exact 60 months	23	24	0.96	[0.79; 1.00]	3.76%
Poissonnier et al., 2007	Mean 27.5 months	220	227	0.97	[0.94; 0.99]	7.64%
Ploussard et al., 2024	Exact 30 months	1876	1930	0.97	[0.96; 0.98]	8.97%
Pinthus et al., 2012	Median 24 months	401	402	1.00	[0.99; 1.00]	3.85%
**Random effects model**		**2995**	**3108**	**0.95**	**[0.91; 0.98]**	**48.05%**
Heterogeneity: $I^2 = 89\%$ [79%; 94%], $\tau^2 = 0.67$, $p < 0.001$						
**Random effects model**		**4768**	**4895**	**0.97**	**[0.95; 0.98]**	**100.00%**
Heterogeneity: $I^2 = 76\%$ [64%; 84%], $\tau^2 = 0.72$, $p < 0.001$						
Test for subgroup differences: $\chi^2_1 = 2.73$, df = 1 ($p = 0.099$)						

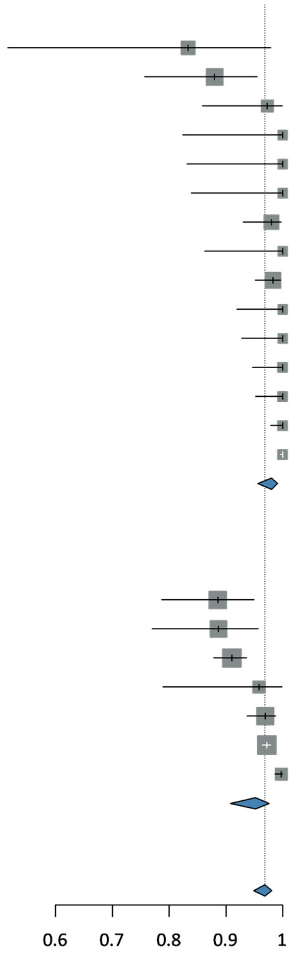

Figure S23. Summary forest plot of pooled overall survival rates regarding focal and whole gland high-intensity focused ultrasound [27, 44, 46, 48, 49, 51, 52, 54-57, 59-62, 64, 65, 70, 71, 77, 82, 84].

Study	Follow up time	Events	Total	Proportion	95%-CI	Weight
**Focal**						
Fegoun et al., 2011 A	Exact 60 months	12	12	1.00	[0.74; 1.00]	3.66%
Ahmed et al., 2011	Exact 12 months	19	19	1.00	[0.82; 1.00]	3.69%
Hardenberg et al., 2018	Mean 18.4 months	20	20	1.00	[0.83; 1.00]	3.69%
Barret et al., 2013	Median 9 months	21	21	1.00	[0.84; 1.00]	3.69%
Hoquetis et al., 2016	Mean 25.1 months	25	25	1.00	[0.86; 1.00]	3.70%
Westhoff et al., 2023	Median 42 months	37	37	1.00	[0.91; 1.00]	3.71%
Ghai et al., 2021	Exact 5 months	44	44	1.00	[0.92; 1.00]	3.71%
Ganzer et al., 2018	Exact 12 months	49	49	1.00	[0.93; 1.00]	3.72%
Velthoven et al., 2016	Exact 60 months	50	50	1.00	[0.93; 1.00]	3.72%
Feijoo et al., 2015	Median 12	67	67	1.00	[0.95; 1.00]	3.72%
Shoji et al., 2020	Median 21 months	75	75	1.00	[0.95; 1.00]	3.72%
Rischmann et al., 2016	Mean 30.4 months	101	101	1.00	[0.96; 1.00]	3.73%
Tourinho-Barbosa et al., 2020 A	Exact 12 months	174	174	1.00	[0.98; 1.00]	3.73%
Dellabella et al., 2021	Median 28 months	177	177	1.00	[0.98; 1.00]	3.73%
Reddy et al., 2022 A	Exact 12 months	916	916	1.00	[1.00; 1.00]	3.74%
**Random effects model**		**1787**	**1787**	**0.99**	**[0.98; 1.00]**	**55.65%**
Heterogeneity: I^2 = 0% [0%; 54%], τ^2 = 0, p = 0.865						
**Whole**						
Aoun et al., 2015 A	Exact 60 months	62	70	0.89	[0.79; 0.95]	7.39%
Capogrosso et al., 2018	Exact 60 months	24	24	1.00	[0.86; 1.00]	3.70%
Rosenhammer et al., 2019 A	Exact 60 months	397	402	0.99	[0.97; 1.00]	7.16%
Ripert et al., 2010	Mean 45.4 months	53	53	1.00	[0.93; 1.00]	3.72%
Misraï et al.,2008	Mean 46.8 months	115	115	1.00	[0.97; 1.00]	3.73%
Mearini et al., 2014 A	Exact 12 months	127	127	1.00	[0.97; 1.00]	3.73%
Crouzet et al., 2011	Exact 60 months	175	175	1.00	[0.98; 1.00]	3.73%
Poissonnier et al., 2007	Mean 27.5 months	227	227	1.00	[0.98; 1.00]	3.73%
Pinthus et al., 2012	Median 24 months	402	402	1.00	[0.99; 1.00]	3.73%
Ploussard et al., 2024	Exact 30 months	1930	1930	1.00	[1.00; 1.00]	3.74%
**Random effects model**		**3512**	**3525**	**0.99**	**[0.98; 1.00]**	**44.35%**
Heterogeneity: I^2 = 80% [64%; 89%], τ^2 = 3.24, p < 0.001						
**Random effects model**		**5299**	**5312**	**0.99**	**[0.98; 1.00]**	**100.00%**
Heterogeneity: I^2 = 59% [37%; 74%], τ^2 = 1.76, p < 0.001						
Test for subgroup differences: χ^2_1 = 0.21, df = 1 (p = 0.646)						

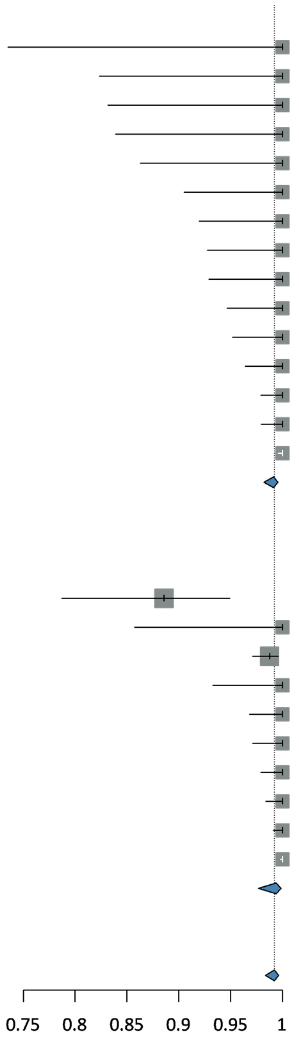

Figure S24. Summary forest plot of pooled cancer-specific survival rates regarding focal and whole gland high-intensity focused ultrasound [27, 44, 46, 48, 49, 51, 52, 54-57, 59-62, 64, 65, 67, 69, 71, 77, 80, 82, 84].

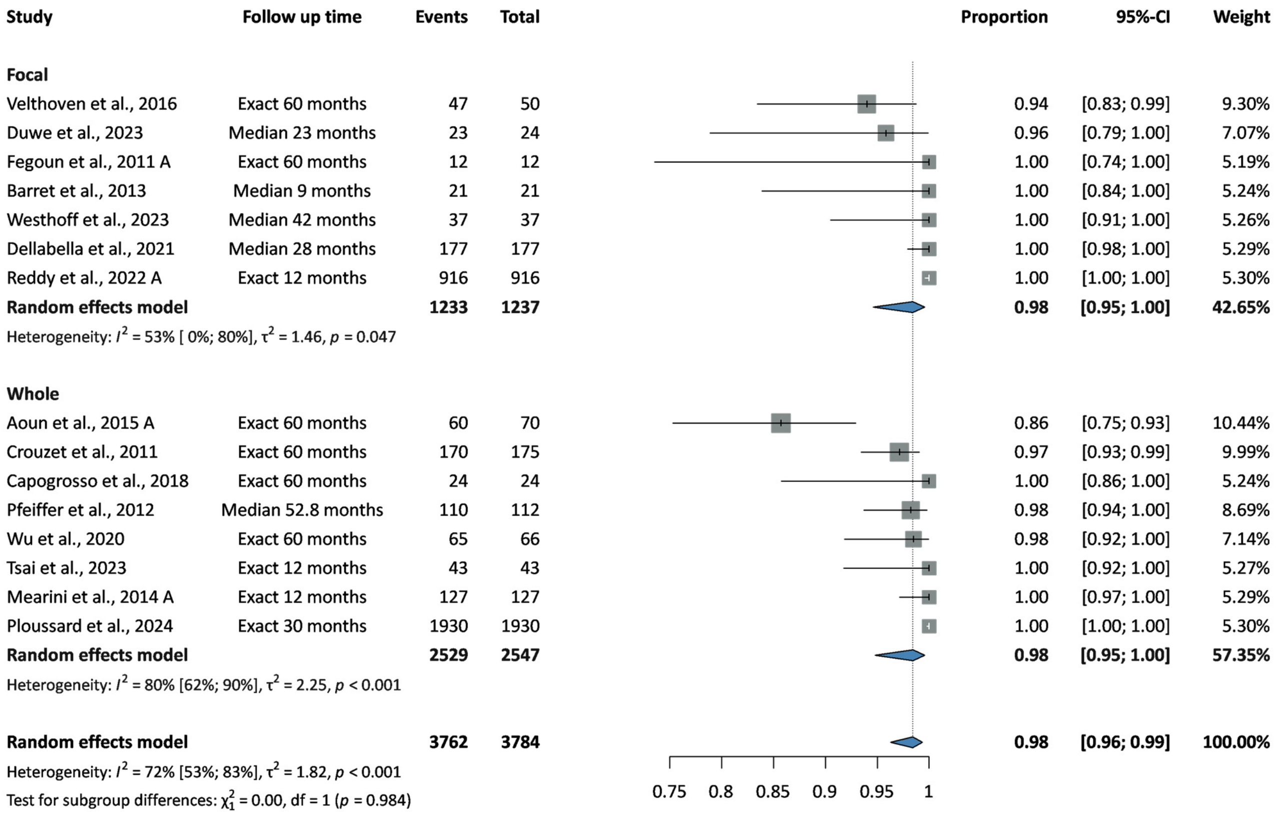

Figure S25. Summary forest plot of pooled metastasis-free survival rates regarding focal and whole gland high-intensity focused ultrasound [27, 46, 49, 51-54, 64, 67, 73, 77, 80, 81, 83, 84].

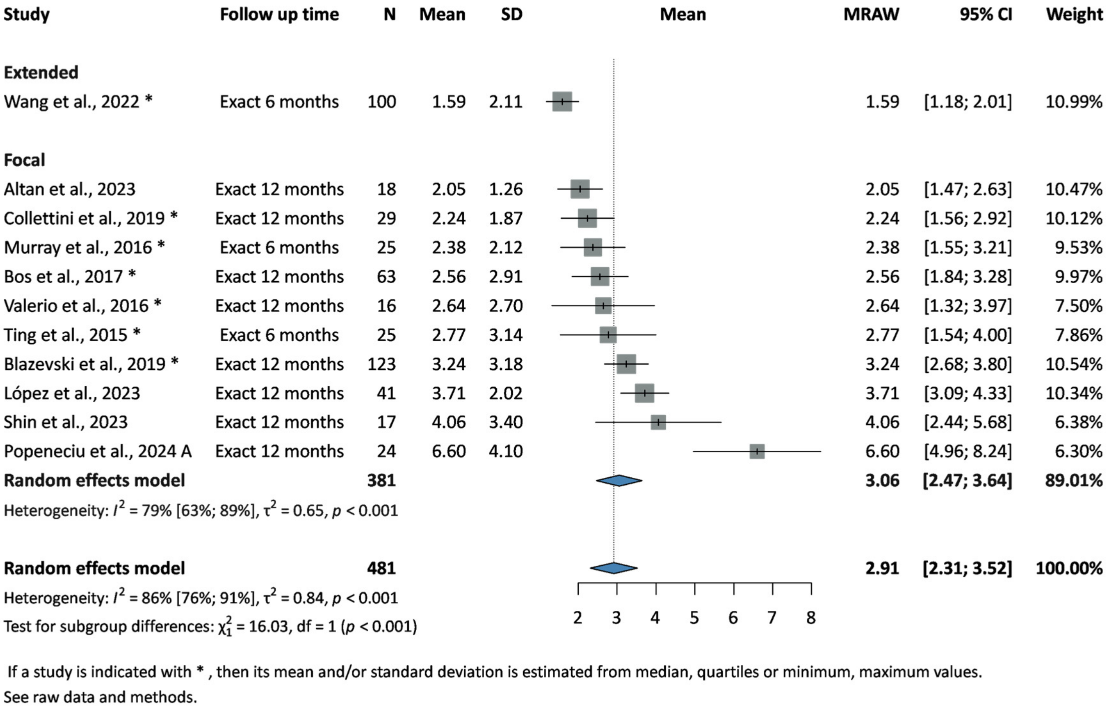

Figure S26. Summary forest plot of mean postoperative PSA levels regarding focal and extended irreversible electroporation [2-4, 6, 7, 9-14].

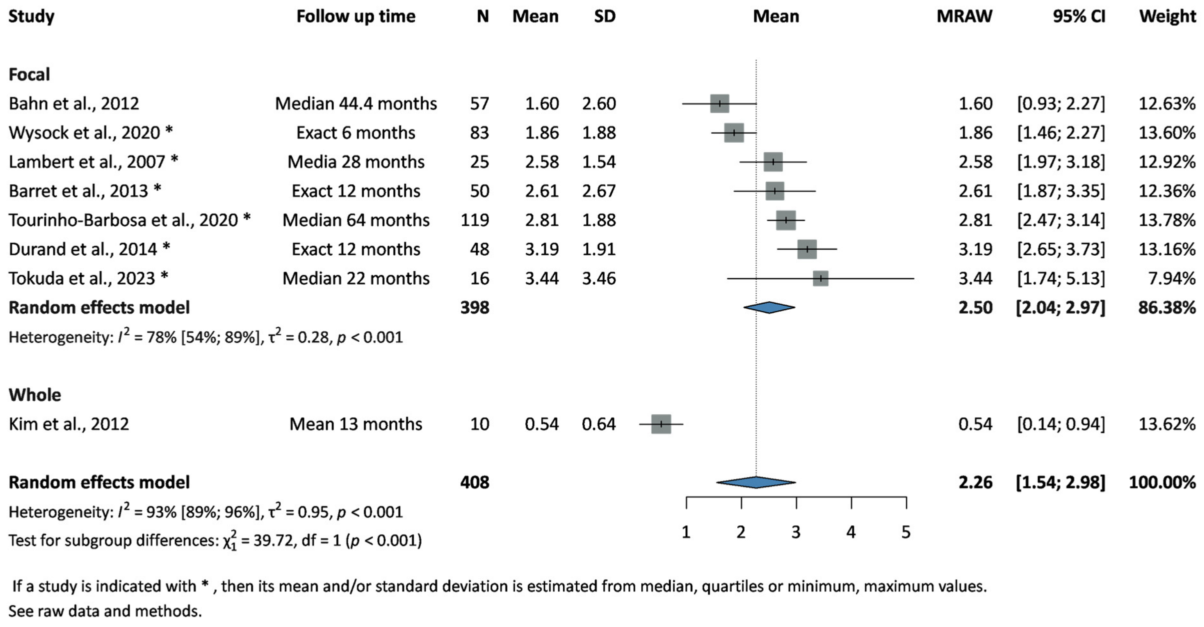

Figure S27. Summary forest plot of mean postoperative PSA levels regarding focal and whole gland cryoablation [15, 16, 18, 19, 23, 27, 29, 44].

Study	Follow up time	N	Mean	SD	MRAW	95% CI	Weight
**Focal**							
Ahmed et al., 2011 *	Exact 12 months	20	1.36	1.35	1.36	[0.77; 1.96]	6.27%
Fegoun et al., 2011 A	Median 127 months	12	1.55	0.82	1.55	[1.09; 2.01]	6.44%
Duwe et al., 2023 *	Exact 12 months	20	2.47	2.64	2.47	[1.31; 3.63]	5.24%
Rischmann et al., 2016	Exact 12 months	111	2.50	2.10	2.50	[2.11; 2.89]	6.53%
Sivaraman et al., 2020	Exact 3 months	88	2.50	3.30	2.50	[1.81; 3.19]	6.12%
Ganzer et al., 2018 *	Exact 12 months	51	2.62	0.99	2.62	[2.35; 2.89]	6.63%
Tourinho-Barbosa et al., 2020 A *	Median 37 months	190	2.85	2.32	2.85	[2.52; 3.17]	6.59%
Ghai et al., 2021 *	Exact 5 months	44	3.00	3.29	3.00	[2.03; 3.97]	5.61%
Barret et al., 2013 *	Exact 12 months	21	3.28	1.51	3.28	[2.63; 3.92]	6.19%
Westhoff et al., 2023 *	Exact 12 months	50	3.42	2.51	3.42	[2.72; 4.12]	6.11%
Glybochko et al., 2019 *	Exact 12 months	35	3.51	2.70	3.51	[2.61; 4.40]	5.76%
Feijoo et al., 2015 *	Exact 12 months	67	3.61	2.65	3.61	[2.97; 4.24]	6.21%
Luca et al., 2023 A	Exact 12 months	85	4.09	2.62	4.09	[3.53; 4.65]	6.32%
**Random effects model**		**794**			**2.81**	**[2.41; 3.21]**	**80.04%**
Heterogeneity: I^2 = 86% [78%; 91%], τ^2 = 0.44, p < 0.001							
**Whole**							
Luca et al., 2023 B	Exact 12 months	15	0.46	0.28	0.46	[0.32; 0.60]	6.71%
Blana et al., 2004 *	Median 22 months	137	0.69	2.32	0.69	[0.30; 1.08]	6.53%
Poissonnier et al., 2007	Median 20.5 months	227	0.89	0.83	0.89	[0.78; 1.00]	6.72%
**Random effects model**		**379**			**0.68**	**[0.35; 1.01]**	**19.96%**
Heterogeneity: I^2 = 91% [77%; 97%], τ^2 = 0.07, p < 0.001							
**Random effects model**		**1173**			**2.39**	**[1.83; 2.96]**	**100.00%**
Heterogeneity: I^2 = 98% [97%; 98%], τ^2 = 1.23, p < 0.001							
Test for subgroup differences: χ^2_1 = 63.79, df = 1 (p < 0.001)							

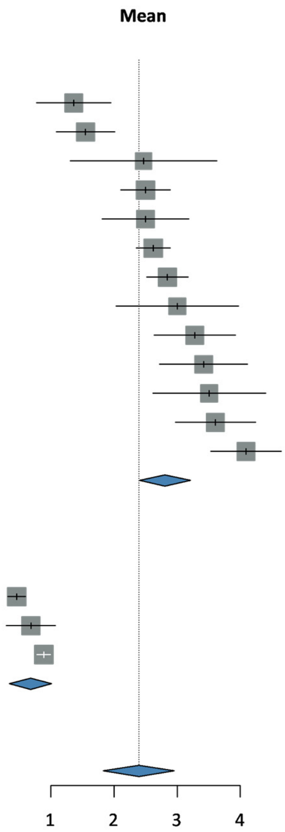

If a study is indicated with * , then its mean and/or standard deviation is estimated from median, quartiles or minimum, maximum values.
See raw data and methods.

Figure S28. Summary forest plot of mean postoperative PSA levels regarding focal and whole gland high-intensity focused ultrasound [27, 44, 46-48, 50, 53-56, 60-63, 68].

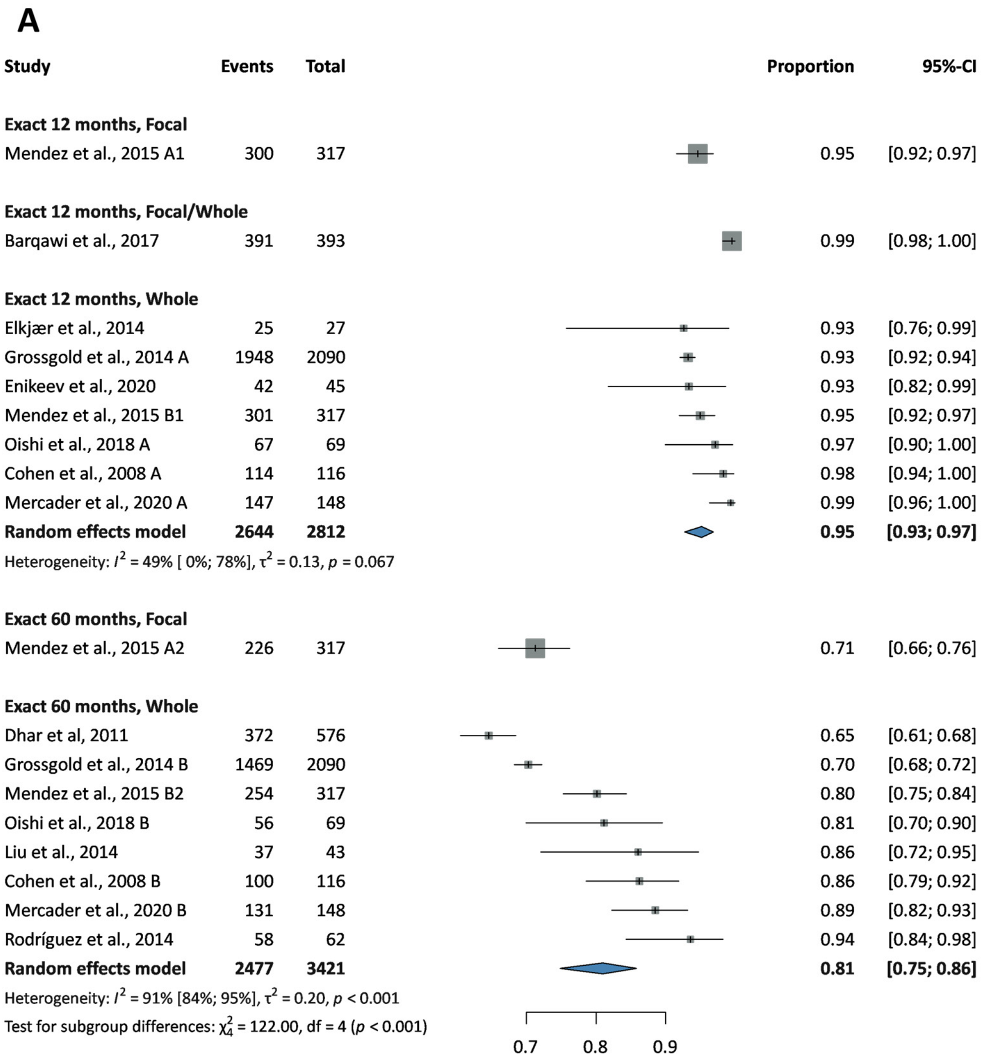
A
Study
Events
Total
Proportion
95%-CI
Exact 12 months, Focal
Mendez et al., 2015 A1 300 317 0.95 [0.92; 0.97]
Exact 12 months, Focal/Whole
Barqawi et al., 2017 391 393 0.99 [0.98; 1.00]
Exact 12 months, Whole
Elkjær et al., 2014 25 27 0.93 [0.76; 0.99]
Grossgold et al., 2014 A 1948 2090 0.93 [0.92; 0.94]
Enikeev et al., 2020 42 45 0.93 [0.82; 0.99]
Mendez et al., 2015 B1 301 317 0.95 [0.92; 0.97]
Oishi et al., 2018 A 67 69 0.97 [0.90; 1.00]
Cohen et al., 2008 A 114 116 0.98 [0.94; 1.00]
Mercader et al., 2020 A 147 148 0.99 [0.96; 1.00]
Random effects model 2644 2812 0.95 [0.93; 0.97]
Heterogeneity: I^2 = 49% [0%; 78%], τ^2 = 0.13, p = 0.067
Exact 60 months, Focal
Mendez et al., 2015 A2 226 317 0.71 [0.66; 0.76]
Exact 60 months, Whole
Dhar et al, 2011 372 576 0.65 [0.61; 0.68]
Grossgold et al., 2014 B 1469 2090 0.70 [0.68; 0.72]
Mendez et al., 2015 B2 254 317 0.80 [0.75; 0.84]
Oishi et al., 2018 B 56 69 0.81 [0.70; 0.90]
Liu et al., 2014 37 43 0.86 [0.72; 0.95]
Cohen et al., 2008 B 100 116 0.86 [0.79; 0.92]
Mercader et al., 2020 B 131 148 0.89 [0.82; 0.93]
Rodríguez et al., 2014 58 62 0.94 [0.84; 0.98]
Random effects model 2477 3421 0.81 [0.75; 0.86]
Heterogeneity: I^2 = 91% [84%; 95%], τ^2 = 0.20, p < 0.001
Test for subgroup differences: χ^2_4 = 122.00, df = 4 (p < 0.001)
0.7
0.8
0.9

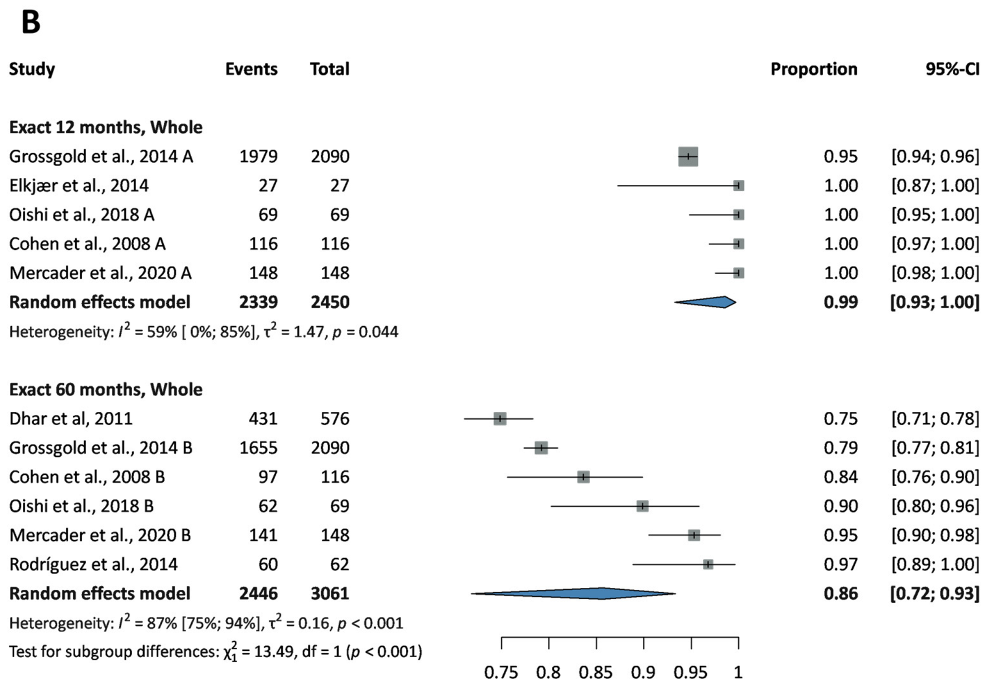
B
Study
Events
Total
Proportion
95%-CI
Exact 12 months, Whole
Grossgold et al., 2014 A 1979 2090 0.95 [0.94; 0.96]
Elkjær et al., 2014 27 27 1.00 [0.87; 1.00]
Oishi et al., 2018 A 69 69 1.00 [0.95; 1.00]
Cohen et al., 2008 A 116 116 1.00 [0.97; 1.00]
Mercader et al., 2020 A 148 148 1.00 [0.98; 1.00]
Random effects model 2339 2450 0.99 [0.93; 1.00]
Heterogeneity: I^2 = 59% [0%; 85%], τ^2 = 1.47, p = 0.044
Exact 60 months, Whole
Dhar et al, 2011 431 576 0.75 [0.71; 0.78]
Grossgold et al., 2014 B 1655 2090 0.79 [0.77; 0.81]
Cohen et al., 2008 B 97 116 0.84 [0.76; 0.90]
Oishi et al., 2018 B 62 69 0.90 [0.80; 0.96]
Mercader et al., 2020 B 141 148 0.95 [0.90; 0.98]
Rodríguez et al., 2014 60 62 0.97 [0.89; 1.00]
Random effects model 2446 3061 0.86 [0.72; 0.93]
Heterogeneity: I^2 = 87% [75%; 94%], τ^2 = 0.16, p < 0.001
Test for subgroup differences: χ^2_1 = 13.49, df = 1 (p < 0.001)
0.75
0.8
0.85
0.9
0.95
1

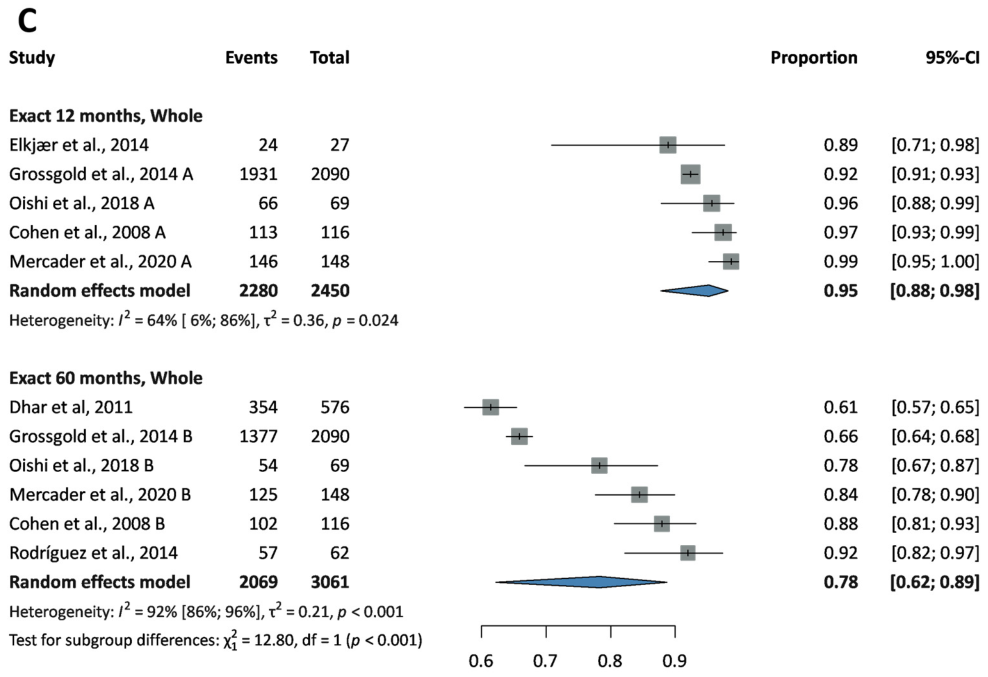

Figure S29. Summary forest plot of 12- and 60-month BFRS regarding focal and whole gland cryoablation (Phoenix criteria). (A) BFRS among patients with low- and intermediate-risk prostate cancer; (B) BFRS among patients with low-risk prostate cancer; (C) BFRS among patients with intermediate-risk prostate cancer [20, 25, 28, 32, 33, 38-43].

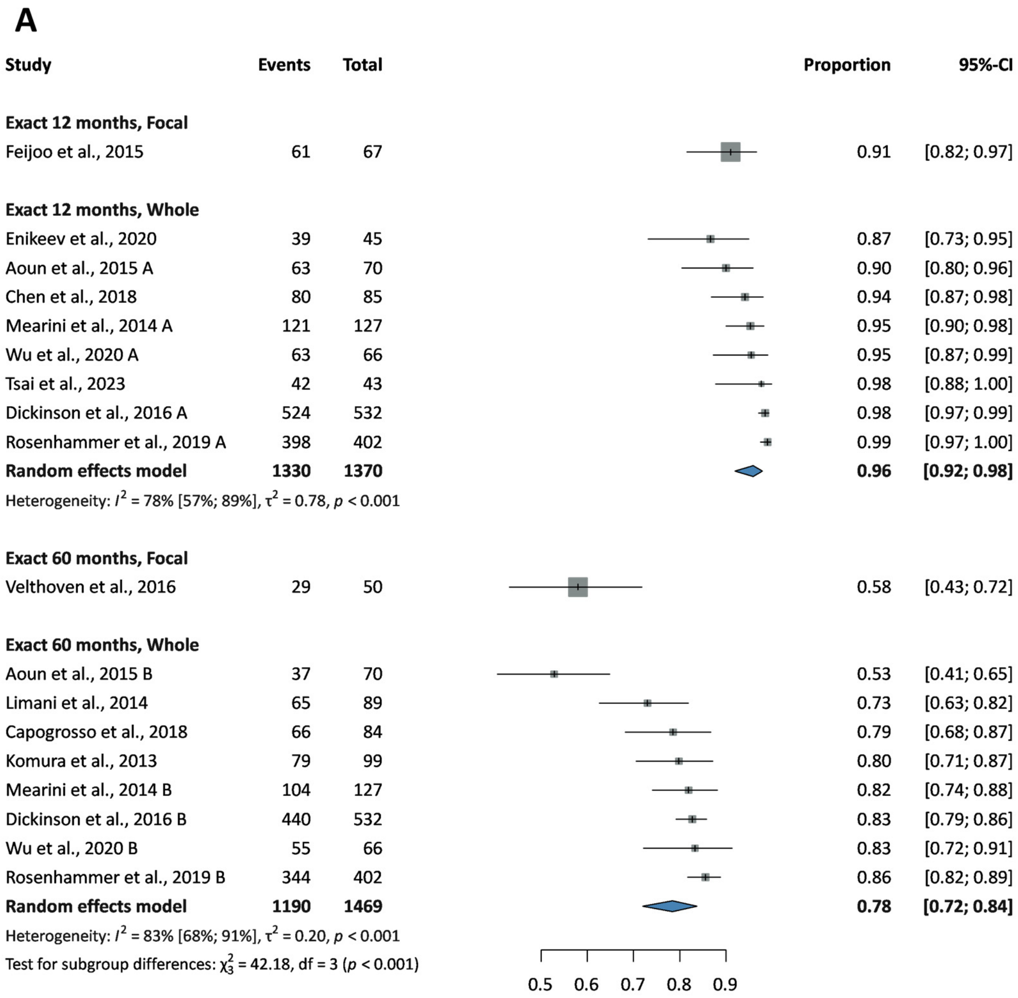
A
Study
Events
Total
Proportion
95%-CI
Exact 12 months, Focal
Feijoo et al., 2015 61 67 0.91 [0.82; 0.97]
Exact 12 months, Whole
Enikeev et al., 2020 39 45 0.87 [0.73; 0.95]
Aoun et al., 2015 A 63 70 0.90 [0.80; 0.96]
Chen et al., 2018 80 85 0.94 [0.87; 0.98]
Mearini et al., 2014 A 121 127 0.95 [0.90; 0.98]
Wu et al., 2020 A 63 66 0.95 [0.87; 0.99]
Tsai et al., 2023 42 43 0.98 [0.88; 1.00]
Dickinson et al., 2016 A 524 532 0.98 [0.97; 0.99]
Rosenhammer et al., 2019 A 398 402 0.99 [0.97; 1.00]
Random effects model 1330 1370 0.96 [0.92; 0.98]
Heterogeneity: I^2 = 78% [57%; 89%], τ^2 = 0.78, p < 0.001
Exact 60 months, Focal
Velthoven et al., 2016 29 50 0.58 [0.43; 0.72]
Exact 60 months, Whole
Aoun et al., 2015 B 37 70 0.53 [0.41; 0.65]
Limani et al., 2014 65 89 0.73 [0.63; 0.82]
Capogrosso et al., 2018 66 84 0.79 [0.68; 0.87]
Komura et al., 2013 79 99 0.80 [0.71; 0.87]
Mearini et al., 2014 B 104 127 0.82 [0.74; 0.88]
Dickinson et al., 2016 B 440 532 0.83 [0.79; 0.86]
Wu et al., 2020 B 55 66 0.83 [0.72; 0.91]
Rosenhammer et al., 2019 B 344 402 0.86 [0.82; 0.89]
Random effects model 1190 1469 0.78 [0.72; 0.84]
Heterogeneity: I^2 = 83% [68%; 91%], τ^2 = 0.20, p < 0.001
Test for subgroup differences: χ^2_3 = 42.18, df = 3 (p < 0.001)
0.5 0.6 0.7 0.8 0.9

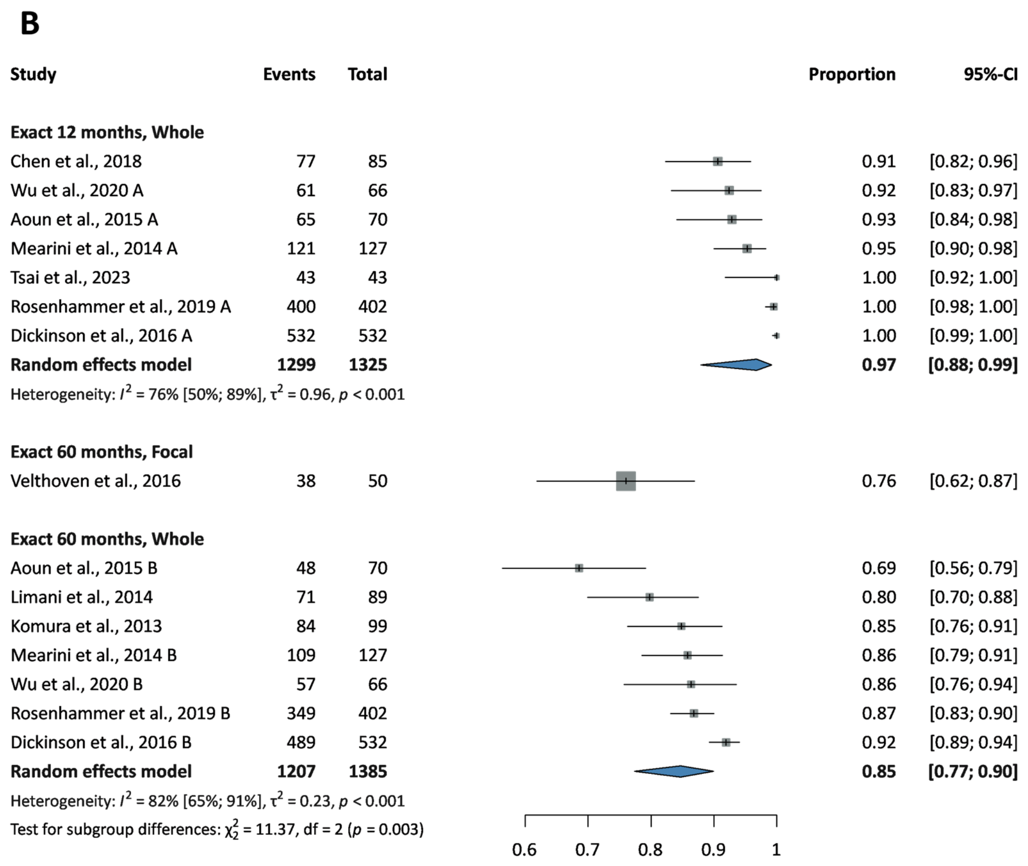
B
Study
Events
Total
Proportion
95%-CI
Exact 12 months, Whole
Chen et al., 2018 77 85 0.91 [0.82; 0.96]
Wu et al., 2020 A 61 66 0.92 [0.83; 0.97]
Aoun et al., 2015 A 65 70 0.93 [0.84; 0.98]
Mearini et al., 2014 A 121 127 0.95 [0.90; 0.98]
Tsai et al., 2023 43 43 1.00 [0.92; 1.00]
Rosenhammer et al., 2019 A 400 402 1.00 [0.98; 1.00]
Dickinson et al., 2016 A 532 532 1.00 [0.99; 1.00]
Random effects model 1299 1325 0.97 [0.88; 0.99]
Heterogeneity: I^2 = 76% [50%; 89%], τ^2 = 0.96, p < 0.001
Exact 60 months, Focal
Velthoven et al., 2016 38 50 0.76 [0.62; 0.87]
Exact 60 months, Whole
Aoun et al., 2015 B 48 70 0.69 [0.56; 0.79]
Limani et al., 2014 71 89 0.80 [0.70; 0.88]
Komura et al., 2013 84 99 0.85 [0.76; 0.91]
Mearini et al., 2014 B 109 127 0.86 [0.79; 0.91]
Wu et al., 2020 B 57 66 0.86 [0.76; 0.94]
Rosenhammer et al., 2019 B 349 402 0.87 [0.83; 0.90]
Dickinson et al., 2016 B 489 532 0.92 [0.89; 0.94]
Random effects model 1207 1385 0.85 [0.77; 0.90]
Heterogeneity: I^2 = 82% [65%; 91%], τ^2 = 0.23, p < 0.001
Test for subgroup differences: χ^2_2 = 11.37, df = 2 (p = 0.003)
0.6
0.7
0.8
0.9
1

C

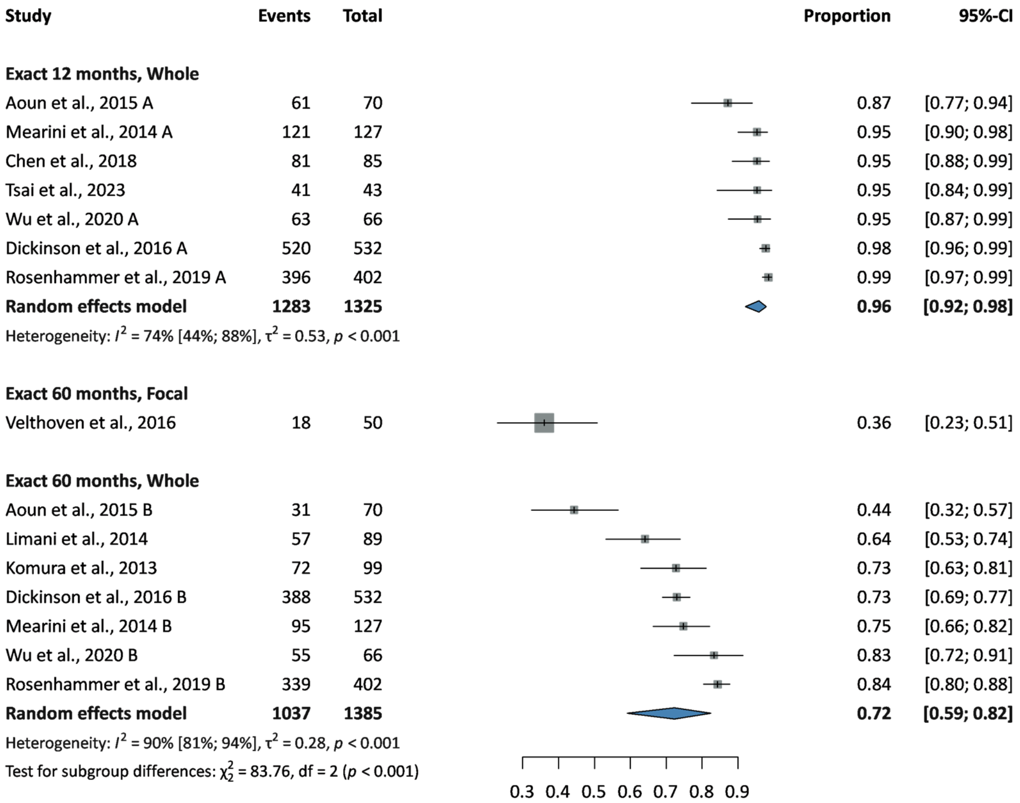

Figure S30. Summary forest plot of 12- and 60-month BFRS regarding focal and whole gland high-intensity focused ultrasound (Phoenix criteria). (A) BFRS among patients with low- and intermediate-risk prostate cancer; (B) BFRS among patients with low-risk prostate cancer; (C) BFRS among patients with intermediate-risk prostate cancer [28, 49, 51, 55, 64, 70, 73, 75, 76, 78-80, 83].

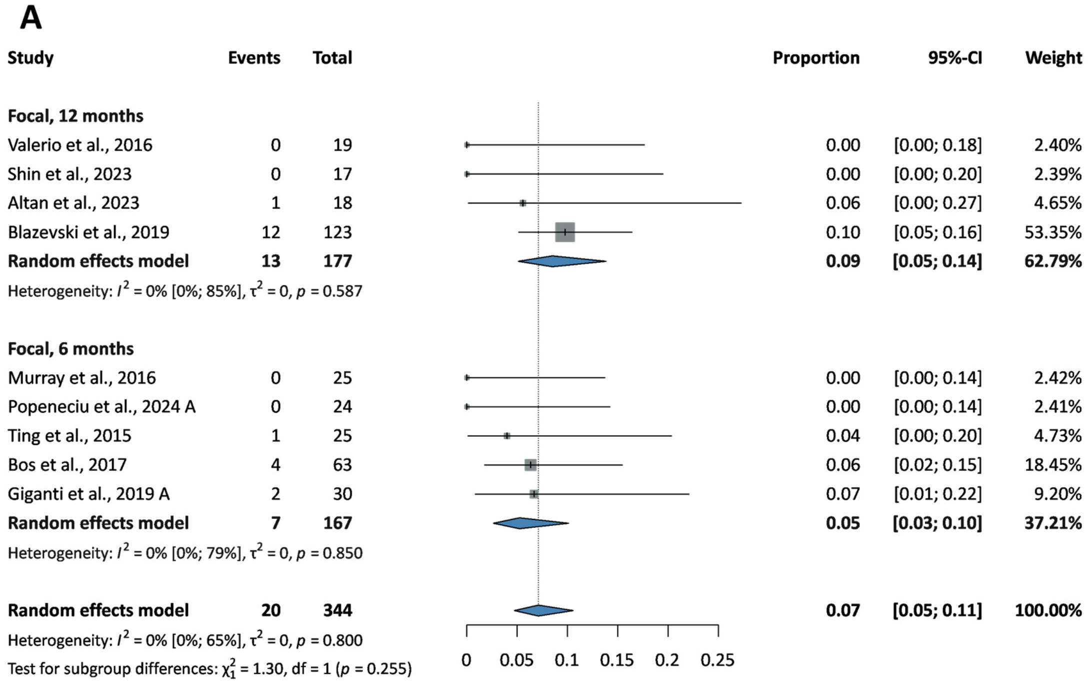
A
Study Events Total Proportion 95%-CI Weight
Focal, 12 months
Valerio et al., 2016 0 19 0.00 [0.00; 0.18] 2.40%
Shin et al., 2023 0 17 0.00 [0.00; 0.20] 2.39%
Altan et al., 2023 1 18 0.06 [0.00; 0.27] 4.65%
Blazevski et al., 2019 12 123 0.10 [0.05; 0.16] 53.35%
Random effects model 13 177 0.09 [0.05; 0.14] 62.79%
Heterogeneity: I^2 = 0% [0%; 85%], τ^2 = 0, p = 0.587
Focal, 6 months
Murray et al., 2016 0 25 0.00 [0.00; 0.14] 2.42%
Popeneciu et al., 2024 A 0 24 0.00 [0.00; 0.14] 2.41%
Ting et al., 2015 1 25 0.04 [0.00; 0.20] 4.73%
Bos et al., 2017 4 63 0.06 [0.02; 0.15] 18.45%
Giganti et al., 2019 A 2 30 0.07 [0.01; 0.22] 9.20%
Random effects model 7 167 0.05 [0.03; 0.10] 37.21%
Heterogeneity: I^2 = 0% [0%; 79%], τ^2 = 0, p = 0.850
Random effects model 20 344 0.07 [0.05; 0.11] 100.00%
Heterogeneity: I^2 = 0% [0%; 65%], τ^2 = 0, p = 0.800
Test for subgroup differences: χ^2_1 = 1.30, df = 1 (p = 0.255)
0 0.05 0.1 0.15 0.2 0.25

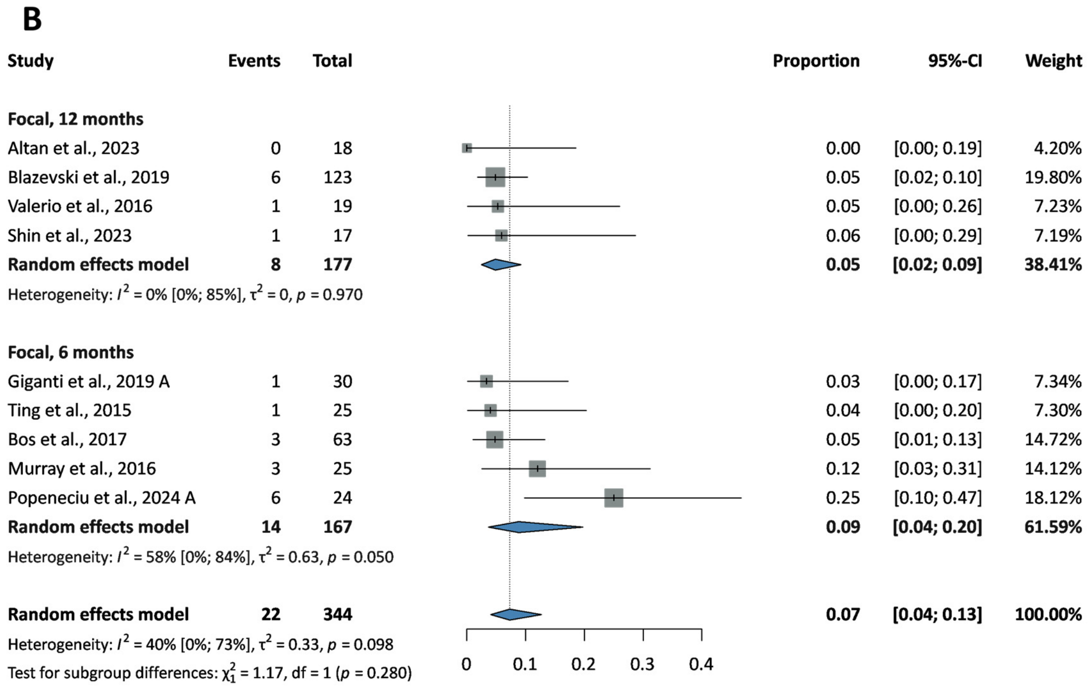
B
Study Events Total Proportion 95%-CI Weight
Focal, 12 months
Altan et al., 2023 0 18 0.00 [0.00; 0.19] 4.20%
Blazevski et al., 2019 6 123 0.05 [0.02; 0.10] 19.80%
Valerio et al., 2016 1 19 0.05 [0.00; 0.26] 7.23%
Shin et al., 2023 1 17 0.06 [0.00; 0.29] 7.19%
Random effects model 8 177 0.05 [0.02; 0.09] 38.41%
Heterogeneity: I^2 = 0% [0%; 85%], τ^2 = 0, p = 0.970
Focal, 6 months
Giganti et al., 2019 A 1 30 0.03 [0.00; 0.17] 7.34%
Ting et al., 2015 1 25 0.04 [0.00; 0.20] 7.30%
Bos et al., 2017 3 63 0.05 [0.01; 0.13] 14.72%
Murray et al., 2016 3 25 0.12 [0.03; 0.31] 14.12%
Popeneciu et al., 2024 A 6 24 0.25 [0.10; 0.47] 18.12%
Random effects model 14 167 0.09 [0.04; 0.20] 61.59%
Heterogeneity: I^2 = 58% [0%; 84%], τ^2 = 0.63, p = 0.050
Random effects model 22 344 0.07 [0.04; 0.13] 100.00%
Heterogeneity: I^2 = 40% [0%; 73%], τ^2 = 0.33, p = 0.098
Test for subgroup differences: χ^2_1 = 1.17, df = 1 (p = 0.280)
0 0.1 0.2 0.3 0.4

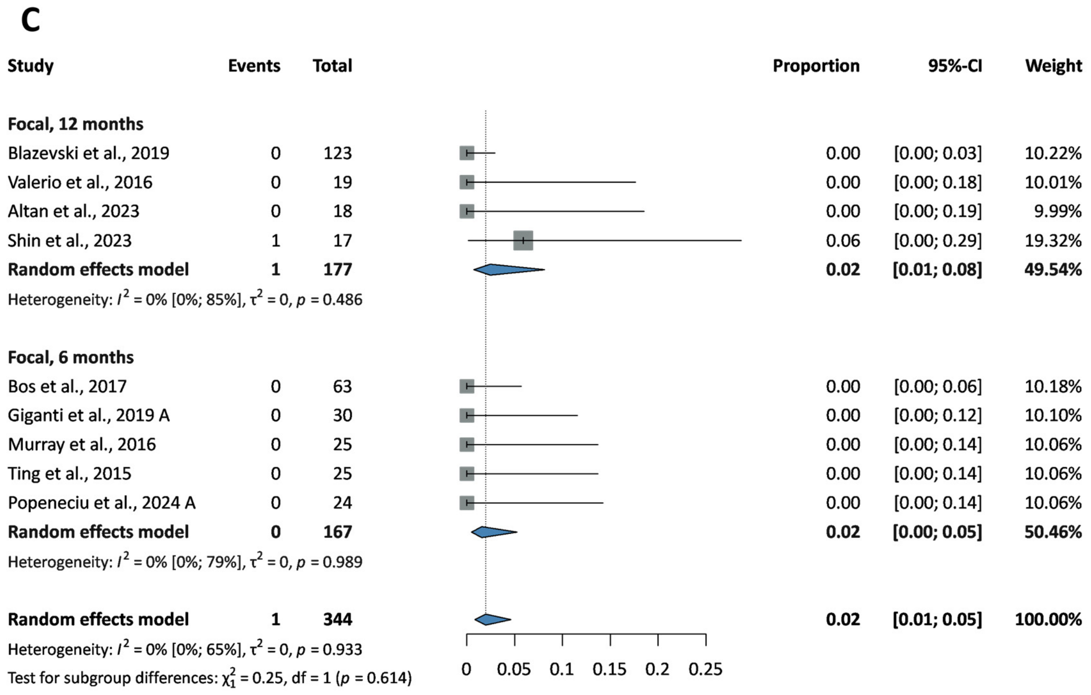

Figure S31. Summary forest plot of retreatments following focal irreversible electroporation: (A) second IRE; (B) radical treatment; and (C) hormonal therapy [4-7, 9-13].

A

Study	Follow up time	Events	Total	Proportion	95%-CI	Weight
**Focal**						
Bahn et al., 2012	Exact 12 months	2	73	0.03	[0.00; 0.10]	4.76%
Marra et al., 2021	Exact 5 months	4	121	0.03	[0.01; 0.08]	7.73%
Lepor et al., 2024	Exact 60 months	13	313	0.04	[0.02; 0.07]	13.64%
Gregg et al., 2021	.	1	23	0.04	[0.00; 0.22]	2.65%
Lian et al., 2015	Exact 12 months	2	41	0.05	[0.01; 0.17]	4.68%
Tokuda et al., 2023	.	1	16	0.06	[0.00; 0.30]	2.60%
Boissier et al., 2020 A	.	2	26	0.08	[0.01; 0.25]	4.57%
Durand et al., 2014	Exact 12 months	4	48	0.08	[0.02; 0.20]	7.47%
Ekish et al., 2013 A	Median 18 months	0	4	0.00	[0.00; 0.60]	1.34%
Lambert et al., 2007	.	3	25	0.12	[0.03; 0.31]	5.98%
Tourinho-Barbosa et al., 2020	Exact 12 months	16	111	0.14	[0.08; 0.22]	14.08%
**Random effects model**		**48**	**801**	**0.06**	**[0.04; 0.10]**	**69.51%**
Heterogeneity: I^2 = 46% [0%; 73%], τ^2 = 0.24, p = 0.045						
**Focal/Whole**						
Hale et al., 2013	Exact 12 months	1	26	0.04	[0.00; 0.20]	2.66%
Barqawi et al., 2017	Range 12-65 months	38	393	0.10	[0.07; 0.13]	17.48%
**Random effects model**		**39**	**419**	**0.09**	**[0.07; 0.13]**	**20.15%**
Heterogeneity: I^2 = 0%, τ^2 = 0, p = 0.341						
**Whole**						
Johansen et al., 2007	Median 21 months	1	64	0.02	[0.00; 0.08]	2.72%
Ekish et al., 2013 B	Median 18 months	0	17	0.00	[0.00; 0.20]	1.44%
Boissier et al., 2020 B	.	3	40	0.07	[0.02; 0.20]	6.19%
**Random effects model**		**4**	**121**	**0.05**	**[0.02; 0.11]**	**10.35%**
Heterogeneity: I^2 = 5% [0%; 90%], τ^2 = 0.04, p = 0.351						
**Random effects model**		**91**	**1341**	**0.07**	**[0.05; 0.09]**	**100.00%**
Heterogeneity: I^2 = 40% [0%; 67%], τ^2 = 0.15, p = 0.051						
Test for subgroup differences: χ^2_2 = 3.41, df = 2 (p = 0.182)						

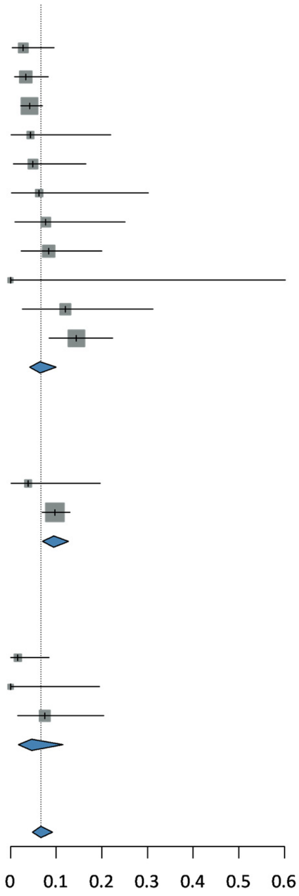

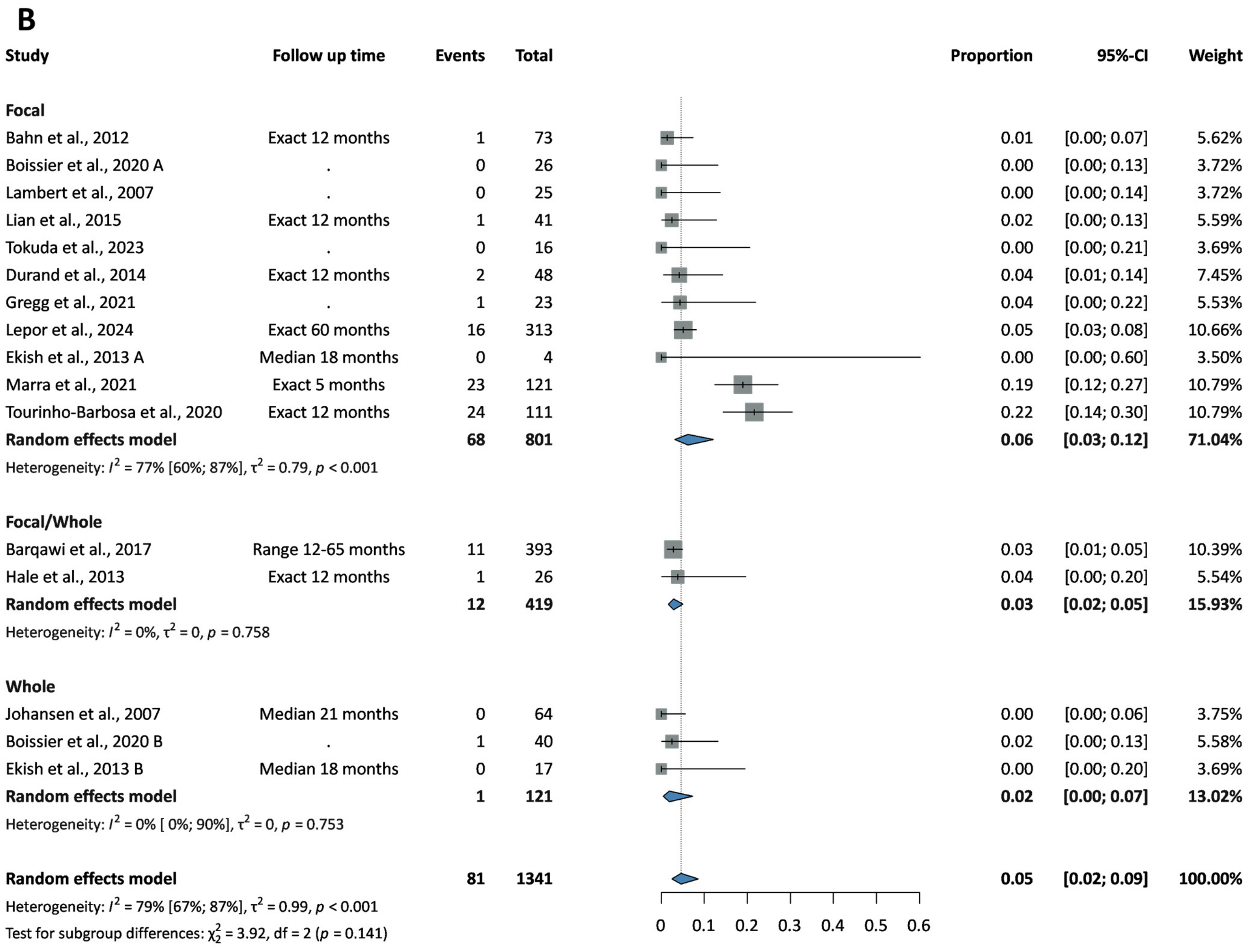
B
Study | Follow up time | Events | Total | Proportion | 95%-CI | Weight
Focal
Bahn et al., 2012 | Exact 12 months | 1 | 73 | 0.01 | [0.00; 0.07] | 5.62%
Boissier et al., 2020 A | . | 0 | 26 | 0.00 | [0.00; 0.13] | 3.72%
Lambert et al., 2007 | . | 0 | 25 | 0.00 | [0.00; 0.14] | 3.72%
Lian et al., 2015 | Exact 12 months | 1 | 41 | 0.02 | [0.00; 0.13] | 5.59%
Tokuda et al., 2023 | . | 0 | 16 | 0.00 | [0.00; 0.21] | 3.69%
Durand et al., 2014 | Exact 12 months | 2 | 48 | 0.04 | [0.01; 0.14] | 7.45%
Gregg et al., 2021 | . | 1 | 23 | 0.04 | [0.00; 0.22] | 5.53%
Lepor et al., 2024 | Exact 60 months | 16 | 313 | 0.05 | [0.03; 0.08] | 10.66%
Ekish et al., 2013 A | Median 18 months | 0 | 4 | 0.00 | [0.00; 0.60] | 3.50%
Marra et al., 2021 | Exact 5 months | 23 | 121 | 0.19 | [0.12; 0.27] | 10.79%
Tourinho-Barbosa et al., 2020 | Exact 12 months | 24 | 111 | 0.22 | [0.14; 0.30] | 10.79%
Random effects model | 68 | 801 | 0.06 | [0.03; 0.12] | 71.04%
Heterogeneity: I^2 = 77% [60%; 87%], τ^2 = 0.79, p < 0.001
Focal/Whole
Barqawi et al., 2017 | Range 12-65 months | 11 | 393 | 0.03 | [0.01; 0.05] | 10.39%
Hale et al., 2013 | Exact 12 months | 1 | 26 | 0.04 | [0.00; 0.20] | 5.54%
Random effects model | 12 | 419 | 0.03 | [0.02; 0.05] | 15.93%
Heterogeneity: I^2 = 0%, τ^2 = 0, p = 0.758
Whole
Johansen et al., 2007 | Median 21 months | 0 | 64 | 0.00 | [0.00; 0.06] | 3.75%
Boissier et al., 2020 B | . | 1 | 40 | 0.02 | [0.00; 0.13] | 5.58%
Ekish et al., 2013 B | Median 18 months | 0 | 17 | 0.00 | [0.00; 0.20] | 3.69%
Random effects model | 1 | 121 | 0.02 | [0.00; 0.07] | 13.02%
Heterogeneity: I^2 = 0% [0%; 90%], τ^2 = 0, p = 0.753
Random effects model | 81 | 1341 | 0.05 | [0.02; 0.09] | 100.00%
Heterogeneity: I^2 = 79% [67%; 87%], τ^2 = 0.99, p < 0.001
Test for subgroup differences: χ^2_2 = 3.92, df = 2 (p = 0.141)
0 0.1 0.2 0.3 0.4 0.5 0.6

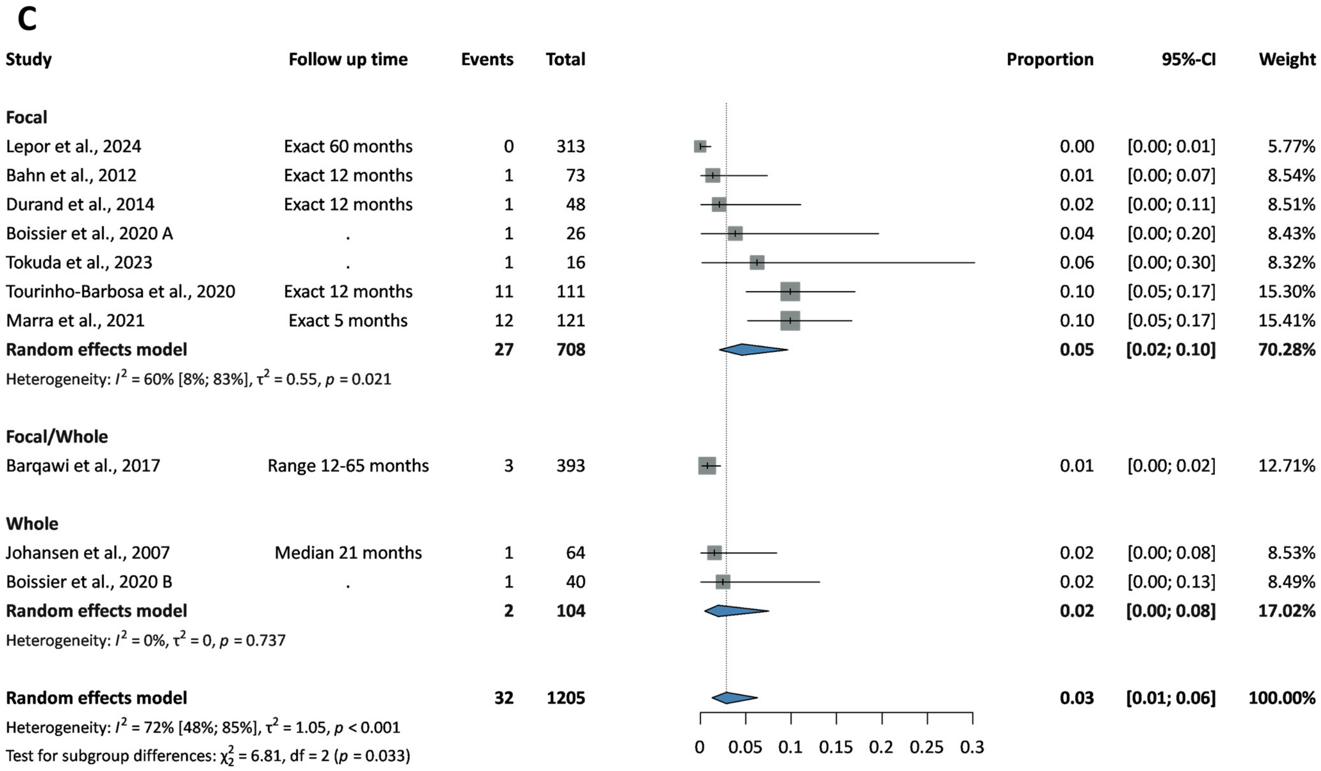

Figure S32. Summary forest plot of retreatments following focal and whole gland cryoablation: (A) second cryoablation; (B) radical treatment; and © hormonal therapy [15, 19, 20, 22-24, 26, 29-31, 36, 37, 44, 45].

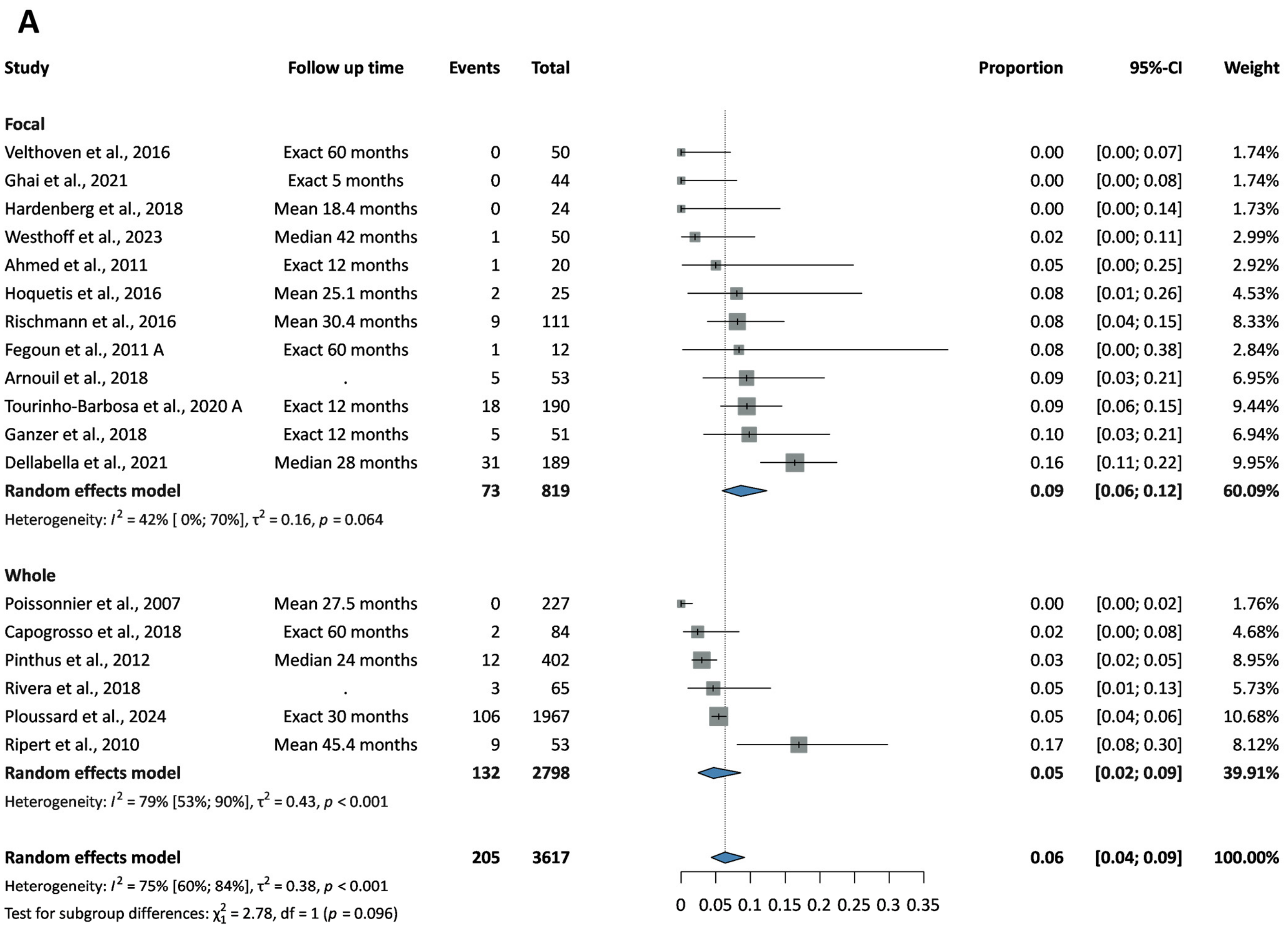
A
Study | Follow up time | Events | Total | Proportion | 95%-CI | Weight
Focal
Velthoven et al., 2016 | Exact 60 months | 0 | 50 | 0.00 | [0.00; 0.07] | 1.74%
Ghai et al., 2021 | Exact 5 months | 0 | 44 | 0.00 | [0.00; 0.08] | 1.74%
Hardenberg et al., 2018 | Mean 18.4 months | 0 | 24 | 0.00 | [0.00; 0.14] | 1.73%
Westhoff et al., 2023 | Median 42 months | 1 | 50 | 0.02 | [0.00; 0.11] | 2.99%
Ahmed et al., 2011 | Exact 12 months | 1 | 20 | 0.05 | [0.00; 0.25] | 2.92%
Hoquetis et al., 2016 | Mean 25.1 months | 2 | 25 | 0.08 | [0.01; 0.26] | 4.53%
Rischmann et al., 2016 | Mean 30.4 months | 9 | 111 | 0.08 | [0.04; 0.15] | 8.33%
Fegoun et al., 2011 A | Exact 60 months | 1 | 12 | 0.08 | [0.00; 0.38] | 2.84%
Arnouil et al., 2018 | . | 5 | 53 | 0.09 | [0.03; 0.21] | 6.95%
Tourinho-Barbosa et al., 2020 A | Exact 12 months | 18 | 190 | 0.09 | [0.06; 0.15] | 9.44%
Ganzer et al., 2018 | Exact 12 months | 5 | 51 | 0.10 | [0.03; 0.21] | 6.94%
Dellabella et al., 2021 | Median 28 months | 31 | 189 | 0.16 | [0.11; 0.22] | 9.95%
Random effects model | 73 | 819 | 0.09 | [0.06; 0.12] | 60.09%
Heterogeneity: I^2 = 42% [0%; 70%], τ^2 = 0.16, p = 0.064
Whole
Poissonnier et al., 2007 | Mean 27.5 months | 0 | 227 | 0.00 | [0.00; 0.02] | 1.76%
Capogrosso et al., 2018 | Exact 60 months | 2 | 84 | 0.02 | [0.00; 0.08] | 4.68%
Pinthus et al., 2012 | Median 24 months | 12 | 402 | 0.03 | [0.02; 0.05] | 8.95%
Rivera et al., 2018 | . | 3 | 65 | 0.05 | [0.01; 0.13] | 5.73%
Ploussard et al., 2024 | Exact 30 months | 106 | 1967 | 0.05 | [0.04; 0.06] | 10.68%
Ripert et al., 2010 | Mean 45.4 months | 9 | 53 | 0.17 | [0.08; 0.30] | 8.12%
Random effects model | 132 | 2798 | 0.05 | [0.02; 0.09] | 39.91%
Heterogeneity: I^2 = 79% [53%; 90%], τ^2 = 0.43, p < 0.001
Random effects model | 205 | 3617 | 0.06 | [0.04; 0.09] | 100.00%
Heterogeneity: I^2 = 75% [60%; 84%], τ^2 = 0.38, p < 0.001
Test for subgroup differences: χ^2_1 = 2.78, df = 1 (p = 0.096)
0 0.05 0.1 0.15 0.2 0.25 0.3 0.35

B

Study	Follow up time	Events	Total	Proportion	95%-CI	Weight
**Focal**						
Ghai et al., 2021	Exact 5 months	0	44	0.00	[0.00; 0.08]	1.81%
Hoquetis et al., 2016	Mean 25.1 months	0	25	0.00	[0.00; 0.14]	1.79%
Ahmed et al., 2011	Exact 12 months	0	20	0.00	[0.00; 0.17]	1.79%
Fegoun et al., 2011 A	Exact 60 months	0	12	0.00	[0.00; 0.26]	1.76%
Dellabella et al., 2021	Median 28 months	9	189	0.05	[0.02; 0.09]	7.74%
Arnouil et al., 2018	.	3	53	0.06	[0.01; 0.16]	5.50%
Velthoven et al., 2016	Exact 60 months	3	50	0.06	[0.01; 0.17]	5.49%
Rischmann et al., 2016	Mean 30.4 months	9	111	0.08	[0.04; 0.15]	7.69%
Ganzer et al., 2018	Exact 12 months	5	51	0.10	[0.03; 0.21]	6.55%
Tourinho-Barbosa et al., 2020 A	Exact 12 months	38	190	0.20	[0.15; 0.26]	9.05%
Hardenberg et al., 2018	Mean 18.4 months	5	24	0.21	[0.07; 0.42]	6.27%
Westhoff et al., 2023	Median 42 months	17	50	0.34	[0.21; 0.49]	8.13%
**Random effects model**		**89**	**819**	**0.09**	**[0.05; 0.16]**	**63.57%**
Heterogeneity: $I^2 = 78\%$ [63%; 87%], $\tau^2 = 0.69$, $p < 0.001$						
**Whole**						
Rivera et al., 2018	.	1	65	0.02	[0.00; 0.08]	3.03%
Pinthus et al., 2012	Median 24 months	10	402	0.02	[0.01; 0.05]	7.94%
Ploussard et al., 2024	Exact 30 months	163	1967	0.08	[0.07; 0.10]	9.56%
Poissonnier et al., 2007	Mean 27.5 months	22	227	0.10	[0.06; 0.14]	8.74%
Ripert et al., 2010	Mean 45.4 months	7	53	0.13	[0.05; 0.25]	7.15%
**Random effects model**		**203**	**2714**	**0.07**	**[0.04; 0.11]**	**36.43%**
Heterogeneity: $I^2 = 81\%$ [54%; 92%], $\tau^2 = 0.26$, $p < 0.001$						
**Random effects model**		**292**	**3533**	**0.08**	**[0.06; 0.12]**	**100.00%**
Heterogeneity: $I^2 = 83\%$ [74%; 89%], $\tau^2 = 0.46$, $p < 0.001$						
Test for subgroup differences: $\chi^2_1 = 0.80$, df = 1 ($p = 0.372$)						

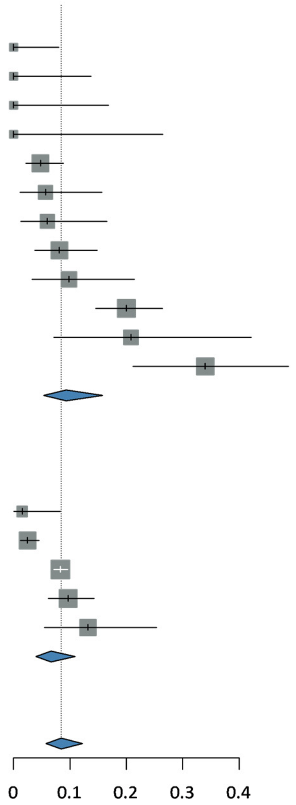

C

Study	Follow up time	Events	Total	Proportion	95%-CI	Weight
**Focal**						
Rischmann et al., 2016	Mean 30.4 months	0	111	0.00	[0.00; 0.03]	3.10%
Arnouil et al., 2018	.	0	53	0.00	[0.00; 0.07]	3.08%
Ganzer et al., 2018	Exact 12 months	0	51	0.00	[0.00; 0.07]	3.08%
Velthoven et al., 2016	Exact 60 months	0	50	0.00	[0.00; 0.07]	3.08%
Ghai et al., 2021	Exact 5 months	0	44	0.00	[0.00; 0.08]	3.08%
Hoquetis et al., 2016	Mean 25.1 months	0	25	0.00	[0.00; 0.14]	3.06%
Hardenberg et al., 2018	Mean 18.4 months	0	24	0.00	[0.00; 0.14]	3.06%
Westhoff et al., 2023	Median 42 months	1	50	0.02	[0.00; 0.11]	4.84%
Ahmed et al., 2011	Exact 12 months	0	20	0.00	[0.00; 0.17]	3.05%
Tourinho-Barbosa et al., 2020 A	Exact 12 months	7	190	0.04	[0.01; 0.07]	9.59%
Dellabella et al., 2021	Median 28 months	7	189	0.04	[0.02; 0.07]	9.59%
Fegoun et al., 2011 A	Exact 60 months	4	12	0.33	[0.10; 0.65]	7.64%
**Random effects model**		**19**	**819**	**0.03**	**[0.01; 0.06]**	**56.27%**
Heterogeneity: I^2 = 55% [13%; 76%], τ^2 = 0.81, p = 0.012						
**Whole**						
Pinthus et al., 2012	Median 24 months	4	402	0.01	[0.00; 0.03]	8.59%
Ripert et al., 2010	Mean 45.4 months	1	53	0.02	[0.00; 0.10]	4.84%
Ploussard et al., 2024	Exact 30 months	41	1967	0.02	[0.01; 0.03]	11.15%
Poissonnier et al., 2007	Mean 27.5 months	6	227	0.03	[0.01; 0.06]	9.35%
Rivera et al., 2018	.	9	65	0.14	[0.07; 0.25]	9.81%
**Random effects model**		**61**	**2714**	**0.03**	**[0.01; 0.07]**	**43.73%**
Heterogeneity: I^2 = 87% [72%; 94%], τ^2 = 0.92, p < 0.001						
**Random effects model**		**80**	**3533**	**0.03**	**[0.02; 0.05]**	**100.00%**
Heterogeneity: I^2 = 72% [55%; 83%], τ^2 = 0.74, p < 0.001						
Test for subgroup differences: χ^2_1 = 0.00, df = 1 (p = 0.976)						

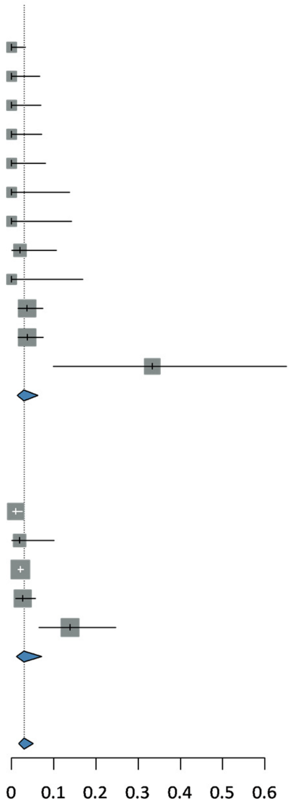

Figure S33. Summary forest plot of retreatments following focal and whole gland high-intensity focused ultrasound: A, second HIFU; B, radical treatment; and C, hormonal therapy [44, 46, 48, 51, 52, 54, 56, 57, 59-62, 64-66, 72, 82, 84].

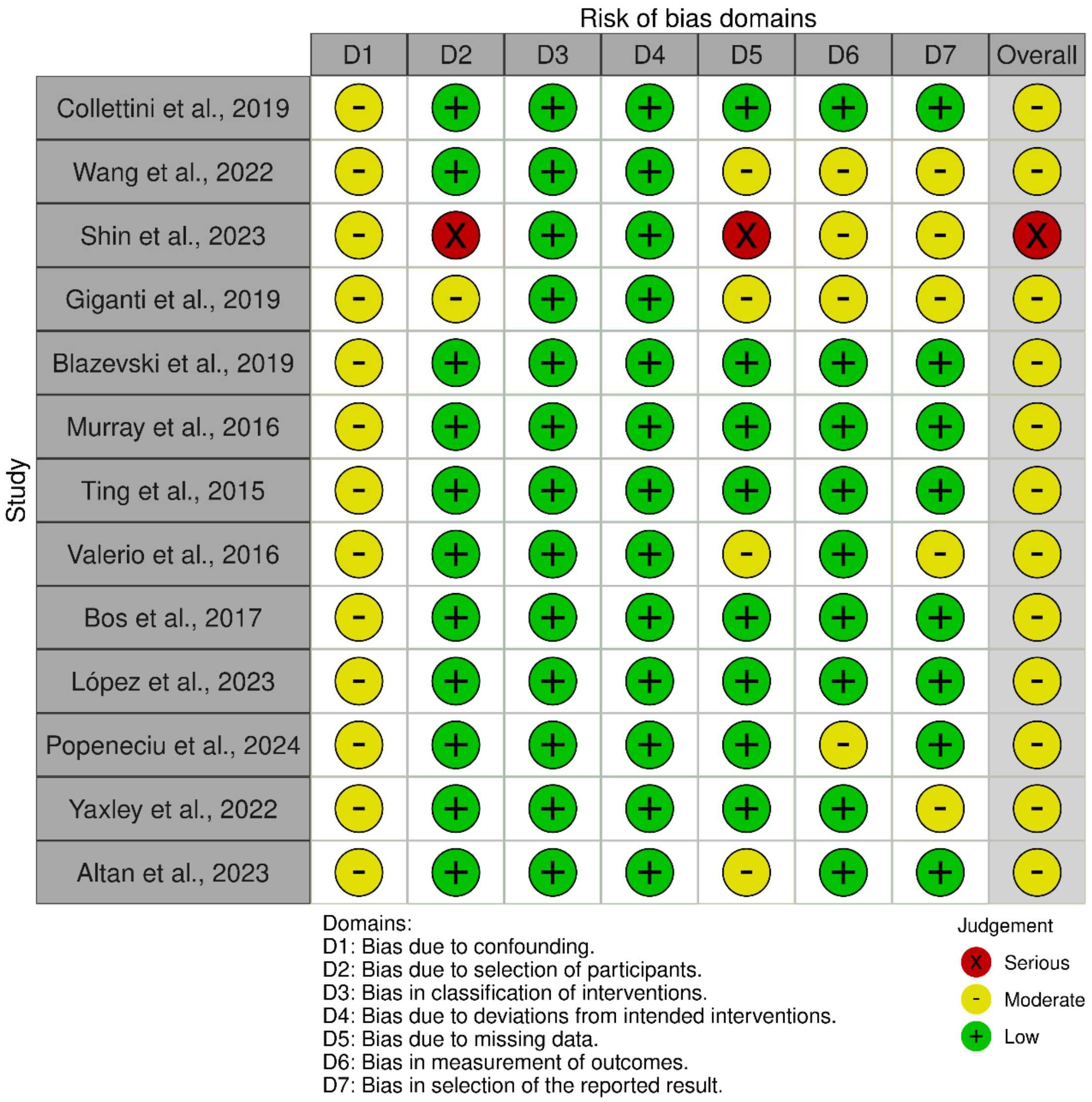

Figure S34. Risk of bias assessment using the ROBINS-I tool Regarding IRE [2-14].

Risk of bias domains

Study	D1	D2	D3	D4	D5	D6	D7	Overall
Tokuda et al., 2023	-	X	+	+	+	+	+	X
Wysock et al., 2020	-	-	+	+	X	-	-	X
Sze et al., 2019	-	X	+	+	+	+	+	X
Kim et al., 2012	-	X	+	+	X	X	-	X
Bahn et al., 2012	-	+	+	+	-	+	-	-
Barqawi et al., 2017	-	-	+	+	X	-	-	X
Hayek et al., 2008	X	X	+	+	X	-	-	X
Gregg et al., 2021	-	+	+	+	+	+	+	-
Lambert et al., 2007	-	-	+	+	+	+	+	-
Lian et al., 2015	-	-	+	+	+	+	+	-
Mendez et al., 2015	-	+	+	+	-	+	+	-
Marra et al., 2021	-	+	+	+	-	+	+	-
Barret et al., 2013	-	+	+	+	-	+	+	-
Enikeev et al., 2020	-	+	+	+	-	+	+	-
Durand et al., 2014	-	-	+	+	+	+	+	-
Hale et al., 2013	-	-	+	+	+	+	+	-
Boissier et al., 2020	-	+	+	+	+	+	+	-
Rodríguez et al., 2014	-	-	+	+	-	-	+	-
Elkjær et al., 2014	-	X	+	+	-	-	-	X
Guo et al., 2020	-	+	+	+	+	-	+	-
Aker et al., 2023	-	+	+	+	-	+	+	-
Ekish et al., 2013	-	X	+	+	-	-	-	X
Johansen et al., 2007	-	-	+	+	-	+	+	-
Cohen et al., 2008	-	+	+	+	-	+	+	-
Dhar et al, 2011	-	X	+	+	-	-	-	X
Grossgold et al., 2014	-	+	+	+	-	+	+	-
Liu et al., 2014	-	X	+	+	-	-	-	X
Mercader et al., 2020	-	-	+	+	-	+	+	-
Oishi et al., 2018	-	X	+	+	-	-	-	X
Tourinho-Barbosa et al., 2020	-	+	+	+	+	+	+	-
Lepor et al., 2024	-	-	+	+	-	+	+	-

Domains:
D1: Bias due to confounding.
D2: Bias due to selection of participants.
D3: Bias in classification of interventions.
D4: Bias due to deviations from intended interventions.
D5: Bias due to missing data.
D6: Bias in measurement of outcomes.
D7: Bias in selection of the reported result.

Judgement
X Serious
- Moderate
+ Low

Figure S35. Risk of bias assessment using the ROBINS-I tool Regarding cryoablation [15-45].

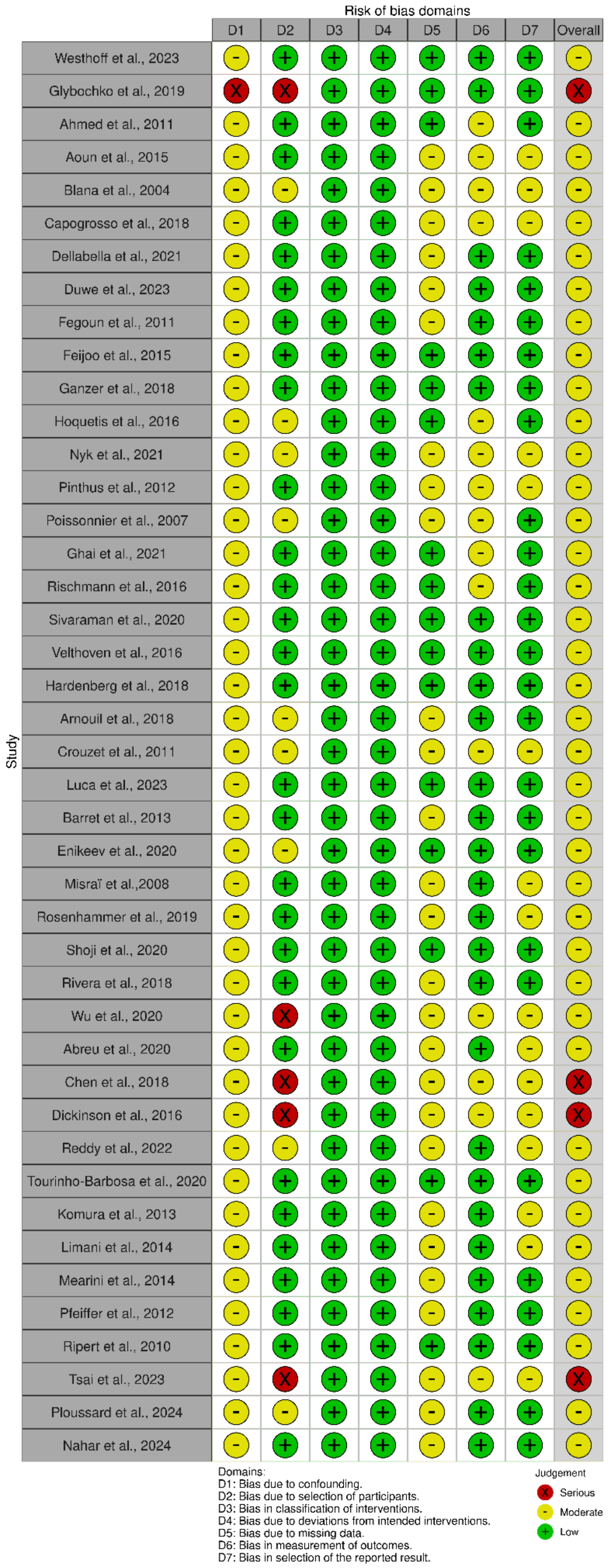
Risk of bias domains
D1 D2 D3 D4 D5 D6 D7 Overall
Study
Westhoff et al., 2023 - + + + + + + -
Glybochko et al., 2019 X X + + + + + X
Ahmed et al., 2011 - + + + + - + -
Aoun et al., 2015 - + + + - - - -
Blana et al., 2004 - - + + - - - -
Capogrosso et al., 2018 - + + + - - - -
Dellabella et al., 2021 - + + + - + + -
Duwe et al., 2023 - + + + - + + -
Fegoun et al., 2011 - + + + - + + -
Feijoo et al., 2015 - + + + + + + -
Ganzer et al., 2018 - + + + + + + -
Hoquetis et al., 2016 - - + + + - + -
Nyk et al., 2021 - - + + - - - -
Pinthus et al., 2012 - + + + - - - -
Poissonnier et al., 2007 - - + + - - + -
Ghai et al., 2021 - + + + + - + -
Rischmann et al., 2016 - + + + + - + -
Sivaraman et al., 2020 - + + + + + + -
Velthoven et al., 2016 - + + + + + + -
Hardenberg et al., 2018 - + + + + + + -
Arnouil et al., 2018 - - + + - + + -
Crouzet et al., 2011 - - + + - - - -
Luca et al., 2023 - + + + + + + -
Barret et al., 2013 - + + + - + + -
Enikeev et al., 2020 - - + + + + + -
Misraï et al.,2008 - + + + - + - -
Rosenhammer et al., 2019 - + + + - + - -
Shoji et al., 2020 - + + + + + + -
Rivera et al., 2018 - + + + - + + -
Wu et al., 2020 - X + + - - - -
Abreu et al., 2020 - + + + - + - -
Chen et al., 2018 - X + + - - - X
Dickinson et al., 2016 - X + + - - - X
Reddy et al., 2022 - - + + - + - -
Tourinho-Barbosa et al., 2020 - + + + + + + -
Komura et al., 2013 - + + + - + - -
Limani et al., 2014 - + + + - + - -
Mearini et al., 2014 - + + + - + + -
Pfeiffer et al., 2012 - + + + - + + -
Ripert et al., 2010 - + + + + + + -
Tsai et al., 2023 - X + + - - - X
Ploussard et al., 2024 - - + + - + + -
Nahar et al., 2024 - + + + - + + -
Domains:
D1: Bias due to confounding.
D2: Bias due to selection of participants.
D3: Bias in classification of interventions.
D4: Bias due to deviations from intended interventions.
D5: Bias due to missing data.
D6: Bias in measurement of outcomes.
D7: Bias in selection of the reported result.
Judgement
X Serious
- Moderate
+ Low

Figure S36. Risk of bias assessment using the ROBINS-I tool Regarding HIFU [27, 28, 44, 46-85].

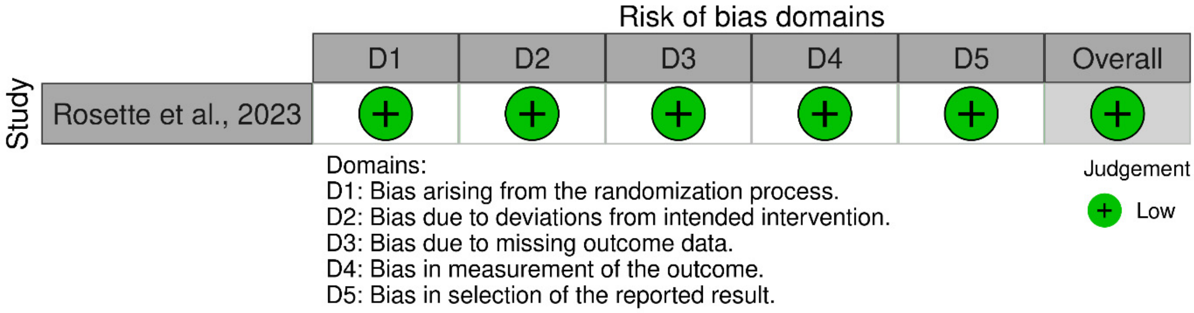

Figure S37. Risk of bias assessment using the RoB2 tool Regarding complication rates [1].

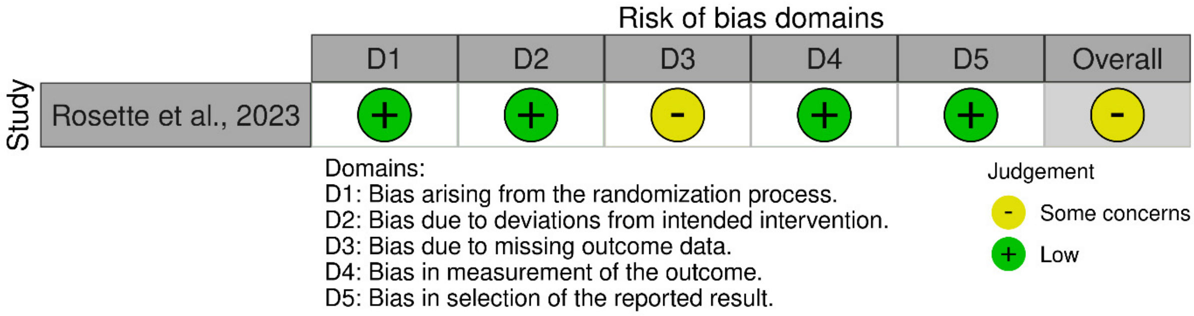

Figure S38. Risk of bias assessment using the RoB2 tool Regarding recurrence rates [1].

References

1. de la Rosette, J.; Dominguez-Escrig, J.; Zhang, K.; Teoh, J.; Barret, E.; Ramon-Borja, J. C.; Muir, G.; Bohr, J.; de Reijke, T.; Ng, C. F.; et al. A Multicenter, Randomized, Single-blind, 2-Arm Intervention Study Evaluating the Adverse Events and Quality of Life After Irreversible Electroporation for the Ablation of Localized Low-intermediate Risk Prostate Cancer. *J Urol* **2023**, *209* (2), 347-353. DOI: 10.1097/ju.0000000000003051

2. Collettini, F.; Enders, J.; Stephan, C.; Fischer, T.; Baur, A. D. J.; Penzkofer, T.; Busch, J.; Hamm, B.; Gebauer, B. Image-guided Irreversible Electroporation of Localized Prostate Cancer: Functional and Oncologic Outcomes. *Radiology* **2019**, *292* (1), 250-257. DOI: 10.1148/radiol.2019181987

3. Wang, H.; Xue, W.; Yan, W.; Yin, L.; Dong, B.; He, B.; Yu, Y.; Shi, W.; Zhou, Z.; Lin, H.; et al. Extended Focal Ablation of Localized Prostate Cancer With High-Frequency Irreversible Electroporation: A Nonrandomized Controlled Trial. *JAMA Surg* **2022**, *157* (8), 693-700. DOI: 10.1001/jamasurg.2022.2230

4. Shin, D.; Yoon, C. E.; Kwon, H. J.; Moon, H. W.; Park, Y. H.; Cho, H. J.; Ha, U. S.; Hong, S. H.; Park, S. Y.; Ha, S.; et al. Irreversible electroporation for prostate cancer using PSMA PET-CT. *Prostate Int* **2023**, *11* (1), 40-45. DOI: 10.1016/j.prnil.2022.08.004

5. Giganti, F.; Stabile, A.; Giona, S.; Marenco, J.; Orczyk, C.; Moore, C. M.; Allen, C.; Kirkham, A.; Emberton, M.; Punwani, S. Prostate cancer treated with irreversible electroporation: MRI-based volumetric analysis and oncological outcome. *Magn Reson Imaging* **2019**, *58*, 143-147. DOI: 10.1016/j.mri.2019.02.003
6. Blazevski, A.; Scheltema, M. J.; Yuen, B.; Masand, N.; Nguyen, T. V.; Delprado, W.; Shnier, R.; Haynes, A. M.; Cusick, T.; Thompson, J.; et al. Oncological and Quality-of-life Outcomes Following Focal Irreversible Electroporation as Primary Treatment for Localised Prostate Cancer: A Biopsy-monitored Prospective Cohort. *Eur Urol Oncol* **2020**, *3* (3), 283-290. DOI: 10.1016/j.euo.2019.04.008
7. Popeneciu, I. V.; Mohr, M. N.; Strauß, A.; Leitsmann, C.; Trojan, L.; Reichert, M. Personalized Treatment Strategy in "Low-Risk Prostate Cancer Active Surveillance Candidates" Using Irreversible Electroporation: Prospective Evaluation of Feasibility, Morbidity, Functional and Oncological Outcomes. *World J Mens Health* **2024**, *42* (4), 821-829. DOI: 10.5534/wjmh.230097
8. Yaxley, W. J.; Gianduzzo, T.; Kua, B.; Oxford, R.; Yaxley, J. W. Focal therapy for prostate cancer with irreversible electroporation: Oncological and functional results of a single institution study. *Investig Clin Urol* **2022**, *63* (3), 285-293. DOI: 10.4111/icu.20210472
9. Altan Ş, A.; Güleryüz Kızıl, P.; Tarhan, N.; Adsan, O. One-year Follow-up Results of Transperineal Biopsy For Patients Undergoing Irreversible Electroporation Treatment in Localized Prostate Cancer. *Urol Res Pract* **2023**, *49* (6), 381-386. DOI: 10.5152/tud.2023.23150
10. Murray, K. S.; Ehdaie, B.; Musser, J.; Mashni, J.; Srimathveeravalli, G.; Durack, J. C.; Solomon, S. B.; Coleman, J. A. Pilot Study to Assess Safety and Clinical Outcomes of Irreversible Electroporation for Partial Gland Ablation in Men with Prostate Cancer. *J Urol* **2016**, *196* (3), 883-890. DOI: 10.1016/j.juro.2016.02.2986
11. Ting, F.; Tran, M.; Böhm, M.; Siriwardana, A.; Van Leeuwen, P. J.; Haynes, A. M.; Delprado, W.; Shnier, R.; Stricker, P. D. Focal irreversible electroporation for prostate cancer: functional outcomes and short-term oncological control. *Prostate Cancer Prostatic Dis* **2016**, *19* (1), 46-52. DOI: 10.1038/pcan.2015.47
12. Valerio, M.; Dickinson, L.; Ali, A.; Ramachadran, N.; Donaldson, I.; McCartan, N.; Freeman, A.; Ahmed, H. U.; Emberton, M. Nanoknife Electroporation Ablation Trial: A Prospective Development Study Investigating Focal Irreversible Electroporation for Localized Prostate Cancer. *J Urol* **2017**, *197* (3 Pt 1), 647-654. DOI: 10.1016/j.juro.2016.09.091
13. van den Bos, W.; Scheltema, M. J.; Siriwardana, A. R.; Kalsbeek, A. M. F.; Thompson, J. E.; Ting, F.; Böhm, M.; Haynes, A. M.; Shnier, R.; Delprado, W.; et al. Focal irreversible electroporation as primary treatment for localized prostate cancer. *BJU Int* **2018**, *121* (5), 716-724. DOI: 10.1111/bju.13983
14. Miñana López, B.; Andrés Boville, G.; Barbas Bernardos, G.; Ancizu Marckert, X.; Torres Roca, M.; Labairu Huerta, L.; Villacampa Aubá, F.; Ramón de Fata Chillón, F.; Sanz Ortega, J.; Abengózar Muela, M.; et al. Focal Therapy of Prostate Cancer Index Lesion With Irreversible Electroporation. A Prospective Study With a Median Follow-up of 3 Years. *J Urol* **2023**, *209* (1), 261-270. DOI: 10.1097/ju.0000000000002970
15. Tokuda, B.; Yamada, K.; Takahata, A.; Fujihara, A.; Iwata, T.; Ukimura, O.; Yamada, K. Time-course changes in multiparametric magnetic resonance imaging following focal cryotherapy for localized prostate cancer: Initial experience. *Eur J Radiol* **2023**, *160*, 110714. DOI: 10.1016/j.ejrad.2023.110714
16. Wysock, J. S.; Becher, E.; Gogaj, R.; Velazquez, N.; Lepor, H. Early oncological control following partial gland cryo-ablation: a prospective experience specifying reflex MRI guided biopsy of the ablation zone. *Prostate Cancer and Prostatic Diseases* **2021**, *24* (1), 114-119. DOI: 10.1038/s41391-020-0244-0
17. Sze, C.; Tsivian, E.; Tay, K. J.; Schulman, A. A.; Davis, L. G.; Gupta, R. T.; Polascik, T. J. Anterior gland focal cryoablation: proof-of-concept primary prostate cancer treatment in select men with localized anterior

cancers detected by multi-parametric magnetic resonance imaging. *BMC Urol* **2019**, *19* (1), 127. DOI: 10.1186/s12894-019-0562-5
18. Kim, F. J.; Cerqueira, M. A.; Almeida, J. C.; Pompeo, A.; Sehrt, D.; Calheiros, J. M.; Martins, F. A.; Molina, W. R. Initial brazilian experience in the treatment of localized prostate cancer using a new generation cryotechnology: feasibility study. *Int Braz J Urol* **2012**, *38* (5), 620-626. DOI: 10.1590/s1677-55382012000500006
19. Bahn, D.; de Castro Abreu, A. L.; Gill, I. S.; Hung, A. J.; Silverman, P.; Gross, M. E.; Lieskovsky, G.; Ukimura, O. Focal cryotherapy for clinically unilateral, low-intermediate risk prostate cancer in 73 men with a median follow-up of 3.7 years. *Eur Urol* **2012**, *62* (1), 55-63. DOI: 10.1016/j.eururo.2012.03.006
20. Barqawi, A. B.; Huebner, E.; Krughoff, K.; O'Donnell, C. I. Prospective Outcome Analysis of the Safety and Efficacy of Partial and Complete Cryoablation in Organ-confined Prostate Cancer. *Urology* **2018**, *112*, 126-131. DOI: 10.1016/j.urology.2017.10.029
21. El Hayek, O. R.; Alfer, W., Jr.; Reggio, E.; Pompeo, A. C.; Arap, S.; Lucon, A. M.; Srougi, M. Prostate cryoablation: prospective analysis comparing high- and low-risk prostate cancer outcomes. *Urol Int* **2008**, *81* (2), 186-190. DOI: 10.1159/000144058
22. Gregg, J. R.; Borregales, L. D.; Choi, H.; Lozano, M.; McRae, S. E.; Venkatesan, A. M.; Davis, J. W.; Nogueras-Gonzalez, G. M.; Pisters, L. L.; Ward, J. F. Prospective trial of regional (hockey-stick) prostate cryoablation: oncologic and quality of life outcomes. *World J Urol* **2021**, *39* (9), 3259-3264. DOI: 10.1007/s00345-020-03575-4
23. Lambert, E. H.; Bolte, K.; Masson, P.; Katz, A. E. Focal cryosurgery: encouraging health outcomes for unifocal prostate cancer. *Urology* **2007**, *69* (6), 1117-1120. DOI: 10.1016/j.urology.2007.02.047
24. Lian, H.; Zhuang, J.; Yang, R.; Qu, F.; Wang, W.; Lin, T.; Guo, H. Focal cryoablation for unilateral low-intermediate-risk prostate cancer: 63-month mean follow-up results of 41 patients. *Int Urol Nephrol* **2016**, *48* (1), 85-90. DOI: 10.1007/s11255-015-1140-8
25. Mendez, M. H.; Passoni, N. M.; Pow-Sang, J.; Jones, J. S.; Polascik, T. J. Comparison of Outcomes Between Preoperatively Potent Men Treated with Focal Versus Whole Gland Cryotherapy in a Matched Population. *J Endourol* **2015**, *29* (10), 1193-1198. DOI: 10.1089/end.2014.0881
26. Marra, G.; Soeterik, T.; Oreggia, D.; Tourinho-Barbosa, R.; Moschini, M.; Filippini, C.; van Melick, H. H. E.; van den Bergh, R. C. N.; Gontero, P.; Cathala, N.; et al. Long-term Outcomes of Focal Cryotherapy for Low- to Intermediate-risk Prostate Cancer: Results and Matched Pair Analysis with Active Surveillance. *Eur Urol Focus* **2022**, *8* (3), 701-709. DOI: 10.1016/j.euf.2021.04.008
27. Barret, E.; Ahallal, Y.; Sanchez-Salas, R.; Galiano, M.; Cosset, J. M.; Validire, P.; Macek, P.; Durand, M.; Prapotnich, D.; Rozet, F.; et al. Morbidity of focal therapy in the treatment of localized prostate cancer. *Eur Urol* **2013**, *63* (4), 618-622. DOI: 10.1016/j.eururo.2012.11.057
28. Enikeev, D.; Taratkin, M.; Amosov, A.; Rivas, J. G.; Podoinitsin, A.; Potoldykova, N.; Karageziyan, M.; Glybochko, P.; Barret, E. Whole-gland ablation therapy versus active surveillance for low-risk prostate cancer: a prospective study. *Cent European J Urol* **2020**, *73* (2), 127-133. DOI: 10.5173/ceju.2020.0009
29. Durand, M.; Barret, E.; Galiano, M.; Rozet, F.; Sanchez-Salas, R.; Ahallal, Y.; Macek, P.; Gaya, J. M.; Cerruti, J.; Devilliers, H.; et al. Focal cryoablation: a treatment option for unilateral low-risk prostate cancer. *BJU Int* **2014**, *113* (1), 56-64. DOI: 10.1111/bju.12370
30. Hale, Z.; Miyake, M.; Palacios, D. A.; Rosser, C. J. Focal cryosurgical ablation of the prostate: a single institute's perspective. *BMC Urol* **2013**, *13*, 2. DOI: 10.1186/1471-2490-13-2

31. Bossier, R.; Sanguedolce, F.; Territo, A.; Vanacore, D.; Martínez, C.; Regis, F.; Gallioli, A.; Mercade, A.; Mosquera, L.; Aumatell, J.; et al. Whole and hemi-gland cryoablation for primary localized prostate cancer: Short and medium-term oncological and functional outcomes. *Actas Urol Esp (Engl Ed)* **2020**, *44* (3), 172-178. DOI: 10.1016/j.acuro.2019.10.003
32. Rodríguez, S. A.; Arias Fúnez, F.; Bueno Bravo, C.; Rodríguez-Patrón Rodríguez, R.; Sanz Mayayo, E.; Palacios, V. H.; Burgos Revilla, F. J. Cryotherapy for primary treatment of prostate cancer: intermediate term results of a prospective study from a single institution. *Prostate Cancer* **2014**, *2014*, 571576. DOI: 10.1155/2014/571576
33. Elkjær, M. C.; Borre, M. Oncological outcome after primary prostate cryoablation compared with radical prostatectomy: a single-centre experience. *Scand J Urol* **2014**, *48* (1), 27-33. DOI: 10.3109/21681805.2013.792102
34. Guo, X. X.; Liu, S. J.; Wang, M.; Hou, H. M.; Wang, X.; Zhang, Z. P.; Liu, M.; Wang, J. Y. Comparing the Oncological Outcomes of Cryoablation vs. Radical Prostatectomy in Low-Intermediate Risk Localized Prostate Cancer. *Front Oncol* **2020**, *10*, 1489. DOI: 10.3389/fonc.2020.01489
35. Aker, M. N.; Brisbane, W. G.; Kwan, L.; Gonzalez, S.; Priester, A. M.; Kinnaird, A.; Delfin, M. K.; Felker, E.; Sisk, A. E.; Kuppermann, D.; et al. Cryotherapy for partial gland ablation of prostate cancer: Oncologic and safety outcomes. *Cancer Med* **2023**, *12* (8), 9351-9362. DOI: 10.1002/cam4.5692
36. Al Ekish, S.; Nayeemuddin, M.; Maddox, M.; Pareek, G. The role of cryosurgery of the prostate for nonsurgical candidates. *Jsls* **2013**, *17* (3), 423-428. DOI: 10.4293/108680813x13693422518551
37. Bjerklund Johansen, T. E. [Cryosurgical ablation as primary treatment in prostate cancer patients]. *Actas Urol Esp* **2007**, *31* (6), 651-659. DOI: 10.1016/s0210-4806(07)73702-6
38. Cohen, J. K.; Miller, R. J., Jr.; Ahmed, S.; Lotz, M. J.; Baust, J. Ten-year biochemical disease control for patients with prostate cancer treated with cryosurgery as primary therapy. *Urology* **2008**, *71* (3), 515-518. DOI: 10.1016/j.urology.2007.09.059
39. Dhar, N.; Ward, J. F.; Cher, M. L.; Jones, J. S. Primary full-gland prostate cryoablation in older men (> age of 75 years): results from 860 patients tracked with the COLD Registry. *BJU Int* **2011**, *108* (4), 508-512. DOI: 10.1111/j.1464-410X.2011.10238.x
40. Grossgold, E.; Given, R.; Ruckle, H.; Jones, J. S. Does neoadjuvant androgen deprivation therapy before primary whole gland cryoablation of the prostate affect the outcome? *Urology* **2014**, *83* (2), 379-383. DOI: 10.1016/j.urology.2013.08.061
41. Liu, Y. Y.; Chiang, P. H.; Chuang, Y. C.; Lee, W. C.; Cheng, Y. T.; Wang, H. J. Predictors of prostate-specific antigen biochemical recurrence in patients undergoing primary whole-gland prostate cryoablation. *Ann Surg Oncol* **2015**, *22* (5), 1612-1617. DOI: 10.1245/s10434-014-3942-9
42. Mercader, C.; Musquera, M.; Franco, A.; Alcaraz, A.; Ribal, M. J. Primary cryotherapy for localized prostate cancer treatment. *Aging Male* **2020**, *23* (5), 1460-1466. DOI: 10.1080/13685538.2020.1796960
43. Oishi, M.; Gill, I. S.; Ashrafi, A. N.; Lin-Brande, M.; Nassiri, N.; Shin, T.; Bove, A.; Cacciamani, G. E.; Ukimura, O.; Bahn, D. K.; et al. Primary Whole-gland Cryoablation for Prostate Cancer: Biochemical Failure and Clinical Recurrence at 5.6 Years of Follow-up. *Eur Urol* **2019**, *75* (2), 208-214. DOI: 10.1016/j.eururo.2018.09.004
44. Tourinho-Barbosa, R. R.; Sanchez-Salas, R.; Claros, O. R.; Collura-Merlier, S.; Bakavicius, A.; Carneiro, A.; Stabile, A.; Moschini, M.; Cathala, N.; Tobias-Machado, M.; et al. Focal Therapy for Localized Prostate Cancer with Either High Intensity Focused Ultrasound or Cryoablation: A Single Institution Experience. *J Urol* **2020**, *203* (2), 320-330. DOI: 10.1097/ju.0000000000000506

45. Lepor, H.; Rapoport, E.; Tafa, M.; Gogaj, R.; Wysock, J. S. Five-year Oncologic Outcomes Following Primary Partial Gland Cryo-ablation Prospective Cohort Study of Men With Intermediate-risk Prostate Cancer. *Urology* **2025**, *196*, 189-195. DOI: 10.1016/j.urology.2024.10.039
46. Westhoff, N.; Ernst, R.; Kowalewski, K. F.; Derigs, F.; Neuberger, M.; Nörenberg, D.; Popovic, Z. V.; Ritter, M.; Stephan Michel, M.; von Hardenberg, J. Medium-term Oncological Efficacy and Patient-reported Outcomes After Focal High-intensity Focused Ultrasound: The FOXPRO Trial. *Eur Urol Focus* **2023**, *9* (2), 283-290. DOI: 10.1016/j.euf.2022.10.006
47. Glybochko, P. V.; Amosov, A. V.; Krupinov, G. E.; Petrovskii, N. V.; Lumpov, I. S. Hemiablation of Localized Prostate Cancer by High-Intensity Focused Ultrasound: A Series of 35 Cases. *Oncology* **2019**, *97* (1), 44-48. DOI: 10.1159/000499739
48. Ahmed, H. U.; Freeman, A.; Kirkham, A.; Sahu, M.; Scott, R.; Allen, C.; Van der Meulen, J.; Emberton, M. Focal therapy for localized prostate cancer: a phase I/II trial. *J Urol* **2011**, *185* (4), 1246-1254. DOI: 10.1016/j.juro.2010.11.079
49. Aoun, F.; Limani, K.; Peltier, A.; Marcelis, Q.; Zanaty, M.; Chamoun, A.; Vanden Bossche, M.; Roumeguère, T.; van Velthoven, R. High Intensity Focused Ultrasound versus Brachytherapy for the Treatment of Localized Prostate Cancer: A Matched-Pair Analysis. *Adv Urol* **2015**, *2015*, 350324. DOI: 10.1155/2015/350324
50. Blana, A.; Walter, B.; Rogenhofer, S.; Wieland, W. F. High-intensity focused ultrasound for the treatment of localized prostate cancer: 5-year experience. *Urology* **2004**, *63* (2), 297-300. DOI: 10.1016/j.urology.2003.09.020
51. Capogrosso, P.; Barret, E.; Sanchez-Salas, R.; Nunes-Silva, I.; Rozet, F.; Galiano, M.; Ventimiglia, E.; Briganti, A.; Salonia, A.; Montorsi, F.; et al. Oncological and functional outcomes of elderly men treated with HIFU vs. minimally invasive radical prostatectomy: A propensity score analysis. *Eur J Surg Oncol* **2018**, *44* (1), 185-191. DOI: 10.1016/j.ejso.2017.11.008
52. Dellabella, M.; Branchi, A.; Di Rosa, M.; Pucci, M.; Gasparri, L.; Claudini, R.; Carnevali, F.; Cecchini, S.; Castellani, D. Oncological and functional outcome after partial prostate HIFU ablation with Focal-One(®): a prospective single-center study. *Prostate Cancer Prostatic Dis* **2021**, *24* (4), 1189-1197. DOI: 10.1038/s41391-021-00390-9
53. Duwe, G.; Boehm, K.; Haack, M.; Sparwasser, P.; Brandt, M. P.; Mager, R.; Tsaur, I.; Haferkamp, A.; Höfner, T. Single-center, prospective phase 2 trial of high-intensity focused ultrasound (HIFU) in patients with unilateral localized prostate cancer: good functional results but oncologically not as safe as expected. *World J Urol* **2023**, *41* (5), 1293-1299. DOI: 10.1007/s00345-023-04352-9
54. El Fegoun, A. B.; Barret, E.; Prapotnich, D.; Soon, S.; Cathelineau, X.; Rozet, F.; Galiano, M.; Sanchez-Salas, R.; Vallancien, G. Focal therapy with high-intensity focused ultrasound for prostate cancer in the elderly. A feasibility study with 10 years follow-up. *Int Braz J Urol* **2011**, *37* (2), 213-219; discussion 220-212. DOI: 10.1590/s1677-55382011000200008
55. Feijoo, E. R.; Sivaraman, A.; Barret, E.; Sanchez-Salas, R.; Galiano, M.; Rozet, F.; Prapotnich, D.; Cathala, N.; Mombet, A.; Cathelineau, X. Focal High-intensity Focused Ultrasound Targeted Hemiablation for Unilateral Prostate Cancer: A Prospective Evaluation of Oncologic and Functional Outcomes. *Eur Urol* **2016**, *69* (2), 214-220. DOI: 10.1016/j.eururo.2015.06.018
56. Ganzer, R.; Hadaschik, B.; Pahernik, S.; Koch, D.; Baumunk, D.; Kuru, T.; Heidenreich, A.; Stolzenburg, J. U.; Schostak, M.; Blana, A. Prospective Multicenter Phase II Study on Focal Therapy (Hemiablation) of the Prostate with High Intensity Focused Ultrasound. *J Urol* **2018**, *199* (4), 983-989. DOI: 10.1016/j.juro.2017.10.033

57. Hoquetis, L.; Malavaud, B.; Game, X.; Beauval, J. B.; Portalez, D.; Soulie, M.; Rischmann, P. MRI evaluation following partial HIFU therapy for localized prostate cancer: A single-center study. *Prog Urol* **2016**, *26* (9), 517-523. DOI: 10.1016/j.purol.2016.07.006
58. Nyk, Ł.; Michalak, W.; Szempliński, S.; Woźniak, R.; Zagożdżon, B.; Krajewski, W.; Kryst, P.; Kamecki, H.; Poletajew, S. High-Intensity Focused-Ultrasound Focal Therapy Versus Laparoscopic Radical Prostatectomy: A Comparison of Oncological and Functional Outcomes in Low- and Intermediate-Risk Prostate Cancer Patients. *J Pers Med* **2022**, *12* (2). DOI: 10.3390/jpm12020251
59. Pinthus, J. H.; Farrokhyar, F.; Hassouna, M. M.; Woods, E.; Whelan, K.; Shayegan, B.; Orovan, W. L. Single-session primary high-intensity focused ultrasonography treatment for localized prostate cancer: biochemical outcomes using third generation-based technology. *BJU Int* **2012**, *110* (8), 1142-1148. DOI: 10.1111/j.1464-410X.2012.10945.x
60. Poissonnier, L.; Chapelon, J. Y.; Rouvière, O.; Curiel, L.; Bouvier, R.; Martin, X.; Dubernard, J. M.; Gelet, A. Control of prostate cancer by transrectal HIFU in 227 patients. *Eur Urol* **2007**, *51* (2), 381-387. DOI: 10.1016/j.eururo.2006.04.012
61. Ghai, S.; Finelli, A.; Corr, K.; Chan, R.; Jokhu, S.; Li, X.; McCluskey, S.; Konukhova, A.; Hlasny, E.; van der Kwast, T. H.; et al. MRI-guided Focused Ultrasound Ablation for Localized Intermediate-Risk Prostate Cancer: Early Results of a Phase II Trial. *Radiology* **2021**, *298* (3), 695-703. DOI: 10.1148/radiol.2021202717
62. Rischmann, P.; Gelet, A.; Riche, B.; Villers, A.; Pasticier, G.; Bondil, P.; Jung, J. L.; Bugel, H.; Petit, J.; Toledano, H.; et al. Focal High Intensity Focused Ultrasound of Unilateral Localized Prostate Cancer: A Prospective Multicentric Hemiablation Study of 111 Patients. *Eur Urol* **2017**, *71* (2), 267-273. DOI: 10.1016/j.eururo.2016.09.039
63. Sivaraman, A.; Marra, G.; Stabile, A.; Mombet, A.; Macek, P.; Lanz, C.; Cathala, N.; Moschini, M.; Carneiro, A.; Sanchez-Salas, R.; et al. Does mpMRI guidance improve HIFU partial gland ablation compared to conventional ultrasound guidance? Early functional outcomes and complications from a single center. *Int Braz J Urol* **2020**, *46* (6), 984-992. DOI: 10.1590/s1677-5538.Ibju.2019.0682
64. van Velthoven, R.; Aoun, F.; Marcelis, Q.; Albisinni, S.; Zanaty, M.; Lemort, M.; Peltier, A.; Limani, K. A prospective clinical trial of HIFU hemiablation for clinically localized prostate cancer. *Prostate Cancer Prostatic Dis* **2016**, *19* (1), 79-83. DOI: 10.1038/pcan.2015.55
65. von Hardenberg, J.; Westhoff, N.; Baumunk, D.; Hausmann, D.; Martini, T.; Marx, A.; Porubsky, S.; Schostak, M.; Michel, M. S.; Ritter, M. Prostate cancer treatment by the latest focal HIFU device with MRI/TRUS-fusion control biopsies: A prospective evaluation. *Urol Oncol* **2018**, *36* (9), 401.e401-401.e409. DOI: 10.1016/j.urolonc.2018.05.022
66. Arnouil, N.; Gelet, A.; Matillon, X.; Rouviere, O.; Colombel, M.; Ruffion, A.; Mège-Lechevallier, F.; Subtil, F.; Badet, L.; Crouzet, S. [Focal HIFU vs robot-assisted total prostatectomy: Functionnal and oncologic outcomes at one year]. *Prog Urol* **2018**, *28* (12), 603-610. DOI: 10.1016/j.purol.2018.07.285
67. Crouzet, S.; Poissonnier, L.; Murat, F. J.; Pasticier, G.; Rouvière, O.; Mège-Lechevallier, F.; Chapelon, J. Y.; Martin, X.; Gelet, A. [Outcomes of HIFU for localised prostate cancer using the Ablatherm Integrate Imaging® device]. *Prog Urol* **2011**, *21* (3), 191-197. DOI: 10.1016/j.purol.2010.07.005
68. S, D. E. L.; Checcucci, E.; Piramide, F.; Russo, F.; Alessio, P.; Garrou, D.; Peretti, D.; Sica, M.; Volpi, G.; Piana, A.; et al. MRI/real-time ultrasound image fusion guided high-intensity focused ultrasound: a prospective comparative and functional analysis of different ablative techniques. *Minerva Urol Nephrol* **2023**, *75* (2), 172-179. DOI: 10.23736/s2724-6051.22.04853-4

69. Misraï, V.; Rouprêt, M.; Chartier-Kastler, E.; Comperat, E.; Renard-Penna, R.; Haertig, A.; Bitker, M. O.; Richard, F.; Conort, P. Oncologic control provided by HIFU therapy as single treatment in men with clinically localized prostate cancer. *World J Urol* **2008**, *26* (5), 481-485. DOI: 10.1007/s00345-008-0286-8
70. Rosenhammer, B.; Ganzer, R.; Zeman, F.; Näger, T.; Fritsche, H. M.; Blana, A.; Burger, M.; Bründl, J. Oncological long-term outcome of whole gland HIFU and open radical prostatectomy: a comparative analysis. *World J Urol* **2019**, *37* (10), 2073-2080. DOI: 10.1007/s00345-018-2613-z
71. Shoji, S.; Hiraiwa, S.; Uemura, K.; Nitta, M.; Hasegawa, M.; Kawamura, Y.; Hashida, K.; Hasebe, T.; Tajiri, T.; Miyajima, A. Focal therapy with high-intensity focused ultrasound for the localized prostate cancer for Asian based on the localization with MRI-TRUS fusion image-guided transperineal biopsy and 12-cores transperineal systematic biopsy: prospective analysis of oncological and functional outcomes. *Int J Clin Oncol* **2020**, *25* (10), 1844-1853. DOI: 10.1007/s10147-020-01723-9
72. Durán-Rivera, A.; Montoliu García, A.; Juan Escudero, J.; Garrido Abad, P.; Fernández Arjona, M.; López Alcina, E. High-intensity focused ultrasound therapy for the treatment of prostate cancer: Medium-term experience. *Actas Urol Esp (Engl Ed)* **2018**, *42* (7), 450-456. DOI: 10.1016/j.acuro.2017.11.007
73. Wu, Y. T.; Chiang, P. H. Cohort study of high-intensity focused ultrasound in the treatment of localised prostate cancer treatment: Medium-term results from a single centre. *PLoS One* **2020**, *15* (7), e0236026. DOI: 10.1371/journal.pone.0236026
74. Abreu, A. L.; Peretsman, S.; Iwata, A.; Shakir, A.; Iwata, T.; Brooks, J.; Tafuri, A.; Ashrafi, A.; Park, D.; Cacciamani, G. E.; et al. High Intensity Focused Ultrasound Hemigland Ablation for Prostate Cancer: Initial Outcomes of a United States Series. *J Urol* **2020**, *204* (4), 741-747. DOI: 10.1097/ju.0000000000001126
75. Chen, P. Y.; Chiang, P. H.; Liu, Y. Y.; Chuang, Y. C.; Cheng, Y. T. Primary whole-gland ablation for localized prostate cancer with high-intensity focused ultrasound: The important predictors of biochemical recurrence. *Int J Urol* **2018**, *25* (6), 615-620. DOI: 10.1111/iju.13581
76. Dickinson, L.; Arya, M.; Afzal, N.; Cathcart, P.; Charman, S. C.; Cornaby, A.; Hindley, R. G.; Lewi, H.; McCartan, N.; Moore, C. M.; et al. Medium-term Outcomes after Whole-gland High-intensity Focused Ultrasound for the Treatment of Nonmetastatic Prostate Cancer from a Multicentre Registry Cohort. *Eur Urol* **2016**, *70* (4), 668-674. DOI: 10.1016/j.eururo.2016.02.054
77. Reddy, D.; Peters, M.; Shah, T. T.; van Son, M.; Tanaka, M. B.; Huber, P. M.; Lomas, D.; Rakauskas, A.; Miah, S.; Eldred-Evans, D.; et al. Cancer Control Outcomes Following Focal Therapy Using High-intensity Focused Ultrasound in 1379 Men with Nonmetastatic Prostate Cancer: A Multi-institute 15-year Experience. *Eur Urol* **2022**, *81* (4), 407-413. DOI: 10.1016/j.eururo.2022.01.005
78. Komura, K.; Inamoto, T.; Takai, T.; Uchimoto, T.; Saito, K.; Tanda, N.; Kono, J.; Minami, K.; Uehara, H.; Fujisue, Y.; et al. Single session of high-intensity focused ultrasound for localized prostate cancer: treatment outcomes and potential effect as a primary therapy. *World J Urol* **2014**, *32* (5), 1339-1345. DOI: 10.1007/s00345-013-1215-z
79. Limani, K.; Aoun, F.; Holz, S.; Paesmans, M.; Peltier, A.; van Velthoven, R. Single high intensity focused ultrasound session as a whole gland primary treatment for clinically localized prostate cancer: 10-year outcomes. *Prostate Cancer* **2014**, *2014*, 186782. DOI: 10.1155/2014/186782
80. Mearini, L.; D'Urso, L.; Collura, D.; Nunzi, E.; Muto, G.; Porena, M. High-intensity focused ultrasound for the treatment of prostate cancer: A prospective trial with long-term follow-up. *Scand J Urol* **2015**, *49* (4), 267-274. DOI: 10.3109/21681805.2014.988174

81. Pfeiffer, D.; Berger, J.; Gross, A. J. Single application of high-intensity focused ultrasound as a first-line therapy for clinically localized prostate cancer: 5-year outcomes. *BJU Int* **2012**, *110* (11), 1702-1707. DOI: 10.1111/j.1464-410X.2012.11375.x
82. Ripert, T.; Azémar, M. D.; Ménard, J.; Barbe, C.; Messaoudi, R.; Bayoud, Y.; Pierrevelcin, J.; Duval, F.; Staerman, F. Six years' experience with high-intensity focused ultrasonography for prostate cancer: oncological outcomes using the new 'Stuttgart' definition for biochemical failure. *BJU Int* **2011**, *107* (12), 1899-1905. DOI: 10.1111/j.1464-410X.2010.09710.x
83. Tsai, M. Y.; Lin, C. T.; Chiang, P. H.; Chiang, P. H.; Chiang, P. C. High-Intensity Focused Ultrasound (Sonablate(®)) for Prostate Cancer: Preliminary Outcomes in Taiwan. *Ann Surg Oncol* **2023**, *30* (13), 8764-8769. DOI: 10.1245/s10434-023-14250-4
84. Ploussard, G.; Coloby, P.; Chevallier, T.; Occéan, B. V.; Houédé, N.; Villers, A.; Rischmann, P. Whole-gland or Subtotal High-intensity Focused Ultrasound Versus Radical Prostatectomy: The Prospective, Noninferiority, Nonrandomized HIFI Trial. *Eur Urol* **2025**, *87* (5), 526-533. DOI: 10.1016/j.eururo.2024.11.006
85. Nahar, B.; Ajami, T.; Williams, A.; Soodana Prakash, N.; Khandekar, A.; Freitas, P. F. S.; Malpani, A.; Rayan, J.; Sureshkumar, K.; Ritch, C. R.; et al. Survival Outcomes and Recurrence Patterns Following Focal High-intensity Focused Ultrasound Treatment for Localized Prostate Cancer: Insights on Patient Selection and Lessons Learned. *Eur Urol Focus* **2024**. DOI: 10.1016/j.euf.2024.11.005
